# Supplementary material for: Resolving the full spectrum of human genome variation using Linked-Reads
Source: Genome Res. 2019 Apr;29(4):635–45. doi: 10.1101/gr.234443.118 (PMC6442396; doi:10.1101/gr.234443.118)
Supplement: Supplemental Material [file supp_gr.234443.118_Supplemental_longranger-2.2.zip › longranger-2.2.tar.bz2!/lr2.2-public/lib/stan/cmdstan/stan/doc/api/html/namespacestan_1_1math.html]

Stan: stan::math Namespace Reference


|  |  |
| --- | --- |
|  | Stan  2.5.0  probability, sampling & optimization |


- Main Page
- Related Pages
- Namespaces
- Classes
- Files

- Namespace List
- Namespace Members

All Classes Namespaces Files Functions Variables Typedefs Enumerations Enumerator Friends Macros Pages

- stan
- math

Namespaces |
Classes |
Typedefs |
Functions |
Variables

stan::math Namespace Reference

Matrices and templated mathematical functions.
More...

|  |  |
| --- | --- |
| Namespaces | |
|  | detail |
|  | |
|  | policies |
|  | Extending boost functionality. |
|  | |

|  |  |
| --- | --- |
| Classes | |
| class | LDLT\_factor< stan::agrad::var, R, C > |
|  | A template specialization of src/stan/math/matrix/LDLT\_factor.hpp for stan::agrad::var which can be used with all the \*\_ldlt functions. More... |
|  | |
| struct | coupled\_ode\_system< F, double, stan::agrad::var > |
|  | The coupled ODE system for known initial values and unknown parameters. More... |
|  | |
| struct | coupled\_ode\_system< F, stan::agrad::var, double > |
|  | The coupled ODE system for unknown initial values and known parameters. More... |
|  | |
| struct | coupled\_ode\_system< F, stan::agrad::var, stan::agrad::var > |
|  | The coupled ode system for unknown intial values and unknown parameters. More... |
|  | |
| struct | promote\_scalar\_struct |
|  | General struct to hold static function for promoting underlying scalar types. More... |
|  | |
| struct | promote\_scalar\_struct< T, T > |
|  | Struct to hold static function for promoting underlying scalar types. More... |
|  | |
| struct | promote\_scalar\_struct< T, std::vector< S > > |
|  | Struct to hold static function for promoting underlying scalar types. More... |
|  | |
| struct | promote\_scalar\_type |
|  | Template metaprogram to calculate a type for converting a convertible type. More... |
|  | |
| struct | promote\_scalar\_type< T, std::vector< S > > |
|  | Template metaprogram to calculate a type for a container whose underlying scalar is converted from the second template parameter type to the first. More... |
|  | |
| class | accumulator |
|  | Class to accumulate values and eventually return their sum. More... |
|  | |
| struct | array\_builder |
|  | Structure for building up arrays in an expression (rather than in statements) using an argumentchaining add() method and a getter method array() to return the result. More... |
|  | |
| struct | common\_type |
|  | |
| struct | common\_type< std::vector< T1 >, std::vector< T2 > > |
|  | |
| struct | common\_type< Eigen::Matrix< T1, R, C >, Eigen::Matrix< T2, R, C > > |
|  | |
| class | LDLT\_factor |
|  | |
| class | LDLT\_factor< T, R, C > |
|  | LDLT\_factor is a thin wrapper on Eigen::LDLT to allow for reusing factorizations and efficient autodiff of things like log determinants and solutions to linear systems. More... |
|  | |
| struct | index\_type< Eigen::Matrix< T, R, C > > |
|  | Template metaprogram defining typedef for the type of index for an Eigen matrix, vector, or row vector. More... |
|  | |
| struct | value\_type< Eigen::Matrix< T, R, C > > |
|  | Template metaprogram defining the type of values stored in an Eigen matrix, vector, or row vector. More... |
|  | |
| struct | promote\_scalar\_struct< T, Eigen::Matrix< S,-1,-1 > > |
|  | Struct to hold static function for promoting underlying scalar types. More... |
|  | |
| struct | promote\_scalar\_struct< T, Eigen::Matrix< S, 1,-1 > > |
|  | Struct to hold static function for promoting underlying scalar types. More... |
|  | |
| struct | promote\_scalar\_struct< T, Eigen::Matrix< S,-1, 1 > > |
|  | Struct to hold static function for promoting underlying scalar types. More... |
|  | |
| struct | promote\_scalar\_type< T, Eigen::Matrix< S, Eigen::Dynamic, Eigen::Dynamic > > |
|  | Template metaprogram to calculate a type for a matrix whose underlying scalar is converted from the second template parameter type to the first. More... |
|  | |
| struct | promote\_scalar\_type< T, Eigen::Matrix< S, Eigen::Dynamic, 1 > > |
|  | Template metaprogram to calculate a type for a vector whose underlying scalar is converted from the second template parameter type to the first. More... |
|  | |
| struct | promote\_scalar\_type< T, Eigen::Matrix< S, 1, Eigen::Dynamic > > |
|  | Template metaprogram to calculate a type for a row vector whose underlying scalar is converted from the second template parameter type to the first. More... |
|  | |
| struct | promoter |
|  | |
| struct | promoter< T, T > |
|  | |
| struct | promoter< std::vector< F >, std::vector< T > > |
|  | |
| struct | promoter< std::vector< T >, std::vector< T > > |
|  | |
| struct | promoter< Eigen::Matrix< F, R, C >, Eigen::Matrix< T, R, C > > |
|  | |
| struct | promoter< Eigen::Matrix< T, R, C >, Eigen::Matrix< T, R, C > > |
|  | |
| struct | index\_type |
|  | Primary template class for the metaprogram to compute the index type of a container. More... |
|  | |
| struct | index\_type< const T > |
|  | Template class for metaprogram to compute the type of indexes used in a constant container type. More... |
|  | |
| struct | index\_type< std::vector< T > > |
|  | Template metaprogram class to compute the type of index for a standard vector. More... |
|  | |
| struct | value\_type |
|  | Primary template class for metaprogram to compute the type of values stored in a container. More... |
|  | |
| struct | value\_type< const T > |
|  | Template class for metaprogram to compute the type of values stored in a constant container. More... |
|  | |
| struct | value\_type< std::vector< T > > |
|  | Template metaprogram class to compute the type of values stored in a standard vector. More... |
|  | |
| struct | coupled\_ode\_observer |
|  | Observer for the coupled states. More... |
|  | |
| struct | coupled\_ode\_system |
|  | Base template class for a coupled ordinary differential equation system, which adds sensitivities to the base system. More... |
|  | |
| struct | coupled\_ode\_system< F, double, double > |
|  | The coupled ode system for known initial values and known parameters. More... |
|  | |
| struct | store\_type |
|  | |
| struct | store\_type< double > |
|  | |
| struct | store\_type< int > |
|  | |
| struct | pass\_type |
|  | |
| struct | pass\_type< double > |
|  | |
| struct | pass\_type< int > |
|  | |
| class | seq\_view |
|  | |
| class | seq\_view< T, Eigen::Matrix< S, Eigen::Dynamic, 1 > > |
|  | |
| class | seq\_view< T, Eigen::Matrix< S, 1, Eigen::Dynamic > > |
|  | |
| class | seq\_view< T, Eigen::Matrix< S, Eigen::Dynamic, Eigen::Dynamic > > |
|  | |
| class | seq\_view< T, std::vector< S > > |
|  | |
| class | seq\_view< T, std::vector< T > > |
|  | |
| class | seq\_view< T, std::vector< std::vector< T > > > |
|  | |
| class | seq\_view< double, std::vector< int > > |
|  | |

|  |  |
| --- | --- |
| Typedefs | |
| typedef index\_type  < Eigen::Matrix< double,   Eigen::Dynamic, Eigen::Dynamic >  >::type | size\_type |
|  | Type for sizes and indexes in an Eigen matrix with double e. More... |
|  | |
| typedef Eigen::Matrix< double,   Eigen::Dynamic, Eigen::Dynamic > | matrix\_d |
|  | Type for matrix of double values. More... |
|  | |
| typedef Eigen::Matrix< double,   Eigen::Dynamic, 1 > | vector\_d |
|  | Type for (column) vector of double values. More... |
|  | |
| typedef Eigen::Matrix< double,   1, Eigen::Dynamic > | row\_vector\_d |
|  | Type for (row) vector of double values. More... |
|  | |

|  |  |
| --- | --- |
| Functions | |
| void | add\_initial\_values (const std::vector< stan::agrad::var > &y0, std::vector< std::vector< stan::agrad::var > > &y) |
|  | Increment the state derived from the coupled system in the with the original initial state. More... |
|  | |
| double | pi () |
|  | Return the value of pi. More... |
|  | |
| double | e () |
|  | Return the base of the natural logarithm. More... |
|  | |
| double | sqrt2 () |
|  | Return the square root of two. More... |
|  | |
| double | log10 () |
|  | Return natural logarithm of ten. More... |
|  | |
| double | positive\_infinity () |
|  | Return positive infinity. More... |
|  | |
| double | negative\_infinity () |
|  | Return negative infinity. More... |
|  | |
| double | not\_a\_number () |
|  | Return (quiet) not-a-number. More... |
|  | |
| double | machine\_precision () |
|  | Returns the difference between 1.0 and the next value representable. More... |
|  | |
| template<typename T\_y , typename T\_low , typename T\_high , typename T\_result > | |
| bool | check\_bounded (const char \*function, const T\_y &y, const T\_low &low, const T\_high &high, const char \*name, T\_result \*result) |
|  | |
| template<typename T , typename T\_result > | |
| bool | check\_consistent\_size (size\_t max\_size, const char \*function, const T &x, const char \*name, T\_result \*result) |
|  | |
| template<typename T1 , typename T2 , typename T\_result > | |
| bool | check\_consistent\_sizes (const char \*function, const T1 &x1, const T2 &x2, const char \*name1, const char \*name2, T\_result \*result) |
|  | |
| template<typename T1 , typename T2 , typename T3 , typename T\_result > | |
| bool | check\_consistent\_sizes (const char \*function, const T1 &x1, const T2 &x2, const T3 &x3, const char \*name1, const char \*name2, const char \*name3, T\_result \*result) |
|  | |
| template<typename T1 , typename T2 , typename T3 , typename T4 , typename T\_result > | |
| bool | check\_consistent\_sizes (const char \*function, const T1 &x1, const T2 &x2, const T3 &x3, const T4 &x4, const char \*name1, const char \*name2, const char \*name3, const char \*name4, T\_result \*result) |
|  | |
| template<typename T\_y , typename T\_eq , typename T\_result > | |
| bool | check\_equal (const char \*function, const T\_y &y, const T\_eq &eq, const char \*name, T\_result \*result) |
|  | |
| template<typename T\_y , typename T\_result > | |
| bool | check\_finite (const char \*function, const T\_y &y, const char \*name, T\_result \*result) |
|  | Checks if the variable y is finite. More... |
|  | |
| template<typename T\_y , typename T\_low , typename T\_result > | |
| bool | check\_greater (const char \*function, const T\_y &y, const T\_low &low, const char \*name, T\_result \*result) |
|  | |
| template<typename T\_y , typename T\_low , typename T\_result > | |
| bool | check\_greater\_or\_equal (const char \*function, const T\_y &y, const T\_low &low, const char \*name, T\_result \*result) |
|  | |
| template<typename T\_y , typename T\_high , typename T\_result > | |
| bool | check\_less (const char \*function, const T\_y &y, const T\_high &high, const char \*name, T\_result \*result) |
|  | |
| template<typename T\_y , typename T\_high , typename T\_result > | |
| bool | check\_less\_or\_equal (const char \*function, const T\_y &y, const T\_high &high, const char \*name, T\_result \*result) |
|  | |
| template<typename T\_y , typename T\_result > | |
| bool | check\_nonnegative (const char \*function, const T\_y &y, const char \*name, T\_result \*result) |
|  | |
| template<typename T\_y , typename T\_result > | |
| bool | check\_not\_nan (const char \*function, const T\_y &y, const char \*name, T\_result \*result) |
|  | Checks if the variable y is nan. More... |
|  | |
| template<typename T\_y , typename T\_result > | |
| bool | check\_positive (const char \*function, const T\_y &y, const char \*name, T\_result \*result) |
|  | |
| template<typename T\_y , typename T\_result > | |
| bool | check\_positive\_finite (const char \*function, const T\_y &y, const char \*name, T\_result \*result) |
|  | |
| template<typename T , typename T\_result , typename T\_msg > | |
| bool | dom\_err (const char \*function, const T &y, const char \*name, const char \*error\_msg, const T\_msg error\_msg2, T\_result \*result) |
|  | |
| template<typename T , typename T\_result , typename T\_msg > | |
| bool | dom\_err\_vec (const size\_t i, const char \*function, const T &y, const char \*name, const char \*error\_msg, const T\_msg error\_msg2, T\_result \*result) |
|  | |
| template<typename T\_y , typename T\_result > | |
| bool | check\_cholesky\_factor (const char \*function, const Eigen::Matrix< T\_y, Eigen::Dynamic, Eigen::Dynamic > &y, const char \*name, T\_result \*result) |
|  | Return `true` if the specified matrix is a valid Cholesky factor. More... |
|  | |
| template<typename T\_y , typename T\_result > | |
| bool | check\_cholesky\_factor\_corr (const char \*function, const Eigen::Matrix< T\_y, Eigen::Dynamic, Eigen::Dynamic > &y, const char \*name, T\_result \*result) |
|  | Return `true` if the specified matrix is a valid Cholesky factor. More... |
|  | |
| template<typename T > | |
| bool | check\_cholesky\_factor\_corr (const char \*function, const Eigen::Matrix< T, Eigen::Dynamic, Eigen::Dynamic > &y, const char \*name, T \*result=0) |
|  | |
| template<typename T\_y , typename T\_result > | |
| bool | check\_cholesky\_factor\_corr (const char \*function, const Eigen::Matrix< T\_y, Eigen::Dynamic, Eigen::Dynamic > &y, T\_result \*result) |
|  | Return `true` if the specified matrix is a valid Cholesky factor (lower triangular, positive diagonal). More... |
|  | |
| template<typename T > | |
| bool | check\_cholesky\_factor\_corr (const char \*function, const Eigen::Matrix< T, Eigen::Dynamic, Eigen::Dynamic > &y, T \*result=0) |
|  | |
| template<typename T\_y , typename T\_result , int R, int C> | |
| bool | check\_column\_index (const char \*function, size\_t i, const Eigen::Matrix< T\_y, R, C > &y, const char \*name, T\_result \*result) |
|  | Return `true` if the specified index is a valid column of the matrix. More... |
|  | |
| template<typename T\_y , typename T\_result > | |
| bool | check\_corr\_matrix (const char \*function, const Eigen::Matrix< T\_y, Eigen::Dynamic, Eigen::Dynamic > &y, const char \*name, T\_result \*result) |
|  | Return `true` if the specified matrix is a valid correlation matrix. More... |
|  | |
| template<typename T\_y , typename T\_result > | |
| bool | check\_cov\_matrix (const char \*function, const Eigen::Matrix< T\_y, Eigen::Dynamic, Eigen::Dynamic > &y, const char \*name, T\_result \*result) |
|  | Return `true` if the specified matrix is a valid covariance matrix. More... |
|  | |
| template<typename T , int R, int C, typename T\_result > | |
| bool | check\_ldlt\_factor (const char \*function, stan::math::LDLT\_factor< T, R, C > &A, const char \*name, T\_result \*result) |
|  | Return `true` if the underlying matrix is positive definite. More... |
|  | |
| template<typename T\_y , typename T\_result > | |
| bool | check\_lower\_triangular (const char \*function, const Eigen::Matrix< T\_y, Eigen::Dynamic, Eigen::Dynamic > &y, const char \*name, T\_result \*result) |
|  | Return `true` if the specified matrix is lower triangular. More... |
|  | |
| template<typename T1 , typename T2 , int R1, int C1, int R2, int C2, typename T\_result > | |
| bool | check\_matching\_dims (const char \*function, const Eigen::Matrix< T1, R1, C1 > &y1, const char \*name1, const Eigen::Matrix< T2, R2, C2 > &y2, const char \*name2, T\_result \*result) |
|  | |
| template<typename T\_y1 , typename T\_y2 , typename T\_result > | |
| bool | check\_matching\_sizes (const char \*function, const T\_y1 &y1, const char \*name1, const T\_y2 &y2, const char \*name2, T\_result \*result) |
|  | |
| template<typename T1 , typename T2 , typename T\_result > | |
| bool | check\_multiplicable (const char \*function, const T1 &y1, const char \*name1, const T2 &y2, const char \*name2, T\_result \*result) |
|  | |
| template<typename T\_y , typename T\_result > | |
| bool | check\_nonzero\_size (const char \*function, const T\_y &y, const char \*name, T\_result \*result) |
|  | Return `true` if the specified matrix/vector is of non-zero size. More... |
|  | |
| template<typename T\_y , typename T\_result > | |
| bool | check\_ordered (const char \*function, const Eigen::Matrix< T\_y, Eigen::Dynamic, 1 > &y, const char \*name, T\_result \*result) |
|  | Return `true` if the specified vector is sorted into increasing order. More... |
|  | |
| template<typename T\_y , typename T\_result > | |
| bool | check\_ordered (const char \*function, const std::vector< T\_y > &y, const char \*name, T\_result \*result) |
|  | |
| template<typename T > | |
| bool | check\_ordered (const char \*function, const Eigen::Matrix< T, Eigen::Dynamic, 1 > &y, const char \*name, T \*result=0) |
|  | |
| template<typename T > | |
| bool | check\_ordered (const char \*function, const std::vector< T > &y, const char \*name, T \*result=0) |
|  | |
| template<typename T\_y , typename T\_result > | |
| bool | check\_pos\_definite (const char \*function, const Eigen::Matrix< T\_y, Eigen::Dynamic, Eigen::Dynamic > &y, const char \*name, T\_result \*result) |
|  | Return `true` if the specified matrix is positive definite. More... |
|  | |
| template<typename T\_y , typename T\_result > | |
| bool | check\_pos\_semidefinite (const char \*function, const Eigen::Matrix< T\_y, Eigen::Dynamic, Eigen::Dynamic > &y, const char \*name, T\_result \*result) |
|  | Return `true` if the specified matrix is positive definite. More... |
|  | |
| template<typename T\_y , typename T\_result > | |
| bool | check\_positive\_ordered (const char \*function, const Eigen::Matrix< T\_y, Eigen::Dynamic, 1 > &y, const char \*name, T\_result \*result) |
|  | Return `true` if the specified vector contains only non-negative values and is sorted into increasing order. More... |
|  | |
| void | check\_range (size\_t max, size\_t i, const char \*msg, size\_t idx) |
|  | |
| template<typename T\_y , typename T\_result , int R, int C> | |
| bool | check\_row\_index (const char \*function, size\_t i, const Eigen::Matrix< T\_y, R, C > &y, const char \*name, T\_result \*result) |
|  | Return `true` if the specified index is a valid row of the matrix. More... |
|  | |
| template<typename T\_prob , typename T\_result > | |
| bool | check\_simplex (const char \*function, const Eigen::Matrix< T\_prob, Eigen::Dynamic, 1 > &theta, const char \*name, T\_result \*result) |
|  | Return `true` if the specified vector is simplex. More... |
|  | |
| template<typename T\_size1 , typename T\_size2 , typename T\_result > | |
| bool | check\_size\_match (const char \*function, T\_size1 i, const char \*name\_i, T\_size2 j, const char \*name\_j, T\_result \*result) |
|  | |
| template<typename T\_y , typename T\_result > | |
| bool | check\_spsd\_matrix (const char \*function, const Eigen::Matrix< T\_y, Eigen::Dynamic, Eigen::Dynamic > &y, const char \*name, T\_result \*result) |
|  | Return `true` if the specified matrix is a square, symmetric, and positive semi-definite. More... |
|  | |
| template<typename T\_y , typename T\_result > | |
| bool | check\_square (const char \*function, const Eigen::Matrix< T\_y, Eigen::Dynamic, Eigen::Dynamic > &y, const char \*name, T\_result \*result) |
|  | Return `true` if the specified matrix is square. More... |
|  | |
| template<typename T\_y , typename T\_result > | |
| bool | check\_std\_vector\_index (const char \*function, size\_t i, const std::vector< T\_y > &y, const char \*name, T\_result \*result) |
|  | Return `true` if the specified index is valid in std vector. More... |
|  | |
| template<typename T\_y , typename T\_result > | |
| bool | check\_symmetric (const char \*function, const Eigen::Matrix< T\_y, Eigen::Dynamic, Eigen::Dynamic > &y, const char \*name, T\_result \*result) |
|  | Return `true` if the specified matrix is symmetric. More... |
|  | |
| template<typename T\_prob , typename T\_result > | |
| bool | check\_unit\_vector (const char \*function, const Eigen::Matrix< T\_prob, Eigen::Dynamic, 1 > &theta, const char \*name, T\_result \*result) |
|  | Return `true` if the specified vector is unit vector. More... |
|  | |
| template<typename T > | |
| bool | check\_unit\_vector (const char \*function, const Eigen::Matrix< T, Eigen::Dynamic, 1 > &theta, const char \*name, T \*result=0) |
|  | |
| template<typename T , int R, int C, typename T\_result > | |
| bool | check\_vector (const char \*function, const Eigen::Matrix< T, R, C > &x, const char \*name, T\_result \*result) |
|  | |
| void | validate\_non\_negative\_index (const std::string &var\_name, const std::string &expr, int val) |
|  | |
| double | abs (double x) |
|  | Return floating-point absolute value. More... |
|  | |
| template<typename T > | |
| bool | as\_bool (const T x) |
|  | Return 1 if the argument is unequal to zero and 0 otherwise. More... |
|  | |
| template<typename T2 > | |
| T2 | bessel\_first\_kind (const int v, const T2 z) |
|  | More... |
|  | |
| template<typename T2 > | |
| T2 | bessel\_second\_kind (const int v, const T2 z) |
|  | More... |
|  | |
| template<typename T > | |
| boost::math::tools::promote\_args  < T >::type | binary\_log\_loss (const int y, const T y\_hat) |
|  | Returns the log loss function for binary classification with specified reference and response values. More... |
|  | |
| template<typename T\_N , typename T\_n > | |
| boost::math::tools::promote\_args  < T\_N, T\_n >::type | binomial\_coefficient\_log (const T\_N N, const T\_n n) |
|  | Return the log of the binomial coefficient for the specified arguments. More... |
|  | |
| double | digamma (double x) |
|  | More... |
|  | |
| double | dist (const std::vector< double > &x, const std::vector< double > &y) |
|  | |
| int | divide (const int x, const int y) |
|  | |
| double | dot (const std::vector< double > &x, const std::vector< double > &y) |
|  | |
| double | dot\_self (const std::vector< double > &x) |
|  | |
| template<typename T > | |
| boost::math::tools::promote\_args  < T >::type | exp2 (const T y) |
|  | Return the exponent base 2 of the specified argument (C99). More... |
|  | |
| template<typename T1 , typename T2 > | |
| boost::math::tools::promote\_args  < T1, T2 >::type | falling\_factorial (const T1 x, const T2 n) |
|  | More... |
|  | |
| template<typename T1 , typename T2 > | |
| boost::math::tools::promote\_args  < T1, T2 >::type | fdim (T1 a, T2 b) |
|  | The positive difference function (C99). More... |
|  | |
| double | gamma\_p (double x, double a) |
|  | More... |
|  | |
| double | gamma\_q (double x, double a) |
|  | More... |
|  | |
| double | ibeta (const double a, const double b, const double x) |
|  | The normalized incomplete beta function of a, b, and x. More... |
|  | |
| template<typename T\_true , typename T\_false > | |
| boost::math::tools::promote\_args  < T\_true, T\_false >::type | if\_else (const bool c, const T\_true y\_true, const T\_false y\_false) |
|  | Return the second argument if the first argument is true and otherwise return the second argument. More... |
|  | |
| template<typename T > | |
| unsigned int | int\_step (const T y) |
|  | The integer step, or Heaviside, function. More... |
|  | |
| template<typename T > | |
| boost::math::tools::promote\_args  < T >::type | inv (const T x) |
|  | |
| template<typename T > | |
| boost::math::tools::promote\_args  < T >::type | inv\_cloglog (T x) |
|  | The inverse complementary log-log function. More... |
|  | |
| template<typename T > | |
| boost::math::tools::promote\_args  < T >::type | inv\_logit (const T a) |
|  | Returns the inverse logit function applied to the argument. More... |
|  | |
| template<typename T > | |
| boost::math::tools::promote\_args  < T >::type | inv\_sqrt (const T x) |
|  | |
| template<typename T > | |
| boost::math::tools::promote\_args  < T >::type | inv\_square (const T x) |
|  | |
| template<typename Vector > | |
| void | inverse\_softmax (const Vector &simplex, Vector &y) |
|  | Writes the inverse softmax of the simplex argument into the second argument. More... |
|  | |
| int | is\_inf (const double x) |
|  | Returns 1 if the input is infinite and 0 otherwise. More... |
|  | |
| int | is\_nan (const double x) |
|  | Returns 1 if the input is NaN and 0 otherwise. More... |
|  | |
| template<typename T > | |
| bool | is\_uninitialized (T x) |
|  | Returns `true` if the specified variable is uninitialized. More... |
|  | |
| template<typename T1 , typename T2 > | |
| boost::math::tools::promote\_args  < T1, T2 >::type | lbeta (const T1 a, const T2 b) |
|  | Return the log of the beta function applied to the specified arguments. More... |
|  | |
| double | lgamma (double x) |
|  | More... |
|  | |
| template<typename T > | |
| boost::math::tools::promote\_args  < T >::type | lmgamma (const int k, T x) |
|  | Return the natural logarithm of the multivariate gamma function with the speciifed dimensions and argument. More... |
|  | |
| template<typename T > | |
| boost::math::tools::promote\_args  < T >::type | log1m (T x) |
|  | Return the natural logarithm of one minus the specified value. More... |
|  | |
| template<typename T > | |
| boost::math::tools::promote\_args  < T >::type | log1m\_exp (const T a) |
|  | Calculates the log of 1 minus the exponential of the specified value without overflow log1m\_exp(x) = log(1-exp(x)). More... |
|  | |
| template<typename T > | |
| boost::math::tools::promote\_args  < T >::type | log1m\_inv\_logit (const T u) |
|  | Returns the natural logarithm of 1 minus the inverse logit of the specified argument. More... |
|  | |
| template<typename T > | |
| boost::math::tools::promote\_args  < T >::type | log1p (const T x) |
|  | Return the natural logarithm of one plus the specified value. More... |
|  | |
| template<typename T > | |
| boost::math::tools::promote\_args  < T >::type | log1p\_exp (const T a) |
|  | Calculates the log of 1 plus the exponential of the specified value without overflow. More... |
|  | |
| template<typename T > | |
| boost::math::tools::promote\_args  < T >::type | log2 (const T a) |
|  | Returns the base 2 logarithm of the argument (C99). More... |
|  | |
| double | log2 () |
|  | Return natural logarithm of two. More... |
|  | |
| template<typename T1 , typename T2 > | |
| boost::math::tools::promote\_args  < T1, T2 >::type | log\_diff\_exp (const T1 x, const T2 y) |
|  | The natural logarithm of the difference of the natural exponentiation of x1 and the natural exponentiation of x2. More... |
|  | |
| template<typename T1 , typename T2 > | |
| boost::math::tools::promote\_args  < T1, T2 >::type | log\_falling\_factorial (const T1 x, const T2 n) |
|  | More... |
|  | |
| template<typename T > | |
| boost::math::tools::promote\_args  < T >::type | log\_inv\_logit (const T &u) |
|  | Returns the natural logarithm of the inverse logit of the specified argument. More... |
|  | |
| template<typename T1 , typename T2 > | |
| boost::math::tools::promote\_args  < T1, T2 >::type | log\_rising\_factorial (const T1 x, const T2 n) |
|  | More... |
|  | |
| template<typename T1 , typename T2 > | |
| boost::math::tools::promote\_args  < T1, T2 >::type | log\_sum\_exp (const T2 &a, const T1 &b) |
|  | Calculates the log sum of exponetials without overflow. More... |
|  | |
| double | log\_sum\_exp (const std::vector< double > &x) |
|  | Return the log of the sum of the exponentiated values of the specified sequence of values. More... |
|  | |
| template<typename T1 , typename T2 > | |
| int | logical\_and (const T1 x1, const T2 x2) |
|  | The logical and function which returns 1 if both arguments are unequal to zero and 0 otherwise. More... |
|  | |
| template<typename T1 , typename T2 > | |
| int | logical\_eq (const T1 x1, const T2 x2) |
|  | Return 1 if the first argument is equal to the second. More... |
|  | |
| template<typename T1 , typename T2 > | |
| int | logical\_gt (const T1 x1, const T2 x2) |
|  | Return 1 if the first argument is strictly greater than the second. More... |
|  | |
| template<typename T1 , typename T2 > | |
| int | logical\_gte (const T1 x1, const T2 x2) |
|  | Return 1 if the first argument is greater than or equal to the second. More... |
|  | |
| template<typename T1 , typename T2 > | |
| int | logical\_lt (T1 x1, T2 x2) |
|  | Return 1 if the first argument is strictly less than the second. More... |
|  | |
| template<typename T1 , typename T2 > | |
| int | logical\_lte (const T1 x1, const T2 x2) |
|  | Return 1 if the first argument is less than or equal to the second. More... |
|  | |
| template<typename T > | |
| int | logical\_negation (const T x) |
|  | The logical negation function which returns 1 if the input is equal to zero and 0 otherwise. More... |
|  | |
| template<typename T1 , typename T2 > | |
| int | logical\_neq (const T1 x1, const T2 x2) |
|  | Return 1 if the first argument is unequal to the second. More... |
|  | |
| template<typename T1 , typename T2 > | |
| int | logical\_or (T1 x1, T2 x2) |
|  | The logical or function which returns 1 if either argument is unequal to zero and 0 otherwise. More... |
|  | |
| template<typename T > | |
| boost::math::tools::promote\_args  < T >::type | logit (const T a) |
|  | Returns the logit function applied to the argument. More... |
|  | |
| double | max (const double a, const double b) |
|  | |
| double | min (const double a, const double b) |
|  | |
| template<typename T2 > | |
| T2 | modified\_bessel\_first\_kind (const int v, const T2 z) |
|  | More... |
|  | |
| template<typename T2 > | |
| T2 | modified\_bessel\_second\_kind (const int v, const T2 z) |
|  | More... |
|  | |
| int | modulus (const int x, const int y) |
|  | |
| template<typename T\_a , typename T\_b > | |
| boost::math::tools::promote\_args  < T\_a, T\_b >::type | multiply\_log (const T\_a a, const T\_b b) |
|  | Calculated the value of the first argument times log of the second argument while behaving properly with 0 inputs. More... |
|  | |
| template<typename T1 , typename T2 > | |
| boost::math::tools::promote\_args  < T1, T2 >::type | owens\_t (const T1 &h, const T2 &a) |
|  | The Owen's T function of h and a. More... |
|  | |
| template<typename T > | |
| boost::math::tools::promote\_args  < T >::type | Phi (const T x) |
|  | The unit normal cumulative distribution function. More... |
|  | |
| template<typename T > | |
| boost::math::tools::promote\_args  < T >::type | Phi\_approx (T x) |
|  | Approximation of the unit normal CDF. More... |
|  | |
| template<typename T > | |
| boost::enable\_if  < boost::is\_arithmetic< T >, T >  ::type | primitive\_value (T x) |
|  | Return the value of the specified arithmetic argument unmodified with its own declared type. More... |
|  | |
| template<typename T > | |
| boost::disable\_if  < boost::is\_arithmetic< T >  , double >::type | primitive\_value (const T &x) |
|  | Return the primitive value of the specified argument. More... |
|  | |
| template<typename T , typename S > | |
| promote\_scalar\_type< T, S >::type | promote\_scalar (const S &x) |
|  | This is the top-level function to call to promote the scalar types of an input of type S to type T. More... |
|  | |
| template<typename T1 , typename T2 > | |
| boost::math::tools::promote\_args  < T1, T2 >::type | rising\_factorial (const T1 x, const T2 n) |
|  | More... |
|  | |
| void | scaled\_add (std::vector< double > &x, const std::vector< double > &y, const double lambda) |
|  | |
| template<typename T > | |
| int | sign (const T &z) |
|  | |
| template<typename T > | |
| T | square (const T x) |
|  | Return the square of the specified argument. More... |
|  | |
| template<typename T > | |
| int | step (const T y) |
|  | The step, or Heaviside, function. More... |
|  | |
| void | sub (std::vector< double > &x, std::vector< double > &y, std::vector< double > &result) |
|  | |
| double | sum (std::vector< double > &x) |
|  | |
| template<typename T > | |
| T | trigamma (T x) |
|  | More... |
|  | |
| template<typename T > | |
| double | value\_of (const T x) |
|  | Return the value of the specified scalar argument converted to a double value. More... |
|  | |
| template<> | |
| double | value\_of< double > (const double x) |
|  | Return the specified argument. More... |
|  | |
| template<typename T1 , typename T2 , int R, int C> | |
| Eigen::Matrix< typename   boost::math::tools::promote\_args  < T1, T2 >::type, R, C > | add (const Eigen::Matrix< T1, R, C > &m1, const Eigen::Matrix< T2, R, C > &m2) |
|  | Return the sum of the specified matrices. More... |
|  | |
| template<typename T1 , typename T2 , int R, int C> | |
| Eigen::Matrix< typename   boost::math::tools::promote\_args  < T1, T2 >::type, R, C > | add (const Eigen::Matrix< T1, R, C > &m, const T2 &c) |
|  | Return the sum of the specified matrix and specified scalar. More... |
|  | |
| template<typename T1 , typename T2 , int R, int C> | |
| Eigen::Matrix< typename   boost::math::tools::promote\_args  < T1, T2 >::type, R, C > | add (const T1 &c, const Eigen::Matrix< T2, R, C > &m) |
|  | Return the sum of the specified scalar and specified matrix. More... |
|  | |
| template<typename T1 , typename T2 , int R1, int C1, int R2, int C2> | |
| Matrix< typename return\_type  < T1, T2 >::type, Dynamic,   Dynamic > | append\_col (const Matrix< T1, R1, C1 > &A, const Matrix< T2, R2, C2 > &B) |
|  | |
| template<typename T1 , typename T2 , int C1, int C2> | |
| Matrix< typename return\_type  < T1, T2 >::type, 1, Dynamic > | append\_col (const Matrix< T1, 1, C1 > &A, const Matrix< T2, 1, C2 > &B) |
|  | |
| template<typename T , int R1, int C1, int R2, int C2> | |
| Matrix< T, Dynamic, Dynamic > | append\_col (const Matrix< T, R1, C1 > &A, const Matrix< T, R2, C2 > &B) |
|  | |
| template<typename T , int C1, int C2> | |
| Matrix< T, 1, Dynamic > | append\_col (const Matrix< T, 1, C1 > &A, const Matrix< T, 1, C2 > &B) |
|  | |
| template<typename T1 , typename T2 , int R1, int C1, int R2, int C2> | |
| Matrix< typename return\_type  < T1, T2 >::type, Dynamic,   Dynamic > | append\_row (const Matrix< T1, R1, C1 > &A, const Matrix< T2, R2, C2 > &B) |
|  | |
| template<typename T1 , typename T2 , int R1, int R2> | |
| Matrix< typename return\_type  < T1, T2 >::type, Dynamic, 1 > | append\_row (const Matrix< T1, R1, 1 > &A, const Matrix< T2, R1, 1 > &B) |
|  | |
| template<typename T , int R1, int C1, int R2, int C2> | |
| Matrix< T, Dynamic, Dynamic > | append\_row (const Matrix< T, R1, C1 > &A, const Matrix< T, R2, C2 > &B) |
|  | |
| template<typename T , int R1, int R2> | |
| Matrix< T, Dynamic, 1 > | append\_row (const Matrix< T, R1, 1 > &A, const Matrix< T, R1, 1 > &B) |
|  | |
| template<typename LHS , typename RHS > | |
| void | assign (LHS &lhs, const RHS &rhs) |
|  | Copy the right-hand side's value to the left-hand side variable. More... |
|  | |
| template<typename LHS , typename RHS , int R1, int C1, int R2, int C2> | |
| void | assign (Eigen::Matrix< LHS, R1, C1 > &x, const Eigen::Matrix< RHS, R2, C2 > &y) |
|  | Copy the right-hand side's value to the left-hand side variable. More... |
|  | |
| template<typename LHS , typename RHS , int R, int C> | |
| void | assign (Eigen::Matrix< LHS, R, C > &x, const Eigen::Matrix< RHS, R, C > &y) |
|  | Copy the right-hand side's value to the left-hand side variable. More... |
|  | |
| template<typename LHS , typename RHS , int R, int C> | |
| void | assign (Eigen::Block< LHS > x, const Eigen::Matrix< RHS, R, C > &y) |
|  | Copy the right-hand side's value to the left-hand side variable. More... |
|  | |
| template<typename LHS , typename RHS > | |
| void | assign (std::vector< LHS > &x, const std::vector< RHS > &y) |
|  | Copy the right-hand side's value to the left-hand side variable. More... |
|  | |
| template<typename T > | |
| Eigen::Matrix< T,   Eigen::Dynamic, Eigen::Dynamic > | block (const Eigen::Matrix< T, Eigen::Dynamic, Eigen::Dynamic > &m, size\_t i, size\_t j, size\_t nrows, size\_t ncols) |
|  | Return a nrows x ncols submatrix starting at (i-1,j-1). More... |
|  | |
| template<typename T > | |
| Eigen::Matrix< T,   Eigen::Dynamic, Eigen::Dynamic > | cholesky\_decompose (const Eigen::Matrix< T, Eigen::Dynamic, Eigen::Dynamic > &m) |
|  | Return the lower-triangular Cholesky factor (i.e., matrix square root) of the specified square, symmetric matrix. More... |
|  | |
| template<typename T > | |
| Eigen::Matrix< T,   Eigen::Dynamic, 1 > | col (const Eigen::Matrix< T, Eigen::Dynamic, Eigen::Dynamic > &m, size\_t j) |
|  | Return the specified column of the specified matrix using start-at-1 indexing. More... |
|  | |
| template<typename T , int R, int C> | |
| size\_t | cols (const Eigen::Matrix< T, R, C > &m) |
|  | |
| template<int R1, int C1, int R2, int C2> | |
| Eigen::Matrix< double, 1, C1 > | columns\_dot\_product (const Eigen::Matrix< double, R1, C1 > &v1, const Eigen::Matrix< double, R2, C2 > &v2) |
|  | Returns the dot product of the specified vectors. More... |
|  | |
| template<typename T , int R, int C> | |
| Eigen::Matrix< T, 1, C > | columns\_dot\_self (const Eigen::Matrix< T, R, C > &x) |
|  | Returns the dot product of each column of a matrix with itself. More... |
|  | |
| template<typename T , int R, int C> | |
| Matrix< T, Dynamic, Dynamic > | to\_matrix (Matrix< T, R, C > matrix) |
|  | |
| template<typename T > | |
| Matrix< T, Dynamic, Dynamic > | to\_matrix (const vector< vector< T > > &vec) |
|  | |
| Matrix< double, Dynamic, Dynamic > | to\_matrix (const vector< vector< int > > &vec) |
|  | |
| template<typename T , int R, int C> | |
| Matrix< T, Dynamic, 1 > | to\_vector (const Matrix< T, R, C > &matrix) |
|  | |
| template<typename T > | |
| Matrix< T, Dynamic, 1 > | to\_vector (const vector< T > &vec) |
|  | |
| Matrix< double, Dynamic, 1 > | to\_vector (const vector< int > &vec) |
|  | |
| template<typename T , int R, int C> | |
| Matrix< T, 1, Dynamic > | to\_row\_vector (const Matrix< T, R, C > &matrix) |
|  | |
| template<typename T > | |
| Matrix< T, 1, Dynamic > | to\_row\_vector (const vector< T > &vec) |
|  | |
| Matrix< double, 1, Dynamic > | to\_row\_vector (const vector< int > &vec) |
|  | |
| template<typename T > | |
| vector< vector< T > > | to\_array\_2d (const Matrix< T, Dynamic, Dynamic > &matrix) |
|  | |
| template<typename T , int R, int C> | |
| vector< T > | to\_array\_1d (const Matrix< T, R, C > &matrix) |
|  | |
| template<typename T > | |
| vector< T > | to\_array\_1d (const vector< T > &x) |
|  | |
| template<typename T > | |
| vector< typename scalar\_type  < T >::type > | to\_array\_1d (const vector< vector< T > > &x) |
|  | |
| matrix\_d | crossprod (const matrix\_d &M) |
|  | Returns the result of pre-multiplying a matrix by its own transpose. More... |
|  | |
| template<typename T > | |
| std::vector< T > | cumulative\_sum (const std::vector< T > &x) |
|  | Return the cumulative sum of the specified vector. More... |
|  | |
| template<typename T , int R, int C> | |
| Eigen::Matrix< T, R, C > | cumulative\_sum (const Eigen::Matrix< T, R, C > &m) |
|  | Return the cumulative sum of the specified matrix. More... |
|  | |
| template<typename T , int R, int C> | |
| T | determinant (const Eigen::Matrix< T, R, C > &m) |
|  | Returns the determinant of the specified square matrix. More... |
|  | |
| template<typename T > | |
| Eigen::Matrix< T,   Eigen::Dynamic, Eigen::Dynamic > | diag\_matrix (const Eigen::Matrix< T, Eigen::Dynamic, 1 > &v) |
|  | Return a square diagonal matrix with the specified vector of coefficients as the diagonal values. More... |
|  | |
| template<typename T1 , typename T2 , int R1, int C1, int R2, int C2> | |
| Eigen::Matrix< typename   boost::math::tools::promote\_args  < T1, T2 >::type, R1, C1 > | diag\_post\_multiply (const Eigen::Matrix< T1, R1, C1 > &m1, const Eigen::Matrix< T2, R2, C2 > &m2) |
|  | |
| template<typename T1 , typename T2 , int R1, int C1, int R2, int C2> | |
| Eigen::Matrix< typename   boost::math::tools::promote\_args  < T1, T2 >::type, R2, C2 > | diag\_pre\_multiply (const Eigen::Matrix< T1, R1, C1 > &m1, const Eigen::Matrix< T2, R2, C2 > &m2) |
|  | |
| template<typename T > | |
| Eigen::Matrix< T,   Eigen::Dynamic, 1 > | diagonal (const Eigen::Matrix< T, Eigen::Dynamic, Eigen::Dynamic > &m) |
|  | Return a column vector of the diagonal elements of the specified matrix. More... |
|  | |
| template<typename T > | |
| void | dims (const T &x, std::vector< int > &result) |
|  | |
| template<typename T , int R, int C> | |
| void | dims (const Eigen::Matrix< T, R, C > &x, std::vector< int > &result) |
|  | |
| template<typename T > | |
| void | dims (const std::vector< T > &x, std::vector< int > &result) |
|  | |
| template<typename T > | |
| std::vector< int > | dims (const T &x) |
|  | |
| template<typename T1 , int R1, int C1, typename T2 , int R2, int C2> | |
| boost::math::tools::promote\_args  < T1, T2 >::type | distance (const Eigen::Matrix< T1, R1, C1 > &v1, const Eigen::Matrix< T2, R2, C2 > &v2) |
|  | Returns the distance between the specified vectors. More... |
|  | |
| template<int R, int C, typename T > | |
| boost::enable\_if\_c  < boost::is\_arithmetic< T >  ::value, Eigen::Matrix< double,   R, C > >::type | divide (const Eigen::Matrix< double, R, C > &m, T c) |
|  | Return specified matrix divided by specified scalar. More... |
|  | |
| template<int R1, int C1, int R2, int C2> | |
| double | dot\_product (const Eigen::Matrix< double, R1, C1 > &v1, const Eigen::Matrix< double, R2, C2 > &v2) |
|  | Returns the dot product of the specified vectors. More... |
|  | |
| double | dot\_product (const double \*v1, const double \*v2, size\_t length) |
|  | Returns the dot product of the specified arrays of doubles. More... |
|  | |
| double | dot\_product (const std::vector< double > &v1, const std::vector< double > &v2) |
|  | Returns the dot product of the specified arrays of doubles. More... |
|  | |
| template<int R, int C> | |
| double | dot\_self (const Eigen::Matrix< double, R, C > &v) |
|  | Returns the dot product of the specified vector with itself. More... |
|  | |
| template<typename T > | |
| Eigen::Matrix< T,   Eigen::Dynamic, 1 > | eigenvalues\_sym (const Eigen::Matrix< T, Eigen::Dynamic, Eigen::Dynamic > &m) |
|  | Return the eigenvalues of the specified symmetric matrix in descending order of magnitude. More... |
|  | |
| template<typename T > | |
| Eigen::Matrix< T,   Eigen::Dynamic, Eigen::Dynamic > | eigenvectors\_sym (const Eigen::Matrix< T, Eigen::Dynamic, Eigen::Dynamic > &m) |
|  | |
| template<typename T1 , typename T2 , int R, int C> | |
| Eigen::Matrix< typename   boost::math::tools::promote\_args  < T1, T2 >::type, R, C > | elt\_divide (const Eigen::Matrix< T1, R, C > &m1, const Eigen::Matrix< T2, R, C > &m2) |
|  | Return the elementwise division of the specified matrices. More... |
|  | |
| template<typename T1 , typename T2 , int R, int C> | |
| Eigen::Matrix< typename   boost::math::tools::promote\_args  < T1, T2 >::type, R, C > | elt\_divide (const Eigen::Matrix< T1, R, C > &m, T2 s) |
|  | Return the elementwise division of the specified matrix by the specified scalar. More... |
|  | |
| template<typename T1 , typename T2 , int R, int C> | |
| Eigen::Matrix< typename   boost::math::tools::promote\_args  < T1, T2 >::type, R, C > | elt\_divide (T1 s, const Eigen::Matrix< T2, R, C > &m) |
|  | Return the elementwise division of the specified scalar by the specified matrix. More... |
|  | |
| template<typename T1 , typename T2 , int R, int C> | |
| Eigen::Matrix< typename   boost::math::tools::promote\_args  < T1, T2 >::type, R, C > | elt\_multiply (const Eigen::Matrix< T1, R, C > &m1, const Eigen::Matrix< T2, R, C > &m2) |
|  | Return the elementwise multiplication of the specified matrices. More... |
|  | |
| template<typename T , int Rows, int Cols> | |
| Eigen::Matrix< T, Rows, Cols > | exp (const Eigen::Matrix< T, Rows, Cols > &m) |
|  | Return the element-wise exponentiation of the matrix or vector. More... |
|  | |
| template<int Rows, int Cols> | |
| Eigen::Matrix< double, Rows, Cols > | exp (const Eigen::Matrix< double, Rows, Cols > &m) |
|  | |
| template<typename T , typename S > | |
| void | fill (T &x, const S &y) |
|  | Fill the specified container with the specified value. More... |
|  | |
| template<typename T , int R, int C, typename S > | |
| void | fill (Eigen::Matrix< T, R, C > &x, const S &y) |
|  | Fill the specified container with the specified value. More... |
|  | |
| template<typename T , typename S > | |
| void | fill (std::vector< T > &x, const S &y) |
|  | Fill the specified container with the specified value. More... |
|  | |
| template<typename T > | |
| const T & | get\_base1 (const std::vector< T > &x, size\_t i, const char \*error\_msg, size\_t idx) |
|  | Return a reference to the value of the specified vector at the specified base-one index. More... |
|  | |
| template<typename T > | |
| const T & | get\_base1 (const std::vector< std::vector< T > > &x, size\_t i1, size\_t i2, const char \*error\_msg, size\_t idx) |
|  | Return a reference to the value of the specified vector at the specified base-one indexes. More... |
|  | |
| template<typename T > | |
| const T & | get\_base1 (const std::vector< std::vector< std::vector< T > > > &x, size\_t i1, size\_t i2, size\_t i3, const char \*error\_msg, size\_t idx) |
|  | Return a reference to the value of the specified vector at the specified base-one indexes. More... |
|  | |
| template<typename T > | |
| const T & | get\_base1 (const std::vector< std::vector< std::vector< std::vector< T > > > > &x, size\_t i1, size\_t i2, size\_t i3, size\_t i4, const char \*error\_msg, size\_t idx) |
|  | Return a reference to the value of the specified vector at the specified base-one indexes. More... |
|  | |
| template<typename T > | |
| const T & | get\_base1 (const std::vector< std::vector< std::vector< std::vector< std::vector< T > > > > > &x, size\_t i1, size\_t i2, size\_t i3, size\_t i4, size\_t i5, const char \*error\_msg, size\_t idx) |
|  | Return a reference to the value of the specified vector at the specified base-one indexes. More... |
|  | |
| template<typename T > | |
| const T & | get\_base1 (const std::vector< std::vector< std::vector< std::vector< std::vector< std::vector< T > > > > > > &x, size\_t i1, size\_t i2, size\_t i3, size\_t i4, size\_t i5, size\_t i6, const char \*error\_msg, size\_t idx) |
|  | Return a reference to the value of the specified vector at the specified base-one indexes. More... |
|  | |
| template<typename T > | |
| const T & | get\_base1 (const std::vector< std::vector< std::vector< std::vector< std::vector< std::vector< std::vector< T > > > > > > > &x, size\_t i1, size\_t i2, size\_t i3, size\_t i4, size\_t i5, size\_t i6, size\_t i7, const char \*error\_msg, size\_t idx) |
|  | Return a reference to the value of the specified vector at the specified base-one indexes. More... |
|  | |
| template<typename T > | |
| const T & | get\_base1 (const std::vector< std::vector< std::vector< std::vector< std::vector< std::vector< std::vector< std::vector< T > > > > > > > > &x, size\_t i1, size\_t i2, size\_t i3, size\_t i4, size\_t i5, size\_t i6, size\_t i7, size\_t i8, const char \*error\_msg, size\_t idx) |
|  | Return a reference to the value of the specified vector at the specified base-one indexes. More... |
|  | |
| template<typename T > | |
| Eigen::Matrix< T,   1, Eigen::Dynamic > | get\_base1 (const Eigen::Matrix< T, Eigen::Dynamic, Eigen::Dynamic > &x, size\_t m, const char \*error\_msg, size\_t idx) |
|  | Return a copy of the row of the specified vector at the specified base-one row index. More... |
|  | |
| template<typename T > | |
| const T & | get\_base1 (const Eigen::Matrix< T, Eigen::Dynamic, Eigen::Dynamic > &x, size\_t m, size\_t n, const char \*error\_msg, size\_t idx) |
|  | Return a reference to the value of the specified matrix at the specified base-one row and column indexes. More... |
|  | |
| template<typename T > | |
| const T & | get\_base1 (const Eigen::Matrix< T, Eigen::Dynamic, 1 > &x, size\_t m, const char \*error\_msg, size\_t idx) |
|  | Return a reference to the value of the specified column vector at the specified base-one index. More... |
|  | |
| template<typename T > | |
| const T & | get\_base1 (const Eigen::Matrix< T, 1, Eigen::Dynamic > &x, size\_t n, const char \*error\_msg, size\_t idx) |
|  | Return a reference to the value of the specified row vector at the specified base-one index. More... |
|  | |
| template<typename T > | |
| T & | get\_base1\_lhs (std::vector< T > &x, size\_t i, const char \*error\_msg, size\_t idx) |
|  | Return a reference to the value of the specified vector at the specified base-one index. More... |
|  | |
| template<typename T > | |
| T & | get\_base1\_lhs (std::vector< std::vector< T > > &x, size\_t i1, size\_t i2, const char \*error\_msg, size\_t idx) |
|  | Return a reference to the value of the specified vector at the specified base-one indexes. More... |
|  | |
| template<typename T > | |
| T & | get\_base1\_lhs (std::vector< std::vector< std::vector< T > > > &x, size\_t i1, size\_t i2, size\_t i3, const char \*error\_msg, size\_t idx) |
|  | Return a reference to the value of the specified vector at the specified base-one indexes. More... |
|  | |
| template<typename T > | |
| T & | get\_base1\_lhs (std::vector< std::vector< std::vector< std::vector< T > > > > &x, size\_t i1, size\_t i2, size\_t i3, size\_t i4, const char \*error\_msg, size\_t idx) |
|  | Return a reference to the value of the specified vector at the specified base-one indexes. More... |
|  | |
| template<typename T > | |
| T & | get\_base1\_lhs (std::vector< std::vector< std::vector< std::vector< std::vector< T > > > > > &x, size\_t i1, size\_t i2, size\_t i3, size\_t i4, size\_t i5, const char \*error\_msg, size\_t idx) |
|  | Return a reference to the value of the specified vector at the specified base-one indexes. More... |
|  | |
| template<typename T > | |
| T & | get\_base1\_lhs (std::vector< std::vector< std::vector< std::vector< std::vector< std::vector< T > > > > > > &x, size\_t i1, size\_t i2, size\_t i3, size\_t i4, size\_t i5, size\_t i6, const char \*error\_msg, size\_t idx) |
|  | Return a reference to the value of the specified vector at the specified base-one indexes. More... |
|  | |
| template<typename T > | |
| T & | get\_base1\_lhs (std::vector< std::vector< std::vector< std::vector< std::vector< std::vector< std::vector< T > > > > > > > &x, size\_t i1, size\_t i2, size\_t i3, size\_t i4, size\_t i5, size\_t i6, size\_t i7, const char \*error\_msg, size\_t idx) |
|  | Return a reference to the value of the specified vector at the specified base-one indexes. More... |
|  | |
| template<typename T > | |
| T & | get\_base1\_lhs (std::vector< std::vector< std::vector< std::vector< std::vector< std::vector< std::vector< std::vector< T > > > > > > > > &x, size\_t i1, size\_t i2, size\_t i3, size\_t i4, size\_t i5, size\_t i6, size\_t i7, size\_t i8, const char \*error\_msg, size\_t idx) |
|  | Return a reference to the value of the specified vector at the specified base-one indexes. More... |
|  | |
| template<typename T > | |
| Eigen::Block< Eigen::Matrix< T,   Eigen::Dynamic, Eigen::Dynamic > > | get\_base1\_lhs (Eigen::Matrix< T, Eigen::Dynamic, Eigen::Dynamic > &x, size\_t m, const char \*error\_msg, size\_t idx) |
|  | Return a copy of the row of the specified vector at the specified base-one row index. More... |
|  | |
| template<typename T > | |
| T & | get\_base1\_lhs (Eigen::Matrix< T, Eigen::Dynamic, Eigen::Dynamic > &x, size\_t m, size\_t n, const char \*error\_msg, size\_t idx) |
|  | Return a reference to the value of the specified matrix at the specified base-one row and column indexes. More... |
|  | |
| template<typename T > | |
| T & | get\_base1\_lhs (Eigen::Matrix< T, Eigen::Dynamic, 1 > &x, size\_t m, const char \*error\_msg, size\_t idx) |
|  | Return a reference to the value of the specified column vector at the specified base-one index. More... |
|  | |
| template<typename T > | |
| T & | get\_base1\_lhs (Eigen::Matrix< T, 1, Eigen::Dynamic > &x, size\_t n, const char \*error\_msg, size\_t idx) |
|  | Return a reference to the value of the specified row vector at the specified base-one index. More... |
|  | |
| template<typename T\_lp , typename T\_lp\_accum > | |
| boost::math::tools::promote\_args  < T\_lp, T\_lp\_accum >::type | get\_lp (const T\_lp &lp, const stan::math::accumulator< T\_lp\_accum > &lp\_accum) |
|  | |
| template<typename T > | |
| Eigen::Matrix< T,   Eigen::Dynamic, 1 > | head (const Eigen::Matrix< T, Eigen::Dynamic, 1 > &v, size\_t n) |
|  | Return the specified number of elements as a vector from the front of the specified vector. More... |
|  | |
| template<typename T > | |
| Eigen::Matrix< T,   1, Eigen::Dynamic > | head (const Eigen::Matrix< T, 1, Eigen::Dynamic > &rv, size\_t n) |
|  | Return the specified number of elements as a row vector from the front of the specified row vector. More... |
|  | |
| template<typename T > | |
| std::vector< T > | head (const std::vector< T > &sv, size\_t n) |
|  | Return the specified number of elements as a standard vector from the front of the specified standard vector. More... |
|  | |
| template<typename T > | |
| void | initialize (T &x, const T &v) |
|  | |
| template<typename T , typename V > | |
| boost::enable\_if\_c  < boost::is\_arithmetic< V >  ::value, void >::type | initialize (T &x, V v) |
|  | |
| template<typename T , int R, int C, typename V > | |
| void | initialize (Eigen::Matrix< T, R, C > &x, const V &v) |
|  | |
| template<typename T , typename V > | |
| void | initialize (std::vector< T > &x, const V &v) |
|  | |
| template<typename T > | |
| Eigen::Matrix< T,   Eigen::Dynamic, Eigen::Dynamic > | inverse (const Eigen::Matrix< T, Eigen::Dynamic, Eigen::Dynamic > &m) |
|  | Returns the inverse of the specified matrix. More... |
|  | |
| template<typename T > | |
| Eigen::Matrix< T,   Eigen::Dynamic, Eigen::Dynamic > | inverse\_spd (const Eigen::Matrix< T, Eigen::Dynamic, Eigen::Dynamic > &m) |
|  | Returns the inverse of the specified symmetric, pos/neg-definite matrix. More... |
|  | |
| template<typename T , int Rows, int Cols> | |
| Eigen::Matrix< T, Rows, Cols > | log (const Eigen::Matrix< T, Rows, Cols > &m) |
|  | Return the element-wise logarithm of the matrix or vector. More... |
|  | |
| template<typename T , int R, int C> | |
| T | log\_determinant (const Eigen::Matrix< T, R, C > &m) |
|  | Returns the log absolute determinant of the specified square matrix. More... |
|  | |
| template<int R, int C, typename T > | |
| T | log\_determinant\_ldlt (stan::math::LDLT\_factor< T, R, C > &A) |
|  | |
| template<typename T , int R, int C> | |
| T | log\_determinant\_spd (const Eigen::Matrix< T, R, C > &m) |
|  | Returns the log absolute determinant of the specified square matrix. More... |
|  | |
| template<typename T > | |
| Eigen::Matrix< T,   Eigen::Dynamic, 1 > | log\_softmax (const Eigen::Matrix< T, Eigen::Dynamic, 1 > &v) |
|  | Return the natural logarithm of the softmax of the specified vector. More... |
|  | |
| template<int R, int C> | |
| double | log\_sum\_exp (const Eigen::Matrix< double, R, C > &x) |
|  | Return the log of the sum of the exponentiated values of the specified matrix of values. More... |
|  | |
| int | max (const std::vector< int > &x) |
|  | Returns the maximum coefficient in the specified column vector. More... |
|  | |
| template<typename T > | |
| T | max (const std::vector< T > &x) |
|  | Returns the maximum coefficient in the specified column vector. More... |
|  | |
| template<typename T , int R, int C> | |
| T | max (const Eigen::Matrix< T, R, C > &m) |
|  | Returns the maximum coefficient in the specified vector, row vector, or matrix. More... |
|  | |
| template<typename T1 , typename T2 , int R1, int C1, int R2, int C2> | |
| Eigen::Matrix< typename   boost::math::tools::promote\_args  < T1, T2 >::type, R1, C2 > | mdivide\_left (const Eigen::Matrix< T1, R1, C1 > &A, const Eigen::Matrix< T2, R2, C2 > &b) |
|  | Returns the solution of the system Ax=b. More... |
|  | |
| template<int R1, int C1, int R2, int C2, typename T1 , typename T2 > | |
| Eigen::Matrix< typename   boost::math::tools::promote\_args  < T1, T2 >::type, R1, C2 > | mdivide\_left\_ldlt (const stan::math::LDLT\_factor< T1, R1, C1 > &A, const Eigen::Matrix< T2, R2, C2 > &b) |
|  | Returns the solution of the system Ax=b given an LDLT\_factor of A. More... |
|  | |
| template<typename T1 , typename T2 , int R1, int C1, int R2, int C2> | |
| Eigen::Matrix< typename   boost::math::tools::promote\_args  < T1, T2 >::type, R1, C2 > | mdivide\_left\_spd (const Eigen::Matrix< T1, R1, C1 > &A, const Eigen::Matrix< T2, R2, C2 > &b) |
|  | Returns the solution of the system Ax=b where A is symmetric positive definite. More... |
|  | |
| template<int TriView, typename T1 , typename T2 , int R1, int C1, int R2, int C2> | |
| Eigen::Matrix< typename   boost::math::tools::promote\_args  < T1, T2 >::type, R1, C2 > | mdivide\_left\_tri (const Eigen::Matrix< T1, R1, C1 > &A, const Eigen::Matrix< T2, R2, C2 > &b) |
|  | Returns the solution of the system Ax=b when A is triangular. More... |
|  | |
| template<int TriView, typename T , int R1, int C1> | |
| Eigen::Matrix< T, R1, C1 > | mdivide\_left\_tri (const Eigen::Matrix< T, R1, C1 > &A) |
|  | Returns the solution of the system Ax=b when A is triangular and b=I. More... |
|  | |
| template<typename T1 , typename T2 , int R1, int C1, int R2, int C2> | |
| Eigen::Matrix< typename   boost::math::tools::promote\_args  < T1, T2 >::type, R1, C2 > | mdivide\_left\_tri\_low (const Eigen::Matrix< T1, R1, C1 > &A, const Eigen::Matrix< T2, R2, C2 > &b) |
|  | |
| template<typename T , int R1, int C1> | |
| Eigen::Matrix< T, R1, C1 > | mdivide\_left\_tri\_low (const Eigen::Matrix< T, R1, C1 > &A) |
|  | |
| template<typename T1 , typename T2 , int R1, int C1, int R2, int C2> | |
| Eigen::Matrix< typename   boost::math::tools::promote\_args  < T1, T2 >::type, R1, C2 > | mdivide\_right (const Eigen::Matrix< T1, R1, C1 > &b, const Eigen::Matrix< T2, R2, C2 > &A) |
|  | Returns the solution of the system Ax=b. More... |
|  | |
| template<typename T1 , typename T2 , int R1, int C1, int R2, int C2> | |
| Eigen::Matrix< typename   boost::math::tools::promote\_args  < T1, T2 >::type, R1, C2 > | mdivide\_right\_ldlt (const Eigen::Matrix< T1, R1, C1 > &b, const stan::math::LDLT\_factor< T2, R2, C2 > &A) |
|  | Returns the solution of the system xA=b given an LDLT\_factor of A. More... |
|  | |
| template<int R1, int C1, int R2, int C2> | |
| Eigen::Matrix< double, R1, C2 > | mdivide\_right\_ldlt (const Eigen::Matrix< double, R1, C1 > &b, const stan::math::LDLT\_factor< double, R2, C2 > &A) |
|  | |
| template<typename T1 , typename T2 , int R1, int C1, int R2, int C2> | |
| Eigen::Matrix< typename   boost::math::tools::promote\_args  < T1, T2 >::type, R1, C2 > | mdivide\_right\_spd (const Eigen::Matrix< T1, R1, C1 > &b, const Eigen::Matrix< T2, R2, C2 > &A) |
|  | Returns the solution of the system Ax=b where A is symmetric positive definite. More... |
|  | |
| template<int TriView, typename T1 , typename T2 , int R1, int C1, int R2, int C2> | |
| Eigen::Matrix< typename   boost::math::tools::promote\_args  < T1, T2 >::type, R1, C2 > | mdivide\_right\_tri (const Eigen::Matrix< T1, R1, C1 > &b, const Eigen::Matrix< T2, R2, C2 > &A) |
|  | Returns the solution of the system Ax=b when A is triangular. More... |
|  | |
| template<typename T1 , typename T2 , int R1, int C1, int R2, int C2> | |
| Eigen::Matrix< typename   boost::math::tools::promote\_args  < T1, T2 >::type, R1, C2 > | mdivide\_right\_tri\_low (const Eigen::Matrix< T1, R1, C1 > &b, const Eigen::Matrix< T2, R2, C2 > &A) |
|  | Returns the solution of the system tri(A)x=b when tri(A) is a lower triangular view of the matrix A. More... |
|  | |
| template<typename T > | |
| boost::math::tools::promote\_args  < T >::type | mean (const std::vector< T > &v) |
|  | Returns the sample mean (i.e., average) of the coefficients in the specified standard vector. More... |
|  | |
| template<typename T , int R, int C> | |
| boost::math::tools::promote\_args  < T >::type | mean (const Eigen::Matrix< T, R, C > &m) |
|  | Returns the sample mean (i.e., average) of the coefficients in the specified vector, row vector, or matrix. More... |
|  | |
| int | min (const std::vector< int > &x) |
|  | Returns the minimum coefficient in the specified column vector. More... |
|  | |
| template<typename T > | |
| T | min (const std::vector< T > &x) |
|  | Returns the minimum coefficient in the specified column vector. More... |
|  | |
| template<typename T , int R, int C> | |
| T | min (const Eigen::Matrix< T, R, C > &m) |
|  | Returns the minimum coefficient in the specified matrix, vector, or row vector. More... |
|  | |
| template<typename T > | |
| T | minus (const T &x) |
|  | Returns the negation of the specified scalar or matrix. More... |
|  | |
| template<int R, int C, typename T > | |
| boost::enable\_if\_c  < boost::is\_arithmetic< T >  ::value, Eigen::Matrix< double,   R, C > >::type | multiply (const Eigen::Matrix< double, R, C > &m, T c) |
|  | Return specified matrix multiplied by specified scalar. More... |
|  | |
| template<int R, int C, typename T > | |
| boost::enable\_if\_c  < boost::is\_arithmetic< T >  ::value, Eigen::Matrix< double,   R, C > >::type | multiply (T c, const Eigen::Matrix< double, R, C > &m) |
|  | Return specified scalar multiplied by specified matrix. More... |
|  | |
| template<int R1, int C1, int R2, int C2> | |
| Eigen::Matrix< double, R1, C2 > | multiply (const Eigen::Matrix< double, R1, C1 > &m1, const Eigen::Matrix< double, R2, C2 > &m2) |
|  | Return the product of the specified matrices. More... |
|  | |
| template<int C1, int R2> | |
| double | multiply (const Eigen::Matrix< double, 1, C1 > &rv, const Eigen::Matrix< double, R2, 1 > &v) |
|  | Return the scalar product of the specified row vector and specified column vector. More... |
|  | |
| matrix\_d | multiply\_lower\_tri\_self\_transpose (const matrix\_d &L) |
|  | Returns the result of multiplying the lower triangular portion of the input matrix by its own transpose. More... |
|  | |
| template<typename T > | |
| int | num\_elements (const T &x) |
|  | Returns 1, the number of elements in a primitive type. More... |
|  | |
| template<typename T , int R, int C> | |
| int | num\_elements (const Eigen::Matrix< T, R, C > &m) |
|  | Returns the size of the specified matrix. More... |
|  | |
| template<typename T > | |
| int | num\_elements (const std::vector< T > &v) |
|  | Returns the number of elements in the specified vector. More... |
|  | |
| template<typename T > | |
| T | prod (const std::vector< T > &v) |
|  | Returns the product of the coefficients of the specified standard vector. More... |
|  | |
| template<typename T , int R, int C> | |
| T | prod (const Eigen::Matrix< T, R, C > &v) |
|  | Returns the product of the coefficients of the specified column vector. More... |
|  | |
| template<typename T1 , typename T2 , typename F > | |
| common\_type< T1, T2 >::type | promote\_common (const F &u) |
|  | |
| template<typename T > | |
| Eigen::Matrix< T,   Eigen::Dynamic, Eigen::Dynamic > | qr\_Q (const Eigen::Matrix< T, Eigen::Dynamic, Eigen::Dynamic > &m) |
|  | |
| template<typename T > | |
| Eigen::Matrix< T,   Eigen::Dynamic, Eigen::Dynamic > | qr\_R (const Eigen::Matrix< T, Eigen::Dynamic, Eigen::Dynamic > &m) |
|  | |
| template<int RA, int CA, int RB, int CB, typename T > | |
| Eigen::Matrix< T, CB, CB > | quad\_form (const Eigen::Matrix< T, RA, CA > &A, const Eigen::Matrix< T, RB, CB > &B) |
|  | Compute B^T A B. More... |
|  | |
| template<int RA, int CA, int RB, typename T > | |
| T | quad\_form (const Eigen::Matrix< T, RA, CA > &A, const Eigen::Matrix< T, RB, 1 > &B) |
|  | |
| template<int RA, int CA, int RB, int CB, typename T > | |
| Eigen::Matrix< T, CB, CB > | quad\_form\_sym (const Eigen::Matrix< T, RA, CA > &A, const Eigen::Matrix< T, RB, CB > &B) |
|  | |
| template<int RA, int CA, int RB, typename T > | |
| T | quad\_form\_sym (const Eigen::Matrix< T, RA, CA > &A, const Eigen::Matrix< T, RB, 1 > &B) |
|  | |
| template<typename T1 , typename T2 , int R, int C> | |
| Matrix< typename promote\_args  < T1, T2 >::type, Dynamic,   Dynamic > | quad\_form\_diag (const Matrix< T1, Dynamic, Dynamic > &mat, const Matrix< T2, R, C > &vec) |
|  | |
| template<typename T > | |
| size\_t | rank (const std::vector< T > &v, int s) |
|  | Return the number of components of v less than v[s]. More... |
|  | |
| template<typename T , int R, int C> | |
| size\_t | rank (const Eigen::Matrix< T, R, C > &v, int s) |
|  | Return the number of components of v less than v[s]. More... |
|  | |
| template<typename T > | |
| void | resize (T &x, std::vector< size\_t > dims) |
|  | Recursively resize the specified vector of vectors, which must bottom out at scalar values, Eigen vectors or Eigen matrices. More... |
|  | |
| template<typename T > | |
| Eigen::Matrix< T,   1, Eigen::Dynamic > | row (const Eigen::Matrix< T, Eigen::Dynamic, Eigen::Dynamic > &m, size\_t i) |
|  | Return the specified row of the specified matrix, using start-at-1 indexing. More... |
|  | |
| template<typename T , int R, int C> | |
| size\_t | rows (const Eigen::Matrix< T, R, C > &m) |
|  | |
| template<int R1, int C1, int R2, int C2> | |
| Eigen::Matrix< double, R1, 1 > | rows\_dot\_product (const Eigen::Matrix< double, R1, C1 > &v1, const Eigen::Matrix< double, R2, C2 > &v2) |
|  | Returns the dot product of the specified vectors. More... |
|  | |
| template<typename T , int R, int C> | |
| Eigen::Matrix< T, R, 1 > | rows\_dot\_self (const Eigen::Matrix< T, R, C > &x) |
|  | Returns the dot product of each row of a matrix with itself. More... |
|  | |
| template<typename T > | |
| boost::math::tools::promote\_args  < T >::type | sd (const std::vector< T > &v) |
|  | Returns the unbiased sample standard deviation of the coefficients in the specified column vector. More... |
|  | |
| template<typename T , int R, int C> | |
| boost::math::tools::promote\_args  < T >::type | sd (const Eigen::Matrix< T, R, C > &m) |
|  | Returns the unbiased sample standard deviation of the coefficients in the specified vector, row vector, or matrix. More... |
|  | |
| template<typename T > | |
| Eigen::Matrix< T,   Eigen::Dynamic, 1 > | segment (const Eigen::Matrix< T, Eigen::Dynamic, 1 > &v, size\_t i, size\_t n) |
|  | Return the specified number of elements as a vector starting from the specified element - 1 of the specified vector. More... |
|  | |
| template<typename T > | |
| Eigen::Matrix< T,   1, Eigen::Dynamic > | segment (const Eigen::Matrix< T, 1, Eigen::Dynamic > &v, size\_t i, size\_t n) |
|  | |
| template<typename T > | |
| std::vector< T > | segment (const std::vector< T > &sv, size\_t i, size\_t n) |
|  | |
| template<typename T > | |
| Eigen::Matrix< T,   Eigen::Dynamic, 1 > | singular\_values (const Eigen::Matrix< T, Eigen::Dynamic, Eigen::Dynamic > &m) |
|  | Return the vector of the singular values of the specified matrix in decreasing order of magnitude. More... |
|  | |
| template<typename T > | |
| int | size (const std::vector< T > &x) |
|  | |
| template<typename T > | |
| Eigen::Matrix< T,   Eigen::Dynamic, 1 > | softmax (const Eigen::Matrix< T, Eigen::Dynamic, 1 > &v) |
|  | Return the softmax of the specified vector. More... |
|  | |
| template<typename T > | |
| std::vector< T > | sort\_asc (std::vector< T > xs) |
|  | Return the specified standard vector in ascending order. More... |
|  | |
| template<typename T > | |
| std::vector< T > | sort\_desc (std::vector< T > xs) |
|  | Return the specified standard vector in descending order. More... |
|  | |
| template<typename T , int R, int C> | |
| Eigen::Matrix< T, R, C > | sort\_asc (Eigen::Matrix< T, R, C > xs) |
|  | Return the specified eigen vector in ascending order. More... |
|  | |
| template<typename T , int R, int C> | |
| Eigen::Matrix< T, R, C > | sort\_desc (Eigen::Matrix< T, R, C > xs) |
|  | Return the specified eigen vector in descending order. More... |
|  | |
| template<typename C > | |
| std::vector< int > | sort\_indices\_asc (const C &xs) |
|  | Return a sorted copy of the argument container in ascending order. More... |
|  | |
| template<typename C > | |
| std::vector< int > | sort\_indices\_desc (const C &xs) |
|  | Return a sorted copy of the argument container in ascending order. More... |
|  | |
| template<int R1, int C1, int R2, int C2, typename T1 , typename T2 > | |
| boost::math::tools::promote\_args  < T1, T2 >::type | squared\_distance (const Eigen::Matrix< T1, R1, C1 > &v1, const Eigen::Matrix< T2, R2, C2 > &v2) |
|  | Returns the squared distance between the specified vectors. More... |
|  | |
| template<typename T > | |
| void | stan\_print (std::ostream \*o, const T &x) |
|  | |
| template<typename T > | |
| void | stan\_print (std::ostream \*o, const std::vector< T > &x) |
|  | |
| template<typename T > | |
| void | stan\_print (std::ostream \*o, const Eigen::Matrix< T, Eigen::Dynamic, 1 > &x) |
|  | |
| template<typename T > | |
| void | stan\_print (std::ostream \*o, const Eigen::Matrix< T, 1, Eigen::Dynamic > &x) |
|  | |
| template<typename T > | |
| void | stan\_print (std::ostream \*o, const Eigen::Matrix< T, Eigen::Dynamic, Eigen::Dynamic > &x) |
|  | |
| template<typename T > | |
| Eigen::Matrix< T,   Eigen::Dynamic, 1 > | sub\_col (const Eigen::Matrix< T, Eigen::Dynamic, Eigen::Dynamic > &m, size\_t i, size\_t j, size\_t nrows) |
|  | Return a nrows x 1 subcolumn starting at (i-1,j-1). More... |
|  | |
| template<typename T > | |
| Eigen::Matrix< T,   1, Eigen::Dynamic > | sub\_row (const Eigen::Matrix< T, Eigen::Dynamic, Eigen::Dynamic > &m, size\_t i, size\_t j, size\_t ncols) |
|  | Return a 1 x nrows subrow starting at (i-1,j-1). More... |
|  | |
| template<typename T1 , typename T2 , int R, int C> | |
| Eigen::Matrix< typename   boost::math::tools::promote\_args  < T1, T2 >::type, R, C > | subtract (const Eigen::Matrix< T1, R, C > &m1, const Eigen::Matrix< T2, R, C > &m2) |
|  | Return the result of subtracting the second specified matrix from the first specified matrix. More... |
|  | |
| template<typename T1 , typename T2 , int R, int C> | |
| Eigen::Matrix< typename   boost::math::tools::promote\_args  < T1, T2 >::type, R, C > | subtract (const T1 &c, const Eigen::Matrix< T2, R, C > &m) |
|  | |
| template<typename T1 , typename T2 , int R, int C> | |
| Eigen::Matrix< typename   boost::math::tools::promote\_args  < T1, T2 >::type, R, C > | subtract (const Eigen::Matrix< T1, R, C > &m, const T2 &c) |
|  | |
| template<typename T > | |
| T | sum (const std::vector< T > &xs) |
|  | Return the sum of the values in the specified standard vector. More... |
|  | |
| template<typename T , int R, int C> | |
| double | sum (const Eigen::Matrix< T, R, C > &v) |
|  | Returns the sum of the coefficients of the specified column vector. More... |
|  | |
| template<typename T > | |
| Eigen::Matrix< T,   Eigen::Dynamic, 1 > | tail (const Eigen::Matrix< T, Eigen::Dynamic, 1 > &v, size\_t n) |
|  | Return the specified number of elements as a vector from the back of the specified vector. More... |
|  | |
| template<typename T > | |
| Eigen::Matrix< T,   1, Eigen::Dynamic > | tail (const Eigen::Matrix< T, 1, Eigen::Dynamic > &rv, size\_t n) |
|  | Return the specified number of elements as a row vector from the back of the specified row vector. More... |
|  | |
| template<typename T > | |
| std::vector< T > | tail (const std::vector< T > &sv, size\_t n) |
|  | |
| matrix\_d | tcrossprod (const matrix\_d &M) |
|  | Returns the result of post-multiplying a matrix by its own transpose. More... |
|  | |
| template<typename T > | |
| T | trace (const Eigen::Matrix< T, Eigen::Dynamic, Eigen::Dynamic > &m) |
|  | Returns the trace of the specified matrix. More... |
|  | |
| template<typename T > | |
| T | trace (const T &m) |
|  | |
| template<typename T1 , typename T2 , typename T3 , int R1, int C1, int R2, int C2, int R3, int C3> | |
| boost::enable\_if\_c  <!stan::is\_var< T1 >::value   &&!stan::is\_var< T2 >::value   &&!stan::is\_var< T3 >::value,   typename   boost::math::tools::promote\_args  < T1, T2, T3 >::type >::type | trace\_gen\_inv\_quad\_form\_ldlt (const Eigen::Matrix< T1, R1, C1 > &D, const stan::math::LDLT\_factor< T2, R2, C2 > &A, const Eigen::Matrix< T3, R3, C3 > &B) |
|  | |
| template<int RD, int CD, int RA, int CA, int RB, int CB> | |
| double | trace\_gen\_quad\_form (const Eigen::Matrix< double, RD, CD > &D, const Eigen::Matrix< double, RA, CA > &A, const Eigen::Matrix< double, RB, CB > &B) |
|  | Compute trace(D B^T A B). More... |
|  | |
| template<typename T1 , typename T2 , int R2, int C2, int R3, int C3> | |
| boost::enable\_if\_c  <!stan::is\_var< T1 >::value   &&!stan::is\_var< T2 >::value,   typename   boost::math::tools::promote\_args  < T1, T2 >::type >::type | trace\_inv\_quad\_form\_ldlt (const stan::math::LDLT\_factor< T1, R2, C2 > &A, const Eigen::Matrix< T2, R3, C3 > &B) |
|  | |
| template<int RA, int CA, int RB, int CB> | |
| double | trace\_quad\_form (const Eigen::Matrix< double, RA, CA > &A, const Eigen::Matrix< double, RB, CB > &B) |
|  | Compute trace(B^T A B). More... |
|  | |
| template<typename T , int R, int C> | |
| Eigen::Matrix< T, C, R > | transpose (const Eigen::Matrix< T, R, C > &m) |
|  | |
| template<typename T > | |
| boost::math::tools::promote\_args  < T >::type | variance (const std::vector< T > &v) |
|  | Returns the sample variance (divide by length - 1) of the coefficients in the specified standard vector. More... |
|  | |
| template<typename T , int R, int C> | |
| boost::math::tools::promote\_args  < T >::type | variance (const Eigen::Matrix< T, R, C > &m) |
|  | Returns the sample variance (divide by length - 1) of the coefficients in the specified column vector. More... |
|  | |
| template<typename F , typename T1 , typename T2 > | |
| std::vector< std::vector  < typename stan::return\_type  < T1, T2 >::type > > | integrate\_ode (const F &f, const std::vector< T1 > y0, const double t0, const std::vector< double > &ts, const std::vector< T2 > &theta, const std::vector< double > &x, const std::vector< int > &x\_int, std::ostream \*msgs) |
|  | Return the solutions for the specified system of ordinary differential equations given the specified initial state, initial times, times of desired solution, and parameters and data, writing error and warning messages to the specified stream. More... |
|  | |
| template<typename T > | |
| std::vector< T > | rep\_array (const T &x, int n) |
|  | |
| template<typename T > | |
| std::vector< std::vector< T > > | rep\_array (const T &x, int m, int n) |
|  | |
| template<typename T > | |
| std::vector< std::vector  < std::vector< T > > > | rep\_array (const T &x, int k, int m, int n) |
|  | |
| template<typename T > | |
| Eigen::Matrix< typename   boost::math::tools::promote\_args  < T >::type, Eigen::Dynamic,   Eigen::Dynamic > | rep\_matrix (const T &x, int m, int n) |
|  | |
| template<typename T > | |
| Eigen::Matrix< T,   Eigen::Dynamic, Eigen::Dynamic > | rep\_matrix (const Eigen::Matrix< T, Eigen::Dynamic, 1 > &v, int n) |
|  | |
| template<typename T > | |
| Eigen::Matrix< T,   Eigen::Dynamic, Eigen::Dynamic > | rep\_matrix (const Eigen::Matrix< T, 1, Eigen::Dynamic > &rv, int m) |
|  | |
| template<typename T > | |
| Eigen::Matrix< typename   boost::math::tools::promote\_args  < T >::type, 1, Eigen::Dynamic > | rep\_row\_vector (const T &x, int m) |
|  | |
| template<typename T > | |
| Eigen::Matrix< typename   boost::math::tools::promote\_args  < T >::type, Eigen::Dynamic, 1 > | rep\_vector (const T &x, int n) |
|  | |
| double | F32 (double a, double b, double c, double d, double e, double z, double precision=1e-6) |
|  | |
| void | gradF32 (double \*g, double a, double b, double c, double d, double e, double z, double precision=1e-6) |
|  | |
| void | grad2F1 (double &gradA, double &gradC, double a, double b, double c, double z, double precision=1e-6) |
|  | |
| void | gradIncBeta (double &g1, double &g2, double a, double b, double z) |
|  | |
| void | gradRegIncBeta (double &g1, double &g2, double a, double b, double z, double digammaA, double digammaB, double digammaSum, double betaAB) |
|  | |
| double | gradRegIncGamma (double a, double z, double g, double dig, double precision=1e-6) |
|  | |

|  |  |
| --- | --- |
| Variables | |
| const double | E = boost::math::constants::e<double>() |
|  | The base of the natural logarithm, . More... |
|  | |
| const double | SQRT\_2 = std::sqrt(2.0) |
|  | The value of the square root of 2, . More... |
|  | |
| const double | INV\_SQRT\_2 = 1.0 / SQRT\_2 |
|  | The value of 1 over the square root of 2, . More... |
|  | |
| const double | LOG\_2 = std::log(2.0) |
|  | The natural logarithm of 2, . More... |
|  | |
| const double | LOG\_10 = std::log(10.0) |
|  | The natural logarithm of 10, . More... |
|  | |
| const double | INFTY = std::numeric\_limits<double>::infinity() |
|  | Positive infinity. More... |
|  | |
| const double | NEGATIVE\_INFTY = - std::numeric\_limits<double>::infinity() |
|  | Negative infinity. More... |
|  | |
| const double | NOT\_A\_NUMBER = std::numeric\_limits<double>::quiet\_NaN() |
|  | (Quiet) not-a-number value. More... |
|  | |
| const double | EPSILON = std::numeric\_limits<double>::epsilon() |
|  | Smallest positive value. More... |
|  | |
| const double | NEGATIVE\_EPSILON = - std::numeric\_limits<double>::epsilon() |
|  | Largest negative value (i.e., smallest absolute value). More... |
|  | |
| const double | LOG\_PI\_OVER\_FOUR = std::log(boost::math::constants::pi<double>()) / 4.0 |
|  | |
| const double | TWO\_OVER\_SQRT\_PI = 2.0 / std::sqrt(boost::math::constants::pi<double>()) |
|  | |
| const double | NEG\_TWO\_OVER\_SQRT\_PI = -TWO\_OVER\_SQRT\_PI |
|  | |
| const double | INV\_SQRT\_TWO\_PI = 1.0 / std::sqrt(2.0 \* boost::math::constants::pi<double>()) |
|  | |
| const double | CONSTRAINT\_TOLERANCE = 1E-8 |
|  | The tolerance for checking arithmetic bounds In rank and in simplexes. More... |
|  | |

## Detailed Description

Matrices and templated mathematical functions.

## Typedef Documentation

typedef Eigen::Matrix<double,Eigen::Dynamic,Eigen::Dynamic> stan::math::matrix\_d

Type for matrix of double values.

Definition at line 23 of file typedefs.hpp.

typedef Eigen::Matrix<double,1,Eigen::Dynamic> stan::math::row\_vector\_d

Type for (row) vector of double values.

Definition at line 37 of file typedefs.hpp.

typedef index\_type<Eigen::Matrix<double,Eigen::Dynamic,Eigen::Dynamic> >::type stan::math::size\_type

Type for sizes and indexes in an Eigen matrix with double e.

Definition at line 16 of file typedefs.hpp.

typedef Eigen::Matrix<double,Eigen::Dynamic,1> stan::math::vector\_d

Type for (column) vector of double values.

Definition at line 30 of file typedefs.hpp.

## Function Documentation

|  |  |  |  |  |  |
| --- | --- | --- | --- | --- | --- |
| double stan::math::abs | ( | double | *x* | ) |  |

Return floating-point absolute value.

Delegates to `fabs(double)` rather than `std::abs(int)`.

Parameters
:   |  |  |
    | --- | --- |
    | x | scalar |

Returns
:   absolute value of scalar

Definition at line 19 of file abs.hpp.

template<typename T1 , typename T2 , int R, int C>

|  |  |  |  |
| --- | --- | --- | --- |
| Eigen::Matrix<typename boost::math::tools::promote\_args<T1,T2>::type,R,C> stan::math::add | ( | const Eigen::Matrix< T1, R, C > & | *m1*, |
|  |  | const Eigen::Matrix< T2, R, C > & | *m2* |
|  | ) |  |  |

inline

Return the sum of the specified matrices.

The two matrices must have the same dimensions.

Template Parameters
:   |  |  |
    | --- | --- |
    | T1 | Scalar type of first matrix. |
    | T2 | Scalar type of second matrix. |
    | R | Row type of matrices. |
    | C | Column type of matrices. |

Parameters
:   |  |  |
    | --- | --- |
    | m1 | First matrix. |
    | m2 | Second matrix. |

Returns
:   Sum of the matrices.

Exceptions
:   |  |  |
    | --- | --- |
    | std::domain\_error | if m1 and m2 do not have the same dimensions. |

Definition at line 27 of file add.hpp.

template<typename T1 , typename T2 , int R, int C>

|  |  |  |  |
| --- | --- | --- | --- |
| Eigen::Matrix<typename boost::math::tools::promote\_args<T1,T2>::type,R,C> stan::math::add | ( | const Eigen::Matrix< T1, R, C > & | *m*, |
|  |  | const T2 & | *c* |
|  | ) |  |  |

inline

Return the sum of the specified matrix and specified scalar.

Template Parameters
:   |  |  |
    | --- | --- |
    | T1 | Scalar type of matrix. |
    | T2 | Type of scalar. |

Parameters
:   |  |  |
    | --- | --- |
    | m | Matrix. |
    | c | Scalar. |

Returns
:   The matrix plus the scalar.

Definition at line 50 of file add.hpp.

template<typename T1 , typename T2 , int R, int C>

|  |  |  |  |
| --- | --- | --- | --- |
| Eigen::Matrix<typename boost::math::tools::promote\_args<T1,T2>::type,R,C> stan::math::add | ( | const T1 & | *c*, |
|  |  | const Eigen::Matrix< T2, R, C > & | *m* |
|  | ) |  |  |

inline

Return the sum of the specified scalar and specified matrix.

Template Parameters
:   |  |  |
    | --- | --- |
    | T1 | Type of scalar. |
    | T2 | Scalar type of matrix. |

Parameters
:   |  |  |
    | --- | --- |
    | c | Scalar. |
    | m | Matrix. |

Returns
:   The scalar plus the matrix.

Definition at line 71 of file add.hpp.

|  |  |  |  |
| --- | --- | --- | --- |
| void stan::math::add\_initial\_values | ( | const std::vector< stan::agrad::var > & | *y0*, |
|  |  | std::vector< std::vector< stan::agrad::var > > & | *y* |
|  | ) |  |  |

Increment the state derived from the coupled system in the with the original initial state.

This is necessary because the coupled system subtracts out the initial state in its representation when the initial state is unknown.

Parameters
:   |  |  |  |
    | --- | --- | --- |
    | [in] | y0 | original initial values to add back into the coupled system. |
    | [in,out] | y | state of the coupled system on input, incremented with initial values on output. |

Definition at line 34 of file coupled\_ode\_system.hpp.

template<typename T1 , typename T2 , int R1, int C1, int R2, int C2>

|  |  |  |  |
| --- | --- | --- | --- |
| Matrix<typename return\_type<T1, T2>::type, Dynamic, Dynamic> stan::math::append\_col | ( | const Matrix< T1, R1, C1 > & | *A*, |
|  |  | const Matrix< T2, R2, C2 > & | *B* |
|  | ) |  |  |

inline

Definition at line 23 of file append\_col.hpp.

template<typename T1 , typename T2 , int C1, int C2>

|  |  |  |  |
| --- | --- | --- | --- |
| Matrix<typename return\_type<T1, T2>::type, 1, Dynamic> stan::math::append\_col | ( | const Matrix< T1, 1, C1 > & | *A*, |
|  |  | const Matrix< T2, 1, C2 > & | *B* |
|  | ) |  |  |

inline

Definition at line 49 of file append\_col.hpp.

template<typename T , int R1, int C1, int R2, int C2>

|  |  |  |  |
| --- | --- | --- | --- |
| Matrix<T, Dynamic, Dynamic> stan::math::append\_col | ( | const Matrix< T, R1, C1 > & | *A*, |
|  |  | const Matrix< T, R2, C2 > & | *B* |
|  | ) |  |  |

inline

Definition at line 69 of file append\_col.hpp.

template<typename T , int C1, int C2>

|  |  |  |  |
| --- | --- | --- | --- |
| Matrix<T, 1, Dynamic> stan::math::append\_col | ( | const Matrix< T, 1, C1 > & | *A*, |
|  |  | const Matrix< T, 1, C2 > & | *B* |
|  | ) |  |  |

inline

Definition at line 85 of file append\_col.hpp.

template<typename T1 , typename T2 , int R1, int C1, int R2, int C2>

|  |  |  |  |
| --- | --- | --- | --- |
| Matrix<typename return\_type<T1, T2>::type, Dynamic, Dynamic> stan::math::append\_row | ( | const Matrix< T1, R1, C1 > & | *A*, |
|  |  | const Matrix< T2, R2, C2 > & | *B* |
|  | ) |  |  |

inline

Definition at line 23 of file append\_row.hpp.

template<typename T1 , typename T2 , int R1, int R2>

|  |  |  |  |
| --- | --- | --- | --- |
| Matrix<typename return\_type<T1, T2>::type, Dynamic, 1> stan::math::append\_row | ( | const Matrix< T1, R1, 1 > & | *A*, |
|  |  | const Matrix< T2, R1, 1 > & | *B* |
|  | ) |  |  |

inline

Definition at line 49 of file append\_row.hpp.

template<typename T , int R1, int C1, int R2, int C2>

|  |  |  |  |
| --- | --- | --- | --- |
| Matrix<T, Dynamic, Dynamic> stan::math::append\_row | ( | const Matrix< T, R1, C1 > & | *A*, |
|  |  | const Matrix< T, R2, C2 > & | *B* |
|  | ) |  |  |

inline

Definition at line 68 of file append\_row.hpp.

template<typename T , int R1, int R2>

|  |  |  |  |
| --- | --- | --- | --- |
| Matrix<T, Dynamic, 1> stan::math::append\_row | ( | const Matrix< T, R1, 1 > & | *A*, |
|  |  | const Matrix< T, R1, 1 > & | *B* |
|  | ) |  |  |

inline

Definition at line 84 of file append\_row.hpp.

template<typename T >

|  |  |  |  |  |  |
| --- | --- | --- | --- | --- | --- |
| bool stan::math::as\_bool | ( | const T | *x* | ) |  |

inline

Return 1 if the argument is unequal to zero and 0 otherwise.

Parameters
:   |  |  |
    | --- | --- |
    | x | Value. |

Returns
:   1 if argument is equal to zero (or NaN) and 0 otherwise.

Definition at line 14 of file as\_bool.hpp.

template<typename LHS , typename RHS >

|  |  |  |  |
| --- | --- | --- | --- |
| void stan::math::assign | ( | LHS & | *lhs*, |
|  |  | const RHS & | *rhs* |
|  | ) |  |  |

inline

Copy the right-hand side's value to the left-hand side variable.

The `assign()` function is overloaded. This instance will match arguments where the right-hand side is assignable to the left and they are not both `std::vector` or `Eigen::Matrix` types.

Template Parameters
:   |  |  |
    | --- | --- |
    | LHS | Type of left-hand side. |
    | RHS | Type of right-hand side. |

Parameters
:   |  |  |
    | --- | --- |
    | lhs | Left-hand side. |
    | rhs | Right-hand side. |

Definition at line 37 of file assign.hpp.

template<typename LHS , typename RHS , int R1, int C1, int R2, int C2>

|  |  |  |  |
| --- | --- | --- | --- |
| void stan::math::assign | ( | Eigen::Matrix< LHS, R1, C1 > & | *x*, |
|  |  | const Eigen::Matrix< RHS, R2, C2 > & | *y* |
|  | ) |  |  |

inline

Copy the right-hand side's value to the left-hand side variable.

The `assign()` function is overloaded. This instance will be called for arguments that are both `Eigen::Matrix` types, but whose shapes are not compatible. The shapes are specified in the row and column template parameters.

Template Parameters
:   |  |  |
    | --- | --- |
    | LHS | Type of left-hand side matrix elements. |
    | RHS | Type of right-hand side matrix elements. |
    | R1 | Row shape of left-hand side matrix. |
    | C1 | Column shape of left-hand side matrix. |
    | R2 | Row shape of right-hand side matrix. |
    | C2 | Column shape of right-hand side matrix. |

Parameters
:   |  |  |
    | --- | --- |
    | x | Left-hand side matrix. |
    | y | Right-hand side matrix. |

Exceptions
:   |  |  |
    | --- | --- |
    | std::domain\_error | if sizes do not match. |

Definition at line 63 of file assign.hpp.

template<typename LHS , typename RHS , int R, int C>

|  |  |  |  |
| --- | --- | --- | --- |
| void stan::math::assign | ( | Eigen::Matrix< LHS, R, C > & | *x*, |
|  |  | const Eigen::Matrix< RHS, R, C > & | *y* |
|  | ) |  |  |

inline

Copy the right-hand side's value to the left-hand side variable.

The `assign()` function is overloaded. This instance will be called for arguments that are both `Eigen::Matrix` types and whose shapes match. The shapes are specified in the row and column template parameters.

Template Parameters
:   |  |  |
    | --- | --- |
    | LHS | Type of left-hand side matrix elements. |
    | RHS | Type of right-hand side matrix elements. |
    | R | Row shape of both matrices. |
    | C | Column shape of both mtarices. |

Parameters
:   |  |  |
    | --- | --- |
    | x | Left-hand side matrix. |
    | y | Right-hand side matrix. |

Exceptions
:   |  |  |
    | --- | --- |
    | std::domain\_error | if sizes do not match. |

Definition at line 94 of file assign.hpp.

template<typename LHS , typename RHS , int R, int C>

|  |  |  |  |
| --- | --- | --- | --- |
| void stan::math::assign | ( | Eigen::Block< LHS > | *x*, |
|  |  | const Eigen::Matrix< RHS, R, C > & | *y* |
|  | ) |  |  |

inline

Copy the right-hand side's value to the left-hand side variable.

The `assign()` function is overloaded. This instance will be called for arguments that are both `Eigen::Matrix` types and whose shapes match. The shape of the right-hand side matrix is specified in the row and column shape template parameters.

Template Parameters
:   |  |  |
    | --- | --- |
    | LHS | Type of matrix block elements. |
    | RHS | Type of right-hand side matrix elements. |
    | R | Row shape for right-hand side matrix. |
    | C | Column shape for right-hand side matrix. |

Parameters
:   |  |  |
    | --- | --- |
    | x | Left-hand side block view of matrix. |
    | y | Right-hand side matrix. |

Exceptions
:   |  |  |
    | --- | --- |
    | std::domain\_error | if sizes do not match. |

Definition at line 122 of file assign.hpp.

template<typename LHS , typename RHS >

|  |  |  |  |
| --- | --- | --- | --- |
| void stan::math::assign | ( | std::vector< LHS > & | *x*, |
|  |  | const std::vector< RHS > & | *y* |
|  | ) |  |  |

inline

Copy the right-hand side's value to the left-hand side variable.

The `assign()` function is overloaded. This instance will be called for arguments that are both `std::vector`, and will call `assign()` element-by element.

For example, a `std::vector<int>` can be assigned to a `std::vector<double>` using this function.

Template Parameters
:   |  |  |
    | --- | --- |
    | LHS | Type of left-hand side vector elements. |
    | RHS | Type of right-hand side vector elements. |

Parameters
:   |  |  |
    | --- | --- |
    | x | Left-hand side vector. |
    | y | Right-hand side vector. |

Exceptions
:   |  |  |
    | --- | --- |
    | std::domain\_error | if sizes do not match. |

Definition at line 153 of file assign.hpp.

template<typename T2 >

|  |  |  |  |
| --- | --- | --- | --- |
| T2 stan::math::bessel\_first\_kind | ( | const int | *v*, |
|  |  | const T2 | *z* |
|  | ) |  |  |

inline

Definition at line 40 of file bessel\_first\_kind.hpp.

template<typename T2 >

|  |  |  |  |
| --- | --- | --- | --- |
| T2 stan::math::bessel\_second\_kind | ( | const int | *v*, |
|  |  | const T2 | *z* |
|  | ) |  |  |

inline

Definition at line 40 of file bessel\_second\_kind.hpp.

template<typename T >

|  |  |  |  |
| --- | --- | --- | --- |
| boost::math::tools::promote\_args<T>::type stan::math::binary\_log\_loss | ( | const int | *y*, |
|  |  | const T | *y\_hat* |
|  | ) |  |  |

inline

Returns the log loss function for binary classification with specified reference and response values.

The log loss function for prediction  given outcome  is

, and

.

Parameters
:   |  |  |
    | --- | --- |
    | y | Reference value in { 0 , 1 }. |
    | y\_hat | Response value in [0,1]. |

Returns
:   Log loss for response given reference value.

Definition at line 26 of file binary\_log\_loss.hpp.

template<typename T\_N , typename T\_n >

|  |  |  |  |
| --- | --- | --- | --- |
| boost::math::tools::promote\_args<T\_N, T\_n>::type stan::math::binomial\_coefficient\_log | ( | const T\_N | *N*, |
|  |  | const T\_n | *n* |
|  | ) |  |  |

inline

Return the log of the binomial coefficient for the specified arguments.

The binomial coefficient, , read "N choose n", is defined for  by

.

This function uses Gamma functions to define the log and generalize the arguments to continuous N and n.

.

Parameters
:   |  |  |
    | --- | --- |
    | N | total number of objects. |
    | n | number of objects chosen. |

Returns
:   log (N choose n).

Definition at line 63 of file binomial\_coefficient\_log.hpp.

template<typename T >

|  |  |  |  |
| --- | --- | --- | --- |
| Eigen::Matrix<T,Eigen::Dynamic,Eigen::Dynamic> stan::math::block | ( | const Eigen::Matrix< T, Eigen::Dynamic, Eigen::Dynamic > & | *m*, |
|  |  | size\_t | *i*, |
|  |  | size\_t | *j*, |
|  |  | size\_t | *nrows*, |
|  |  | size\_t | *ncols* |
|  | ) |  |  |

inline

Return a nrows x ncols submatrix starting at (i-1,j-1).

Parameters
:   |  |  |
    | --- | --- |
    | m | Matrix |
    | i | Starting row |
    | j | Starting column |
    | nrows | Number of rows in block |
    | ncols | Number of columns in block |

Definition at line 23 of file block.hpp.

template<typename T\_y , typename T\_low , typename T\_high , typename T\_result >

|  |  |  |  |
| --- | --- | --- | --- |
| bool stan::math::check\_bounded | ( | const char \* | *function*, |
|  |  | const T\_y & | *y*, |
|  |  | const T\_low & | *low*, |
|  |  | const T\_high & | *high*, |
|  |  | const char \* | *name*, |
|  |  | T\_result \* | *result* |
|  | ) |  |  |

inline

Definition at line 79 of file check\_bounded.hpp.

template<typename T\_y , typename T\_result >

|  |  |  |  |
| --- | --- | --- | --- |
| bool stan::math::check\_cholesky\_factor | ( | const char \* | *function*, |
|  |  | const Eigen::Matrix< T\_y, Eigen::Dynamic, Eigen::Dynamic > & | *y*, |
|  |  | const char \* | *name*, |
|  |  | T\_result \* | *result* |
|  | ) |  |  |

inline

Return `true` if the specified matrix is a valid Cholesky factor.

A Cholesky factor is a lower triangular matrix whose diagonal elements are all positive. Note that Cholesky factors need not be square, but require at least as many rows M as columns N (i.e., M >= N).

Parameters
:   |  |  |
    | --- | --- |
    | function |  |
    | y | Matrix to test. |
    | name |  |
    | result |  |

Returns
:   `true` if the matrix is a valid Cholesky factor.
:   throws if any element in matrix is nan

Template Parameters
:   |  |  |
    | --- | --- |
    | T\_y | Type of elements of Cholesky factor |
    | T\_result | Type of result. |

Definition at line 30 of file check\_cholesky\_factor.hpp.

template<typename T\_y , typename T\_result >

|  |  |  |  |
| --- | --- | --- | --- |
| bool stan::math::check\_cholesky\_factor\_corr | ( | const char \* | *function*, |
|  |  | const Eigen::Matrix< T\_y, Eigen::Dynamic, Eigen::Dynamic > & | *y*, |
|  |  | const char \* | *name*, |
|  |  | T\_result \* | *result* |
|  | ) |  |  |

Return `true` if the specified matrix is a valid Cholesky factor.

A Cholesky factor is a lower triangular matrix whose diagonal elements are all positive. Note that Cholesky factors need not be square, but require at least as many rows M as columns N (i.e., M >= N).

Tolerance is specified by `math::CONSTRAINT_TOLERANCE`.

Parameters
:   |  |  |
    | --- | --- |
    | function |  |
    | y | Matrix to test. |
    | name |  |
    | result |  |

Returns
:   `true` if the matrix is a valid Cholesky factor.
:   throws if any element in y is nan.

Template Parameters
:   |  |  |
    | --- | --- |
    | T\_y | Type of elements of Cholesky factor |
    | T\_result | Type of result. |

Definition at line 34 of file check\_cholesky\_factor\_corr.hpp.

template<typename T >

|  |  |  |  |
| --- | --- | --- | --- |
| bool stan::math::check\_cholesky\_factor\_corr | ( | const char \* | *function*, |
|  |  | const Eigen::Matrix< T, Eigen::Dynamic, Eigen::Dynamic > & | *y*, |
|  |  | const char \* | *name*, |
|  |  | T \* | *result* = `0` |
|  | ) |  |  |

inline

Definition at line 54 of file check\_cholesky\_factor\_corr.hpp.

template<typename T\_y , typename T\_result >

|  |  |  |  |
| --- | --- | --- | --- |
| bool stan::math::check\_cholesky\_factor\_corr | ( | const char \* | *function*, |
|  |  | const Eigen::Matrix< T\_y, Eigen::Dynamic, Eigen::Dynamic > & | *y*, |
|  |  | T\_result \* | *result* |
|  | ) |  |  |

inline

Return `true` if the specified matrix is a valid Cholesky factor (lower triangular, positive diagonal).

Parameters
:   |  |  |
    | --- | --- |
    | function | Name of function. |
    | y | Matrix to test. |
    | result | Pointer into which to write result. |

Returns
:   `true` if the matrix is a valid Cholesky factor.
:   throws if any element in matrix is nan

Template Parameters
:   |  |  |
    | --- | --- |
    | T\_y | Type of elements of Cholesky factor |
    | T\_result | Type of result. |

Definition at line 75 of file check\_cholesky\_factor\_corr.hpp.

template<typename T >

|  |  |  |  |
| --- | --- | --- | --- |
| bool stan::math::check\_cholesky\_factor\_corr | ( | const char \* | *function*, |
|  |  | const Eigen::Matrix< T, Eigen::Dynamic, Eigen::Dynamic > & | *y*, |
|  |  | T \* | *result* = `0` |
|  | ) |  |  |

inline

Definition at line 83 of file check\_cholesky\_factor\_corr.hpp.

template<typename T\_y , typename T\_result , int R, int C>

|  |  |  |  |
| --- | --- | --- | --- |
| bool stan::math::check\_column\_index | ( | const char \* | *function*, |
|  |  | size\_t | *i*, |
|  |  | const Eigen::Matrix< T\_y, R, C > & | *y*, |
|  |  | const char \* | *name*, |
|  |  | T\_result \* | *result* |
|  | ) |  |  |

inline

Return `true` if the specified index is a valid column of the matrix.

NOTE: this will not throw if y contains nan values.

Parameters
:   |  |  |
    | --- | --- |
    | function |  |
    | i | is index |
    | y | Matrix to test against |
    | name |  |
    | result |  |

Returns
:   `true` if the index is a valid column index of the matrix.

Template Parameters
:   |  |  |
    | --- | --- |
    | T | Type of scalar. |

Definition at line 25 of file check\_column\_index.hpp.

template<typename T , typename T\_result >

|  |  |  |  |
| --- | --- | --- | --- |
| bool stan::math::check\_consistent\_size | ( | size\_t | *max\_size*, |
|  |  | const char \* | *function*, |
|  |  | const T & | *x*, |
|  |  | const char \* | *name*, |
|  |  | T\_result \* | *result* |
|  | ) |  |  |

inline

Definition at line 13 of file check\_consistent\_size.hpp.

template<typename T1 , typename T2 , typename T\_result >

|  |  |  |  |
| --- | --- | --- | --- |
| bool stan::math::check\_consistent\_sizes | ( | const char \* | *function*, |
|  |  | const T1 & | *x1*, |
|  |  | const T2 & | *x2*, |
|  |  | const char \* | *name1*, |
|  |  | const char \* | *name2*, |
|  |  | T\_result \* | *result* |
|  | ) |  |  |

inline

Definition at line 13 of file check\_consistent\_sizes.hpp.

template<typename T1 , typename T2 , typename T3 , typename T\_result >

|  |  |  |  |
| --- | --- | --- | --- |
| bool stan::math::check\_consistent\_sizes | ( | const char \* | *function*, |
|  |  | const T1 & | *x1*, |
|  |  | const T2 & | *x2*, |
|  |  | const T3 & | *x3*, |
|  |  | const char \* | *name1*, |
|  |  | const char \* | *name2*, |
|  |  | const char \* | *name3*, |
|  |  | T\_result \* | *result* |
|  | ) |  |  |

inline

Definition at line 26 of file check\_consistent\_sizes.hpp.

template<typename T1 , typename T2 , typename T3 , typename T4 , typename T\_result >

|  |  |  |  |
| --- | --- | --- | --- |
| bool stan::math::check\_consistent\_sizes | ( | const char \* | *function*, |
|  |  | const T1 & | *x1*, |
|  |  | const T2 & | *x2*, |
|  |  | const T3 & | *x3*, |
|  |  | const T4 & | *x4*, |
|  |  | const char \* | *name1*, |
|  |  | const char \* | *name2*, |
|  |  | const char \* | *name3*, |
|  |  | const char \* | *name4*, |
|  |  | T\_result \* | *result* |
|  | ) |  |  |

inline

Definition at line 41 of file check\_consistent\_sizes.hpp.

template<typename T\_y , typename T\_result >

|  |  |  |  |
| --- | --- | --- | --- |
| bool stan::math::check\_corr\_matrix | ( | const char \* | *function*, |
|  |  | const Eigen::Matrix< T\_y, Eigen::Dynamic, Eigen::Dynamic > & | *y*, |
|  |  | const char \* | *name*, |
|  |  | T\_result \* | *result* |
|  | ) |  |  |

inline

Return `true` if the specified matrix is a valid correlation matrix.

A valid correlation matrix is symmetric, has a unit diagonal (all 1 values), and has all values between -1 and 1 (inclussive).

Parameters
:   |  |  |
    | --- | --- |
    | function |  |
    | y | Matrix to test. |
    | name |  |
    | result |  |

Returns
:   `true` if the specified matrix is a valid correlation matrix.
:   throw if any element in matrix is nan

Template Parameters
:   |  |  |
    | --- | --- |
    | T | Type of scalar. |

Definition at line 38 of file check\_corr\_matrix.hpp.

template<typename T\_y , typename T\_result >

|  |  |  |  |
| --- | --- | --- | --- |
| bool stan::math::check\_cov\_matrix | ( | const char \* | *function*, |
|  |  | const Eigen::Matrix< T\_y, Eigen::Dynamic, Eigen::Dynamic > & | *y*, |
|  |  | const char \* | *name*, |
|  |  | T\_result \* | *result* |
|  | ) |  |  |

inline

Return `true` if the specified matrix is a valid covariance matrix.

A valid covariance matrix must be square, symmetric, and positive definite.

Parameters
:   |  |  |
    | --- | --- |
    | function |  |
    | y | Matrix to test. |
    | name |  |
    | result |  |

Returns
:   `true` if the matrix is a valid covariance matrix.
:   throws if any element in matrix is nan

Template Parameters
:   |  |  |
    | --- | --- |
    | T | Type of scalar. |

Definition at line 29 of file check\_cov\_matrix.hpp.

template<typename T\_y , typename T\_eq , typename T\_result >

|  |  |  |  |
| --- | --- | --- | --- |
| bool stan::math::check\_equal | ( | const char \* | *function*, |
|  |  | const T\_y & | *y*, |
|  |  | const T\_eq & | *eq*, |
|  |  | const char \* | *name*, |
|  |  | T\_result \* | *result* |
|  | ) |  |  |

inline

Definition at line 57 of file check\_equal.hpp.

template<typename T\_y , typename T\_result >

|  |  |  |  |
| --- | --- | --- | --- |
| bool stan::math::check\_finite | ( | const char \* | *function*, |
|  |  | const T\_y & | *y*, |
|  |  | const char \* | *name*, |
|  |  | T\_result \* | *result* |
|  | ) |  |  |

inline

Checks if the variable y is finite.

NOTE: throws if any element in y is nan.

Definition at line 52 of file check\_finite.hpp.

template<typename T\_y , typename T\_low , typename T\_result >

|  |  |  |  |
| --- | --- | --- | --- |
| bool stan::math::check\_greater | ( | const char \* | *function*, |
|  |  | const T\_y & | *y*, |
|  |  | const T\_low & | *low*, |
|  |  | const char \* | *name*, |
|  |  | T\_result \* | *result* |
|  | ) |  |  |

inline

Definition at line 58 of file check\_greater.hpp.

template<typename T\_y , typename T\_low , typename T\_result >

|  |  |  |  |
| --- | --- | --- | --- |
| bool stan::math::check\_greater\_or\_equal | ( | const char \* | *function*, |
|  |  | const T\_y & | *y*, |
|  |  | const T\_low & | *low*, |
|  |  | const char \* | *name*, |
|  |  | T\_result \* | *result* |
|  | ) |  |  |

inline

Definition at line 58 of file check\_greater\_or\_equal.hpp.

template<typename T , int R, int C, typename T\_result >

|  |  |  |  |
| --- | --- | --- | --- |
| bool stan::math::check\_ldlt\_factor | ( | const char \* | *function*, |
|  |  | stan::math::LDLT\_factor< T, R, C > & | *A*, |
|  |  | const char \* | *name*, |
|  |  | T\_result \* | *result* |
|  | ) |  |  |

inline

Return `true` if the underlying matrix is positive definite.

Parameters
:   |  |  |
    | --- | --- |
    | function |  |
    | A |  |
    | name |  |
    | result |  |

Returns
:   `true` if the matrix is positive definite.
:   throws if any element in lower triangular of matrix is nan

Template Parameters
:   |  |  |
    | --- | --- |
    | T | Type of scalar. |

Definition at line 24 of file check\_ldlt\_factor.hpp.

template<typename T\_y , typename T\_high , typename T\_result >

|  |  |  |  |
| --- | --- | --- | --- |
| bool stan::math::check\_less | ( | const char \* | *function*, |
|  |  | const T\_y & | *y*, |
|  |  | const T\_high & | *high*, |
|  |  | const char \* | *name*, |
|  |  | T\_result \* | *result* |
|  | ) |  |  |

inline

Definition at line 52 of file check\_less.hpp.

template<typename T\_y , typename T\_high , typename T\_result >

|  |  |  |  |
| --- | --- | --- | --- |
| bool stan::math::check\_less\_or\_equal | ( | const char \* | *function*, |
|  |  | const T\_y & | *y*, |
|  |  | const T\_high & | *high*, |
|  |  | const char \* | *name*, |
|  |  | T\_result \* | *result* |
|  | ) |  |  |

inline

Definition at line 52 of file check\_less\_or\_equal.hpp.

template<typename T\_y , typename T\_result >

|  |  |  |  |
| --- | --- | --- | --- |
| bool stan::math::check\_lower\_triangular | ( | const char \* | *function*, |
|  |  | const Eigen::Matrix< T\_y, Eigen::Dynamic, Eigen::Dynamic > & | *y*, |
|  |  | const char \* | *name*, |
|  |  | T\_result \* | *result* |
|  | ) |  |  |

inline

Return `true` if the specified matrix is lower triangular.

A matrix x is not lower triangular if there is a non-zero entry x[m,n] with m < n.

Parameters
:   |  |  |
    | --- | --- |
    | function |  |
    | y | Matrix to test. |
    | name |  |
    | result |  |

Returns
:   `true` if the matrix is symmetric.
:   throws if any element in upper triangular is nan

Template Parameters
:   |  |  |
    | --- | --- |
    | T | Type of scalar. |

Definition at line 27 of file check\_lower\_triangular.hpp.

template<typename T1 , typename T2 , int R1, int C1, int R2, int C2, typename T\_result >

|  |  |  |  |
| --- | --- | --- | --- |
| bool stan::math::check\_matching\_dims | ( | const char \* | *function*, |
|  |  | const Eigen::Matrix< T1, R1, C1 > & | *y1*, |
|  |  | const char \* | *name1*, |
|  |  | const Eigen::Matrix< T2, R2, C2 > & | *y2*, |
|  |  | const char \* | *name2*, |
|  |  | T\_result \* | *result* |
|  | ) |  |  |

inline

Definition at line 15 of file check\_matching\_dims.hpp.

template<typename T\_y1 , typename T\_y2 , typename T\_result >

|  |  |  |  |
| --- | --- | --- | --- |
| bool stan::math::check\_matching\_sizes | ( | const char \* | *function*, |
|  |  | const T\_y1 & | *y1*, |
|  |  | const char \* | *name1*, |
|  |  | const T\_y2 & | *y2*, |
|  |  | const char \* | *name2*, |
|  |  | T\_result \* | *result* |
|  | ) |  |  |

inline

Definition at line 15 of file check\_matching\_sizes.hpp.

template<typename T1 , typename T2 , typename T\_result >

|  |  |  |  |
| --- | --- | --- | --- |
| bool stan::math::check\_multiplicable | ( | const char \* | *function*, |
|  |  | const T1 & | *y1*, |
|  |  | const char \* | *name1*, |
|  |  | const T2 & | *y2*, |
|  |  | const char \* | *name2*, |
|  |  | T\_result \* | *result* |
|  | ) |  |  |

inline

Definition at line 15 of file check\_multiplicable.hpp.

template<typename T\_y , typename T\_result >

|  |  |  |  |
| --- | --- | --- | --- |
| bool stan::math::check\_nonnegative | ( | const char \* | *function*, |
|  |  | const T\_y & | *y*, |
|  |  | const char \* | *name*, |
|  |  | T\_result \* | *result* |
|  | ) |  |  |

inline

Definition at line 52 of file check\_nonnegative.hpp.

template<typename T\_y , typename T\_result >

|  |  |  |  |
| --- | --- | --- | --- |
| bool stan::math::check\_nonzero\_size | ( | const char \* | *function*, |
|  |  | const T\_y & | *y*, |
|  |  | const char \* | *name*, |
|  |  | T\_result \* | *result* |
|  | ) |  |  |

inline

Return `true` if the specified matrix/vector is of non-zero size.

Throws a std:domain\_error otherwise. The message will indicate that the variable name "has size 0".

NOTE: this will not throw if y contains nan values.

Parameters
:   |  |  |
    | --- | --- |
    | function |  |
    | y | matrix/vector to test against |
    | name |  |
    | result |  |

Returns
:   `true` if the the specified matrix/vector is of non-zero size

Exceptions
:   |  |  |
    | --- | --- |
    | std::domain\_error | if the specified matrix/vector is of non-zero size |

Template Parameters
:   |  |  |
    | --- | --- |
    | T | Type of scalar. |

Definition at line 33 of file check\_nonzero\_size.hpp.

template<typename T\_y , typename T\_result >

|  |  |  |  |
| --- | --- | --- | --- |
| bool stan::math::check\_not\_nan | ( | const char \* | *function*, |
|  |  | const T\_y & | *y*, |
|  |  | const char \* | *name*, |
|  |  | T\_result \* | *result* |
|  | ) |  |  |

inline

Checks if the variable y is nan.

Parameters
:   |  |  |
    | --- | --- |
    | function | Name of function being invoked. |
    | y | Reference to variable being tested. |
    | name | Name of variable being tested. |
    | result | Pointer to resulting value after test. |

Template Parameters
:   |  |  |
    | --- | --- |
    | T\_y | Type of variable being tested. |
    | T\_result | Type of result returned. |

Definition at line 56 of file check\_not\_nan.hpp.

template<typename T\_y , typename T\_result >

|  |  |  |  |
| --- | --- | --- | --- |
| bool stan::math::check\_ordered | ( | const char \* | *function*, |
|  |  | const Eigen::Matrix< T\_y, Eigen::Dynamic, 1 > & | *y*, |
|  |  | const char \* | *name*, |
|  |  | T\_result \* | *result* |
|  | ) |  |  |

Return `true` if the specified vector is sorted into increasing order.

There may not be duplicate values. Otherwise, raise a domain error according to the specified policy.

Parameters
:   |  |  |
    | --- | --- |
    | function |  |
    | y | Vector to test. |
    | name |  |
    | result |  |

Returns
:   `true` if the vector has positive, ordered values.
:   throws if any element in y is nan

Definition at line 29 of file check\_ordered.hpp.

template<typename T\_y , typename T\_result >

|  |  |  |  |
| --- | --- | --- | --- |
| bool stan::math::check\_ordered | ( | const char \* | *function*, |
|  |  | const std::vector< T\_y > & | *y*, |
|  |  | const char \* | *name*, |
|  |  | T\_result \* | *result* |
|  | ) |  |  |

Definition at line 57 of file check\_ordered.hpp.

template<typename T >

|  |  |  |  |
| --- | --- | --- | --- |
| bool stan::math::check\_ordered | ( | const char \* | *function*, |
|  |  | const Eigen::Matrix< T, Eigen::Dynamic, 1 > & | *y*, |
|  |  | const char \* | *name*, |
|  |  | T \* | *result* = `0` |
|  | ) |  |  |

Definition at line 81 of file check\_ordered.hpp.

template<typename T >

|  |  |  |  |
| --- | --- | --- | --- |
| bool stan::math::check\_ordered | ( | const char \* | *function*, |
|  |  | const std::vector< T > & | *y*, |
|  |  | const char \* | *name*, |
|  |  | T \* | *result* = `0` |
|  | ) |  |  |

Definition at line 88 of file check\_ordered.hpp.

template<typename T\_y , typename T\_result >

|  |  |  |  |
| --- | --- | --- | --- |
| bool stan::math::check\_pos\_definite | ( | const char \* | *function*, |
|  |  | const Eigen::Matrix< T\_y, Eigen::Dynamic, Eigen::Dynamic > & | *y*, |
|  |  | const char \* | *name*, |
|  |  | T\_result \* | *result* |
|  | ) |  |  |

inline

Return `true` if the specified matrix is positive definite.

NOTE: symmetry is NOT checked by this function

Parameters
:   |  |  |
    | --- | --- |
    | function |  |
    | y | Matrix to test. |
    | name |  |
    | result |  |

Returns
:   `true` if the matrix is positive definite.
:   throws if any element in lower triangular of matrix is nan.

Template Parameters
:   |  |  |
    | --- | --- |
    | T | Type of scalar. |

Definition at line 29 of file check\_pos\_definite.hpp.

template<typename T\_y , typename T\_result >

|  |  |  |  |
| --- | --- | --- | --- |
| bool stan::math::check\_pos\_semidefinite | ( | const char \* | *function*, |
|  |  | const Eigen::Matrix< T\_y, Eigen::Dynamic, Eigen::Dynamic > & | *y*, |
|  |  | const char \* | *name*, |
|  |  | T\_result \* | *result* |
|  | ) |  |  |

inline

Return `true` if the specified matrix is positive definite.

NOTE: symmetry is NOT checked by this function

Parameters
:   |  |  |
    | --- | --- |
    | function |  |
    | y | Matrix to test. |
    | name |  |
    | result |  |

Returns
:   `true` if the matrix is positive semi-definite.
:   throws if any element in y is nan

Template Parameters
:   |  |  |
    | --- | --- |
    | T | Type of scalar. |

Definition at line 33 of file check\_pos\_semidefinite.hpp.

template<typename T\_y , typename T\_result >

|  |  |  |  |
| --- | --- | --- | --- |
| bool stan::math::check\_positive | ( | const char \* | *function*, |
|  |  | const T\_y & | *y*, |
|  |  | const char \* | *name*, |
|  |  | T\_result \* | *result* |
|  | ) |  |  |

inline

Definition at line 54 of file check\_positive.hpp.

template<typename T\_y , typename T\_result >

|  |  |  |  |
| --- | --- | --- | --- |
| bool stan::math::check\_positive\_finite | ( | const char \* | *function*, |
|  |  | const T\_y & | *y*, |
|  |  | const char \* | *name*, |
|  |  | T\_result \* | *result* |
|  | ) |  |  |

inline

Definition at line 12 of file check\_positive\_finite.hpp.

template<typename T\_y , typename T\_result >

|  |  |  |  |
| --- | --- | --- | --- |
| bool stan::math::check\_positive\_ordered | ( | const char \* | *function*, |
|  |  | const Eigen::Matrix< T\_y, Eigen::Dynamic, 1 > & | *y*, |
|  |  | const char \* | *name*, |
|  |  | T\_result \* | *result* |
|  | ) |  |  |

Return `true` if the specified vector contains only non-negative values and is sorted into increasing order.

There may not be duplicate values. Otherwise, raise a domain error according to the specified policy.

Parameters
:   |  |  |
    | --- | --- |
    | function |  |
    | y | Vector to test. |
    | name |  |
    | result |  |

Returns
:   `true` if the vector has positive, ordered
:   throws if any element in y is nan values.

Definition at line 29 of file check\_positive\_ordered.hpp.

|  |  |  |  |
| --- | --- | --- | --- |
| void stan::math::check\_range | ( | size\_t | *max*, |
|  |  | size\_t | *i*, |
|  |  | const char \* | *msg*, |
|  |  | size\_t | *idx* |
|  | ) |  |  |

inline

Definition at line 30 of file check\_range.hpp.

template<typename T\_y , typename T\_result , int R, int C>

|  |  |  |  |
| --- | --- | --- | --- |
| bool stan::math::check\_row\_index | ( | const char \* | *function*, |
|  |  | size\_t | *i*, |
|  |  | const Eigen::Matrix< T\_y, R, C > & | *y*, |
|  |  | const char \* | *name*, |
|  |  | T\_result \* | *result* |
|  | ) |  |  |

inline

Return `true` if the specified index is a valid row of the matrix.

NOTE: this will not throw if y contains nan values.

Parameters
:   |  |  |
    | --- | --- |
    | function |  |
    | i | is index |
    | y | Matrix to test against |
    | name |  |
    | result |  |

Returns
:   `true` if the index is a valid row index of the matrix.

Template Parameters
:   |  |  |
    | --- | --- |
    | T | Type of scalar. |

Definition at line 25 of file check\_row\_index.hpp.

template<typename T\_prob , typename T\_result >

|  |  |  |  |
| --- | --- | --- | --- |
| bool stan::math::check\_simplex | ( | const char \* | *function*, |
|  |  | const Eigen::Matrix< T\_prob, Eigen::Dynamic, 1 > & | *theta*, |
|  |  | const char \* | *name*, |
|  |  | T\_result \* | *result* |
|  | ) |  |  |

Return `true` if the specified vector is simplex.

To be a simplex, all values must be greater than or equal to 0 and the values must sum to 1.

The test that the values sum to 1 is done to within the tolerance specified by `CONSTRAINT_TOLERANCE`.

Parameters
:   |  |  |
    | --- | --- |
    | function |  |
    | theta | Vector to test. |
    | name |  |
    | result |  |

Returns
:   `true` if the vector is a simplex.
:   throws if any element is nan.

Definition at line 32 of file check\_simplex.hpp.

template<typename T\_size1 , typename T\_size2 , typename T\_result >

|  |  |  |  |
| --- | --- | --- | --- |
| bool stan::math::check\_size\_match | ( | const char \* | *function*, |
|  |  | T\_size1 | *i*, |
|  |  | const char \* | *name\_i*, |
|  |  | T\_size2 | *j*, |
|  |  | const char \* | *name\_j*, |
|  |  | T\_result \* | *result* |
|  | ) |  |  |

inline

Definition at line 13 of file check\_size\_match.hpp.

template<typename T\_y , typename T\_result >

|  |  |  |  |
| --- | --- | --- | --- |
| bool stan::math::check\_spsd\_matrix | ( | const char \* | *function*, |
|  |  | const Eigen::Matrix< T\_y, Eigen::Dynamic, Eigen::Dynamic > & | *y*, |
|  |  | const char \* | *name*, |
|  |  | T\_result \* | *result* |
|  | ) |  |  |

inline

Return `true` if the specified matrix is a square, symmetric, and positive semi-definite.

Parameters
:   |  |  |
    | --- | --- |
    | function |  |
    | y | Matrix to test. |
    | name |  |
    | result |  |

Returns
:   `true` if the matrix is a square, symmetric, and positive semi-definite.
:   throws if any element in matrix is nan.

Template Parameters
:   |  |  |
    | --- | --- |
    | T | Type of scalar. |

Definition at line 29 of file check\_spsd\_matrix.hpp.

template<typename T\_y , typename T\_result >

|  |  |  |  |
| --- | --- | --- | --- |
| bool stan::math::check\_square | ( | const char \* | *function*, |
|  |  | const Eigen::Matrix< T\_y, Eigen::Dynamic, Eigen::Dynamic > & | *y*, |
|  |  | const char \* | *name*, |
|  |  | T\_result \* | *result* |
|  | ) |  |  |

inline

Return `true` if the specified matrix is square.

NOTE: this will not throw if any elements in y are nan.

Parameters
:   |  |  |
    | --- | --- |
    | function |  |
    | y | Matrix to test. |
    | name |  |
    | result |  |

Returns
:   `true` if the matrix is a square matrix.

Template Parameters
:   |  |  |
    | --- | --- |
    | T | Type of scalar. |

Definition at line 24 of file check\_square.hpp.

template<typename T\_y , typename T\_result >

|  |  |  |  |
| --- | --- | --- | --- |
| bool stan::math::check\_std\_vector\_index | ( | const char \* | *function*, |
|  |  | size\_t | *i*, |
|  |  | const std::vector< T\_y > & | *y*, |
|  |  | const char \* | *name*, |
|  |  | T\_result \* | *result* |
|  | ) |  |  |

inline

Return `true` if the specified index is valid in std vector.

NOTE: this will not throw if y contains nan values.

Parameters
:   |  |  |
    | --- | --- |
    | function |  |
    | i | is index |
    | y | std vector to test against |
    | name |  |
    | result |  |

Returns
:   `true` if the index is a valid in std vector.

Template Parameters
:   |  |  |
    | --- | --- |
    | T | Type of scalar. |

Definition at line 25 of file check\_std\_vector\_index.hpp.

template<typename T\_y , typename T\_result >

|  |  |  |  |
| --- | --- | --- | --- |
| bool stan::math::check\_symmetric | ( | const char \* | *function*, |
|  |  | const Eigen::Matrix< T\_y, Eigen::Dynamic, Eigen::Dynamic > & | *y*, |
|  |  | const char \* | *name*, |
|  |  | T\_result \* | *result* |
|  | ) |  |  |

inline

Return `true` if the specified matrix is symmetric.

NOTE: squareness is not checked by this function

Parameters
:   |  |  |
    | --- | --- |
    | function |  |
    | y | Matrix to test. |
    | name |  |
    | result |  |

Returns
:   `true` if the matrix is symmetric.
:   throws if any element not on the main diagonal is NaN

Template Parameters
:   |  |  |
    | --- | --- |
    | T | Type of scalar. |

Definition at line 31 of file check\_symmetric.hpp.

template<typename T\_prob , typename T\_result >

|  |  |  |  |
| --- | --- | --- | --- |
| bool stan::math::check\_unit\_vector | ( | const char \* | *function*, |
|  |  | const Eigen::Matrix< T\_prob, Eigen::Dynamic, 1 > & | *theta*, |
|  |  | const char \* | *name*, |
|  |  | T\_result \* | *result* |
|  | ) |  |  |

Return `true` if the specified vector is unit vector.

The test that the values sum to 1 is done to within the tolerance specified by `CONSTRAINT_TOLERANCE`.

Parameters
:   |  |  |
    | --- | --- |
    | function |  |
    | theta | Vector to test. |
    | name |  |
    | result |  |

Returns
:   `true` if the vector is a unit vector.
:   throws if any element in theta is nan

Definition at line 27 of file check\_unit\_vector.hpp.

template<typename T >

|  |  |  |  |
| --- | --- | --- | --- |
| bool stan::math::check\_unit\_vector | ( | const char \* | *function*, |
|  |  | const Eigen::Matrix< T, Eigen::Dynamic, 1 > & | *theta*, |
|  |  | const char \* | *name*, |
|  |  | T \* | *result* = `0` |
|  | ) |  |  |

inline

Definition at line 55 of file check\_unit\_vector.hpp.

template<typename T , int R, int C, typename T\_result >

|  |  |  |  |
| --- | --- | --- | --- |
| bool stan::math::check\_vector | ( | const char \* | *function*, |
|  |  | const Eigen::Matrix< T, R, C > & | *x*, |
|  |  | const char \* | *name*, |
|  |  | T\_result \* | *result* |
|  | ) |  |  |

inline

Definition at line 14 of file check\_vector.hpp.

template<typename T >

|  |  |  |  |  |  |
| --- | --- | --- | --- | --- | --- |
| Eigen::Matrix<T,Eigen::Dynamic,Eigen::Dynamic> stan::math::cholesky\_decompose | ( | const Eigen::Matrix< T, Eigen::Dynamic, Eigen::Dynamic > & | *m* | ) |  |

Return the lower-triangular Cholesky factor (i.e., matrix square root) of the specified square, symmetric matrix.

The return value  will be a lower-traingular matrix such that the original matrix  is given by

.

Parameters
:   |  |  |
    | --- | --- |
    | m | Symmetrix matrix. |

Returns
:   Square root of matrix.

Exceptions
:   |  |  |
    | --- | --- |
    | std::domain\_error | if m is not a symmetric matrix. |

Definition at line 23 of file cholesky\_decompose.hpp.

template<typename T >

|  |  |  |  |
| --- | --- | --- | --- |
| Eigen::Matrix<T,Eigen::Dynamic,1> stan::math::col | ( | const Eigen::Matrix< T, Eigen::Dynamic, Eigen::Dynamic > & | *m*, |
|  |  | size\_t | *j* |
|  | ) |  |  |

inline

Return the specified column of the specified matrix using start-at-1 indexing.

This is equivalent to calling `m.col(i - 1)` and assigning the resulting template expression to a column vector.

Parameters
:   |  |  |
    | --- | --- |
    | m | Matrix. |
    | j | Column index (count from 1). |

Returns
:   Specified column of the matrix.

Definition at line 24 of file col.hpp.

template<typename T , int R, int C>

|  |  |  |  |  |  |
| --- | --- | --- | --- | --- | --- |
| size\_t stan::math::cols | ( | const Eigen::Matrix< T, R, C > & | *m* | ) |  |

inline

Definition at line 12 of file cols.hpp.

template<int R1, int C1, int R2, int C2>

|  |  |  |  |
| --- | --- | --- | --- |
| Eigen::Matrix<double, 1, C1> stan::math::columns\_dot\_product | ( | const Eigen::Matrix< double, R1, C1 > & | *v1*, |
|  |  | const Eigen::Matrix< double, R2, C2 > & | *v2* |
|  | ) |  |  |

inline

Returns the dot product of the specified vectors.

Parameters
:   |  |  |
    | --- | --- |
    | v1 | First vector. |
    | v2 | Second vector. |

Returns
:   Dot product of the vectors.

Exceptions
:   |  |  |
    | --- | --- |
    | std::domain\_error | If the vectors are not the same size or if they are both not vector dimensioned. |

Definition at line 22 of file columns\_dot\_product.hpp.

template<typename T , int R, int C>

|  |  |  |  |  |  |
| --- | --- | --- | --- | --- | --- |
| Eigen::Matrix<T,1,C> stan::math::columns\_dot\_self | ( | const Eigen::Matrix< T, R, C > & | *x* | ) |  |

inline

Returns the dot product of each column of a matrix with itself.

Parameters
:   |  |  |
    | --- | --- |
    | x | Matrix. |

Template Parameters
:   |  |  |
    | --- | --- |
    | T | scalar type |

Definition at line 16 of file columns\_dot\_self.hpp.

|  |  |  |  |  |  |
| --- | --- | --- | --- | --- | --- |
| matrix\_d stan::math::crossprod | ( | const matrix\_d & | *M* | ) |  |

inline

Returns the result of pre-multiplying a matrix by its own transpose.

Parameters
:   |  |  |
    | --- | --- |
    | M | Matrix to multiply. |

Returns
:   Transpose of M times M

Definition at line 17 of file crossprod.hpp.

template<typename T >

|  |  |  |  |  |  |
| --- | --- | --- | --- | --- | --- |
| std::vector<T> stan::math::cumulative\_sum | ( | const std::vector< T > & | *x* | ) |  |

inline

Return the cumulative sum of the specified vector.

The cumulative sum of a vector of values

is the

@code x[0], x[1] + x[2], ..., x[1] + ,..., + x[x.size()-1]

Template Parameters
:   |  |  |
    | --- | --- |
    | T | Scalar type of vector. |

Parameters
:   |  |  |
    | --- | --- |
    | x | Vector of values. |

Returns
:   Cumulative sum of values.

Definition at line 23 of file cumulative\_sum.hpp.

template<typename T , int R, int C>

|  |  |  |  |  |  |
| --- | --- | --- | --- | --- | --- |
| Eigen::Matrix<T,R,C> stan::math::cumulative\_sum | ( | const Eigen::Matrix< T, R, C > & | *m* | ) |  |

inline

Return the cumulative sum of the specified matrix.

The cumulative sum is of the same type as the input and has values defined by

x(0), x(1) + x(2), ..., x(1) + ,..., + x(x.size()-1)

Template Parameters
:   |  |  |
    | --- | --- |
    | T | Scalar type of matrix. |
    | R | Row type of matrix. |
    | C | Column type of matrix. |

Parameters
:   |  |  |
    | --- | --- |
    | m | Matrix of values. |

Returns
:   Cumulative sum of values.

Definition at line 49 of file cumulative\_sum.hpp.

template<typename T , int R, int C>

|  |  |  |  |  |  |
| --- | --- | --- | --- | --- | --- |
| T stan::math::determinant | ( | const Eigen::Matrix< T, R, C > & | *m* | ) |  |

inline

Returns the determinant of the specified square matrix.

Parameters
:   |  |  |
    | --- | --- |
    | m | Specified matrix. |

Returns
:   Determinant of the matrix.

Exceptions
:   |  |  |
    | --- | --- |
    | std::domain\_error | if matrix is not square. |

Definition at line 18 of file determinant.hpp.

template<typename T >

|  |  |  |  |  |  |
| --- | --- | --- | --- | --- | --- |
| Eigen::Matrix<T,Eigen::Dynamic,Eigen::Dynamic> stan::math::diag\_matrix | ( | const Eigen::Matrix< T, Eigen::Dynamic, 1 > & | *v* | ) |  |

inline

Return a square diagonal matrix with the specified vector of coefficients as the diagonal values.

Parameters
:   |  |  |  |
    | --- | --- | --- |
    | [in] | v | Specified vector. |

Returns
:   Diagonal matrix with vector as diagonal values.

Definition at line 18 of file diag\_matrix.hpp.

template<typename T1 , typename T2 , int R1, int C1, int R2, int C2>

|  |  |  |  |
| --- | --- | --- | --- |
| Eigen::Matrix<typename boost::math::tools::promote\_args<T1,T2>::type, R1, C1> stan::math::diag\_post\_multiply | ( | const Eigen::Matrix< T1, R1, C1 > & | *m1*, |
|  |  | const Eigen::Matrix< T2, R2, C2 > & | *m2* |
|  | ) |  |  |

Definition at line 13 of file diag\_post\_multiply.hpp.

template<typename T1 , typename T2 , int R1, int C1, int R2, int C2>

|  |  |  |  |
| --- | --- | --- | --- |
| Eigen::Matrix<typename boost::math::tools::promote\_args<T1,T2>::type, R2, C2> stan::math::diag\_pre\_multiply | ( | const Eigen::Matrix< T1, R1, C1 > & | *m1*, |
|  |  | const Eigen::Matrix< T2, R2, C2 > & | *m2* |
|  | ) |  |  |

Definition at line 13 of file diag\_pre\_multiply.hpp.

template<typename T >

|  |  |  |  |  |  |
| --- | --- | --- | --- | --- | --- |
| Eigen::Matrix<T,Eigen::Dynamic,1> stan::math::diagonal | ( | const Eigen::Matrix< T, Eigen::Dynamic, Eigen::Dynamic > & | *m* | ) |  |

inline

Return a column vector of the diagonal elements of the specified matrix.

The matrix is not required to be square.

Parameters
:   |  |  |
    | --- | --- |
    | m | Specified matrix. |

Returns
:   Diagonal of the matrix.

Definition at line 18 of file diagonal.hpp.

|  |  |  |  |  |  |
| --- | --- | --- | --- | --- | --- |
| double stan::math::digamma | ( | double | *x* | ) |  |

Definition at line 39 of file digamma.hpp.

template<typename T >

|  |  |  |  |
| --- | --- | --- | --- |
| void stan::math::dims | ( | const T & | *x*, |
|  |  | std::vector< int > & | *result* |
|  | ) |  |  |

inline

Definition at line 13 of file dims.hpp.

template<typename T , int R, int C>

|  |  |  |  |
| --- | --- | --- | --- |
| void stan::math::dims | ( | const Eigen::Matrix< T, R, C > & | *x*, |
|  |  | std::vector< int > & | *result* |
|  | ) |  |  |

inline

Definition at line 18 of file dims.hpp.

template<typename T >

|  |  |  |  |
| --- | --- | --- | --- |
| void stan::math::dims | ( | const std::vector< T > & | *x*, |
|  |  | std::vector< int > & | *result* |
|  | ) |  |  |

inline

Definition at line 25 of file dims.hpp.

template<typename T >

|  |  |  |  |  |  |
| --- | --- | --- | --- | --- | --- |
| std::vector<int> stan::math::dims | ( | const T & | *x* | ) |  |

inline

Definition at line 34 of file dims.hpp.

|  |  |  |  |
| --- | --- | --- | --- |
| double stan::math::dist | ( | const std::vector< double > & | *x*, |
|  |  | const std::vector< double > & | *y* |
|  | ) |  |  |

inline

Definition at line 11 of file dist.hpp.

template<typename T1 , int R1, int C1, typename T2 , int R2, int C2>

|  |  |  |  |
| --- | --- | --- | --- |
| boost::math::tools::promote\_args<T1,T2>::type stan::math::distance | ( | const Eigen::Matrix< T1, R1, C1 > & | *v1*, |
|  |  | const Eigen::Matrix< T2, R2, C2 > & | *v2* |
|  | ) |  |  |

inline

Returns the distance between the specified vectors.

Parameters
:   |  |  |
    | --- | --- |
    | v1 | First vector. |
    | v2 | Second vector. |

Returns
:   Dot product of the vectors.

Exceptions
:   |  |  |
    | --- | --- |
    | std::domain\_error | If the vectors are not the same size or if they are both not vector dimensioned. |

Definition at line 25 of file distance.hpp.

|  |  |  |  |
| --- | --- | --- | --- |
| int stan::math::divide | ( | const int | *x*, |
|  |  | const int | *y* |
|  | ) |  |  |

inline

Definition at line 11 of file divide.hpp.

template<int R, int C, typename T >

|  |  |  |  |
| --- | --- | --- | --- |
| boost::enable\_if\_c<boost::is\_arithmetic<T>::value, Eigen::Matrix<double, R, C> >::type stan::math::divide | ( | const Eigen::Matrix< double, R, C > & | *m*, |
|  |  | T | *c* |
|  | ) |  |  |

inline

Return specified matrix divided by specified scalar.

Template Parameters
:   |  |  |
    | --- | --- |
    | R | Row type for matrix. |
    | C | Column type for matrix. |

Parameters
:   |  |  |
    | --- | --- |
    | m | Matrix. |
    | c | Scalar. |

Returns
:   Matrix divided by scalar.

Definition at line 23 of file divide.hpp.

template<typename T , typename T\_result , typename T\_msg >

|  |  |  |  |
| --- | --- | --- | --- |
| bool stan::math::dom\_err | ( | const char \* | *function*, |
|  |  | const T & | *y*, |
|  |  | const char \* | *name*, |
|  |  | const char \* | *error\_msg*, |
|  |  | const T\_msg | *error\_msg2*, |
|  |  | T\_result \* | *result* |
|  | ) |  |  |

inline

Definition at line 36 of file dom\_err.hpp.

template<typename T , typename T\_result , typename T\_msg >

|  |  |  |  |
| --- | --- | --- | --- |
| bool stan::math::dom\_err\_vec | ( | const size\_t | *i*, |
|  |  | const char \* | *function*, |
|  |  | const T & | *y*, |
|  |  | const char \* | *name*, |
|  |  | const char \* | *error\_msg*, |
|  |  | const T\_msg | *error\_msg2*, |
|  |  | T\_result \* | *result* |
|  | ) |  |  |

inline

Definition at line 33 of file dom\_err\_vec.hpp.

|  |  |  |  |
| --- | --- | --- | --- |
| double stan::math::dot | ( | const std::vector< double > & | *x*, |
|  |  | const std::vector< double > & | *y* |
|  | ) |  |  |

inline

Definition at line 11 of file dot.hpp.

template<int R1, int C1, int R2, int C2>

|  |  |  |  |
| --- | --- | --- | --- |
| double stan::math::dot\_product | ( | const Eigen::Matrix< double, R1, C1 > & | *v1*, |
|  |  | const Eigen::Matrix< double, R2, C2 > & | *v2* |
|  | ) |  |  |

inline

Returns the dot product of the specified vectors.

Parameters
:   |  |  |
    | --- | --- |
    | v1 | First vector. |
    | v2 | Second vector. |

Returns
:   Dot product of the vectors.

Exceptions
:   |  |  |
    | --- | --- |
    | std::domain\_error | If the vectors are not the same size or if they are both not vector dimensioned. |

Definition at line 22 of file dot\_product.hpp.

|  |  |  |  |
| --- | --- | --- | --- |
| double stan::math::dot\_product | ( | const double \* | *v1*, |
|  |  | const double \* | *v2*, |
|  |  | size\_t | *length* |
|  | ) |  |  |

inline

Returns the dot product of the specified arrays of doubles.

Parameters
:   |  |  |
    | --- | --- |
    | v1 | First array. |
    | v2 | Second array. |
    | length | Length of both arrays. |

Definition at line 36 of file dot\_product.hpp.

|  |  |  |  |
| --- | --- | --- | --- |
| double stan::math::dot\_product | ( | const std::vector< double > & | *v1*, |
|  |  | const std::vector< double > & | *v2* |
|  | ) |  |  |

inline

Returns the dot product of the specified arrays of doubles.

Parameters
:   |  |  |
    | --- | --- |
    | v1 | First array. |
    | v2 | Second array. |

Exceptions
:   |  |  |
    | --- | --- |
    | std::domain\_error | if the vectors are not the same size. |

Definition at line 49 of file dot\_product.hpp.

|  |  |  |  |  |  |
| --- | --- | --- | --- | --- | --- |
| double stan::math::dot\_self | ( | const std::vector< double > & | *x* | ) |  |

inline

Definition at line 11 of file dot\_self.hpp.

template<int R, int C>

|  |  |  |  |  |  |
| --- | --- | --- | --- | --- | --- |
| double stan::math::dot\_self | ( | const Eigen::Matrix< double, R, C > & | *v* | ) |  |

inline

Returns the dot product of the specified vector with itself.

Parameters
:   |  |  |
    | --- | --- |
    | v | Vector. |

Template Parameters
:   |  |  |
    | --- | --- |
    | R | number of rows or `Eigen::Dynamic` for dynamic |
    | C | number of rows or `Eigen::Dyanmic` for dynamic |

Exceptions
:   |  |  |
    | --- | --- |
    | std::domain\_error | If v is not vector dimensioned. |

Definition at line 18 of file dot\_self.hpp.

|  |  |  |  |  |
| --- | --- | --- | --- | --- |
| double stan::math::e | ( |  | ) |  |

inline

Return the base of the natural logarithm.

Returns
:   Base of natural logarithm.

Definition at line 86 of file constants.hpp.

template<typename T >

|  |  |  |  |  |  |
| --- | --- | --- | --- | --- | --- |
| Eigen::Matrix<T,Eigen::Dynamic,1> stan::math::eigenvalues\_sym | ( | const Eigen::Matrix< T, Eigen::Dynamic, Eigen::Dynamic > & | *m* | ) |  |

Return the eigenvalues of the specified symmetric matrix in descending order of magnitude.

This function is more efficient than the general eigenvalues function for symmetric matrices.

See `eigen_decompose()` for more information.

Parameters
:   |  |  |
    | --- | --- |
    | m | Specified matrix. |

Returns
:   Eigenvalues of matrix.

Definition at line 22 of file eigenvalues\_sym.hpp.

template<typename T >

|  |  |  |  |  |  |
| --- | --- | --- | --- | --- | --- |
| Eigen::Matrix<T,Eigen::Dynamic,Eigen::Dynamic> stan::math::eigenvectors\_sym | ( | const Eigen::Matrix< T, Eigen::Dynamic, Eigen::Dynamic > & | *m* | ) |  |

Definition at line 13 of file eigenvectors\_sym.hpp.

template<typename T1 , typename T2 , int R, int C>

|  |  |  |  |
| --- | --- | --- | --- |
| Eigen::Matrix<typename boost::math::tools::promote\_args<T1,T2>::type, R, C> stan::math::elt\_divide | ( | const Eigen::Matrix< T1, R, C > & | *m1*, |
|  |  | const Eigen::Matrix< T2, R, C > & | *m2* |
|  | ) |  |  |

Return the elementwise division of the specified matrices.

Template Parameters
:   |  |  |
    | --- | --- |
    | T1 | Type of scalars in first matrix. |
    | T2 | Type of scalars in second matrix. |
    | R | Row type of both matrices. |
    | C | Column type of both matrices. |

Parameters
:   |  |  |
    | --- | --- |
    | m1 | First matrix |
    | m2 | Second matrix |

Returns
:   Elementwise division of matrices.

Definition at line 24 of file elt\_divide.hpp.

template<typename T1 , typename T2 , int R, int C>

|  |  |  |  |
| --- | --- | --- | --- |
| Eigen::Matrix<typename boost::math::tools::promote\_args<T1,T2>::type, R, C> stan::math::elt\_divide | ( | const Eigen::Matrix< T1, R, C > & | *m*, |
|  |  | T2 | *s* |
|  | ) |  |  |

Return the elementwise division of the specified matrix by the specified scalar.

Template Parameters
:   |  |  |
    | --- | --- |
    | T1 | Type of scalars in the matrix. |
    | T2 | Type of the scalar. |
    | R | Row type of the matrix. |
    | C | Column type of the matrix. |

Parameters
:   |  |  |
    | --- | --- |
    | m | matrix |
    | s | scalar |

Returns
:   Elementwise division of a scalar by matrix.

Definition at line 49 of file elt\_divide.hpp.

template<typename T1 , typename T2 , int R, int C>

|  |  |  |  |
| --- | --- | --- | --- |
| Eigen::Matrix<typename boost::math::tools::promote\_args<T1,T2>::type, R, C> stan::math::elt\_divide | ( | T1 | *s*, |
|  |  | const Eigen::Matrix< T2, R, C > & | *m* |
|  | ) |  |  |

Return the elementwise division of the specified scalar by the specified matrix.

Template Parameters
:   |  |  |
    | --- | --- |
    | T1 | Type of the scalar. |
    | T2 | Type of scalars in the matrix. |
    | R | Row type of the matrix. |
    | C | Column type of the matrix. |

Parameters
:   |  |  |
    | --- | --- |
    | s | scalar |
    | m | matrix |

Returns
:   Elementwise division of a scalar by matrix.

Definition at line 67 of file elt\_divide.hpp.

template<typename T1 , typename T2 , int R, int C>

|  |  |  |  |
| --- | --- | --- | --- |
| Eigen::Matrix<typename boost::math::tools::promote\_args<T1,T2>::type, R, C> stan::math::elt\_multiply | ( | const Eigen::Matrix< T1, R, C > & | *m1*, |
|  |  | const Eigen::Matrix< T2, R, C > & | *m2* |
|  | ) |  |  |

Return the elementwise multiplication of the specified matrices.

Template Parameters
:   |  |  |
    | --- | --- |
    | T1 | Type of scalars in first matrix. |
    | T2 | Type of scalars in second matrix. |
    | R | Row type of both matrices. |
    | C | Column type of both matrices. |

Parameters
:   |  |  |
    | --- | --- |
    | m1 | First matrix |
    | m2 | Second matrix |

Returns
:   Elementwise product of matrices.

Definition at line 25 of file elt\_multiply.hpp.

template<typename T , int Rows, int Cols>

|  |  |  |  |  |  |
| --- | --- | --- | --- | --- | --- |
| Eigen::Matrix<T,Rows,Cols> stan::math::exp | ( | const Eigen::Matrix< T, Rows, Cols > & | *m* | ) |  |

inline

Return the element-wise exponentiation of the matrix or vector.

Parameters
:   |  |  |
    | --- | --- |
    | m | The matrix or vector. |

Returns
:   ret(i,j) = exp(m(i,j))

Definition at line 18 of file exp.hpp.

template<int Rows, int Cols>

|  |  |  |  |  |  |
| --- | --- | --- | --- | --- | --- |
| Eigen::Matrix<double,Rows,Cols> stan::math::exp | ( | const Eigen::Matrix< double, Rows, Cols > & | *m* | ) |  |

inline

Definition at line 26 of file exp.hpp.

template<typename T >

|  |  |  |  |  |  |
| --- | --- | --- | --- | --- | --- |
| boost::math::tools::promote\_args<T>::type stan::math::exp2 | ( | const T | *y* | ) |  |

inline

Return the exponent base 2 of the specified argument (C99).

The exponent base 2 function is defined by

`exp2(y) = pow(2.0,y)`.

Parameters
:   |  |  |
    | --- | --- |
    | y | Value. |

Template Parameters
:   |  |  |
    | --- | --- |
    | T | Type of scalar. |

Returns
:   Exponent base 2 of value.

Definition at line 23 of file exp2.hpp.

|  |  |  |  |
| --- | --- | --- | --- |
| double stan::math::F32 | ( | double | *a*, |
|  |  | double | *b*, |
|  |  | double | *c*, |
|  |  | double | *d*, |
|  |  | double | *e*, |
|  |  | double | *z*, |
|  |  | double | *precision* = `1e-6` |
|  | ) |  |  |

Definition at line 14 of file internal\_math.hpp.

template<typename T1 , typename T2 >

|  |  |  |  |
| --- | --- | --- | --- |
| boost::math::tools::promote\_args<T1,T2>::type stan::math::falling\_factorial | ( | const T1 | *x*, |
|  |  | const T2 | *n* |
|  | ) |  |  |

inline

Definition at line 53 of file falling\_factorial.hpp.

template<typename T1 , typename T2 >

|  |  |  |  |
| --- | --- | --- | --- |
| boost::math::tools::promote\_args<T1, T2>::type stan::math::fdim | ( | T1 | *a*, |
|  |  | T2 | *b* |
|  | ) |  |  |

inline

The positive difference function (C99).

The function is defined by

`fdim(a,b) = (a > b) ? (a - b) : 0.0`.

Parameters
:   |  |  |
    | --- | --- |
    | a | First value. |
    | b | Second value. |

Returns
:   Returns min(a - b, 0.0).

Definition at line 23 of file fdim.hpp.

template<typename T , typename S >

|  |  |  |  |
| --- | --- | --- | --- |
| void stan::math::fill | ( | T & | *x*, |
|  |  | const S & | *y* |
|  | ) |  |  |

Fill the specified container with the specified value.

This base case simply assigns the value to the container.

Template Parameters
:   |  |  |
    | --- | --- |
    | T | Type of reference container. |
    | S | Type of value. |

Parameters
:   |  |  |
    | --- | --- |
    | x | Container. |
    | y | Value. |

Definition at line 22 of file fill.hpp.

template<typename T , int R, int C, typename S >

|  |  |  |  |
| --- | --- | --- | --- |
| void stan::math::fill | ( | Eigen::Matrix< T, R, C > & | *x*, |
|  |  | const S & | *y* |
|  | ) |  |  |

Fill the specified container with the specified value.

The specified matrix is filled by element.

Template Parameters
:   |  |  |
    | --- | --- |
    | T | Type of scalar for matrix container. |
    | R | Row type of matrix. |
    | C | Column type of matrix. |
    | S | Type of value. |

Parameters
:   |  |  |
    | --- | --- |
    | x | Container. |
    | y | Value. |

Definition at line 39 of file fill.hpp.

template<typename T , typename S >

|  |  |  |  |
| --- | --- | --- | --- |
| void stan::math::fill | ( | std::vector< T > & | *x*, |
|  |  | const S & | *y* |
|  | ) |  |  |

Fill the specified container with the specified value.

Each container in the specified standard vector is filled recursively by calling `fill`.

Template Parameters
:   |  |  |
    | --- | --- |
    | T | Type of container in vector. |
    | S | Type of value. |

Parameters
:   |  |  |
    | --- | --- |
    | x | Container. |
    | y | Value. |

Definition at line 55 of file fill.hpp.

|  |  |  |  |
| --- | --- | --- | --- |
| double stan::math::gamma\_p | ( | double | *x*, |
|  |  | double | *a* |
|  | ) |  |  |

Definition at line 53 of file gamma\_p.hpp.

|  |  |  |  |
| --- | --- | --- | --- |
| double stan::math::gamma\_q | ( | double | *x*, |
|  |  | double | *a* |
|  | ) |  |  |

Definition at line 53 of file gamma\_q.hpp.

template<typename T >

|  |  |  |  |
| --- | --- | --- | --- |
| const T& stan::math::get\_base1 | ( | const std::vector< T > & | *x*, |
|  |  | size\_t | *i*, |
|  |  | const char \* | *error\_msg*, |
|  |  | size\_t | *idx* |
|  | ) |  |  |

inline

Return a reference to the value of the specified vector at the specified base-one index.

If the index is out of range, throw a `std::out_of_range` exception with the specified error message and index indicated.

Parameters
:   |  |  |
    | --- | --- |
    | x | Vector from which to get a value. |
    | i | Index into vector plus 1. |
    | error\_msg | Error message if the index is out of range. |
    | idx | Nested index level to report in error message if the index is out of range. |

Returns
:   Value of vector at `i - 1`

Template Parameters
:   |  |  |
    | --- | --- |
    | T | type of value. |

Definition at line 27 of file get\_base1.hpp.

template<typename T >

|  |  |  |  |
| --- | --- | --- | --- |
| const T& stan::math::get\_base1 | ( | const std::vector< std::vector< T > > & | *x*, |
|  |  | size\_t | *i1*, |
|  |  | size\_t | *i2*, |
|  |  | const char \* | *error\_msg*, |
|  |  | size\_t | *idx* |
|  | ) |  |  |

inline

Return a reference to the value of the specified vector at the specified base-one indexes.

If an index is out of range, throw a `std::out_of_range` exception with the specified error message and index indicated.

Parameters
:   |  |  |
    | --- | --- |
    | x | Vector from which to get a value. |
    | i1 | First index plus 1. |
    | i2 | Second index plus 1. |
    | error\_msg | Error message if an index is out of range. |
    | idx | Nested index level to report in error message if the index is out of range. |

Returns
:   Value of vector at indexes.

Template Parameters
:   |  |  |
    | --- | --- |
    | T | type of value. |

Definition at line 52 of file get\_base1.hpp.

template<typename T >

|  |  |  |  |
| --- | --- | --- | --- |
| const T& stan::math::get\_base1 | ( | const std::vector< std::vector< std::vector< T > > > & | *x*, |
|  |  | size\_t | *i1*, |
|  |  | size\_t | *i2*, |
|  |  | size\_t | *i3*, |
|  |  | const char \* | *error\_msg*, |
|  |  | size\_t | *idx* |
|  | ) |  |  |

inline

Return a reference to the value of the specified vector at the specified base-one indexes.

If an index is out of range, throw a `std::out_of_range` exception with the specified error message and index indicated.

Parameters
:   |  |  |
    | --- | --- |
    | x | Vector from which to get a value. |
    | i1 | First index plus 1. |
    | i2 | Second index plus 1. |
    | i3 | Third index plus 1. |
    | error\_msg | Error message if an index is out of range. |
    | idx | Nested index level to report in error message if the index is out of range. |

Returns
:   Value of vector at indexes.

Template Parameters
:   |  |  |
    | --- | --- |
    | T | type of value. |

Definition at line 79 of file get\_base1.hpp.

template<typename T >

|  |  |  |  |
| --- | --- | --- | --- |
| const T& stan::math::get\_base1 | ( | const std::vector< std::vector< std::vector< std::vector< T > > > > & | *x*, |
|  |  | size\_t | *i1*, |
|  |  | size\_t | *i2*, |
|  |  | size\_t | *i3*, |
|  |  | size\_t | *i4*, |
|  |  | const char \* | *error\_msg*, |
|  |  | size\_t | *idx* |
|  | ) |  |  |

inline

Return a reference to the value of the specified vector at the specified base-one indexes.

If an index is out of range, throw a `std::out_of_range` exception with the specified error message and index indicated.

Parameters
:   |  |  |
    | --- | --- |
    | x | Vector from which to get a value. |
    | i1 | First index plus 1. |
    | i2 | Second index plus 1. |
    | i3 | Third index plus 1. |
    | i4 | Fourth index plus 1. |
    | error\_msg | Error message if an index is out of range. |
    | idx | Nested index level to report in error message if the index is out of range. |

Returns
:   Value of vector at indexes.

Template Parameters
:   |  |  |
    | --- | --- |
    | T | type of value. |

Definition at line 108 of file get\_base1.hpp.

template<typename T >

|  |  |  |  |
| --- | --- | --- | --- |
| const T& stan::math::get\_base1 | ( | const std::vector< std::vector< std::vector< std::vector< std::vector< T > > > > > & | *x*, |
|  |  | size\_t | *i1*, |
|  |  | size\_t | *i2*, |
|  |  | size\_t | *i3*, |
|  |  | size\_t | *i4*, |
|  |  | size\_t | *i5*, |
|  |  | const char \* | *error\_msg*, |
|  |  | size\_t | *idx* |
|  | ) |  |  |

inline

Return a reference to the value of the specified vector at the specified base-one indexes.

If an index is out of range, throw a `std::out_of_range` exception with the specified error message and index indicated.

Parameters
:   |  |  |
    | --- | --- |
    | x | Vector from which to get a value. |
    | i1 | First index plus 1. |
    | i2 | Second index plus 1. |
    | i3 | Third index plus 1. |
    | i4 | Fourth index plus 1. |
    | i5 | Fifth index plus 1. |
    | error\_msg | Error message if an index is out of range. |
    | idx | Nested index level to report in error message if the index is out of range. |

Returns
:   Value of vector at indexes.

Template Parameters
:   |  |  |
    | --- | --- |
    | T | type of value. |

Definition at line 139 of file get\_base1.hpp.

template<typename T >

|  |  |  |  |
| --- | --- | --- | --- |
| const T& stan::math::get\_base1 | ( | const std::vector< std::vector< std::vector< std::vector< std::vector< std::vector< T > > > > > > & | *x*, |
|  |  | size\_t | *i1*, |
|  |  | size\_t | *i2*, |
|  |  | size\_t | *i3*, |
|  |  | size\_t | *i4*, |
|  |  | size\_t | *i5*, |
|  |  | size\_t | *i6*, |
|  |  | const char \* | *error\_msg*, |
|  |  | size\_t | *idx* |
|  | ) |  |  |

inline

Return a reference to the value of the specified vector at the specified base-one indexes.

If an index is out of range, throw a `std::out_of_range` exception with the specified error message and index indicated.

Parameters
:   |  |  |
    | --- | --- |
    | x | Vector from which to get a value. |
    | i1 | First index plus 1. |
    | i2 | Second index plus 1. |
    | i3 | Third index plus 1. |
    | i4 | Fourth index plus 1. |
    | i5 | Fifth index plus 1. |
    | i6 | Sixth index plus 1. |
    | error\_msg | Error message if an index is out of range. |
    | idx | Nested index level to report in error message if the index is out of range. |

Returns
:   Value of vector at indexes.

Template Parameters
:   |  |  |
    | --- | --- |
    | T | type of value. |

Definition at line 172 of file get\_base1.hpp.

template<typename T >

|  |  |  |  |
| --- | --- | --- | --- |
| const T& stan::math::get\_base1 | ( | const std::vector< std::vector< std::vector< std::vector< std::vector< std::vector< std::vector< T > > > > > > > & | *x*, |
|  |  | size\_t | *i1*, |
|  |  | size\_t | *i2*, |
|  |  | size\_t | *i3*, |
|  |  | size\_t | *i4*, |
|  |  | size\_t | *i5*, |
|  |  | size\_t | *i6*, |
|  |  | size\_t | *i7*, |
|  |  | const char \* | *error\_msg*, |
|  |  | size\_t | *idx* |
|  | ) |  |  |

inline

Return a reference to the value of the specified vector at the specified base-one indexes.

If an index is out of range, throw a `std::out_of_range` exception with the specified error message and index indicated.

Parameters
:   |  |  |
    | --- | --- |
    | x | Vector from which to get a value. |
    | i1 | First index plus 1. |
    | i2 | Second index plus 1. |
    | i3 | Third index plus 1. |
    | i4 | Fourth index plus 1. |
    | i5 | Fifth index plus 1. |
    | i6 | Sixth index plus 1. |
    | i7 | Seventh index plus 1. |
    | error\_msg | Error message if an index is out of range. |
    | idx | Nested index level to report in error message if the index is out of range. |

Returns
:   Value of vector at indexes.

Template Parameters
:   |  |  |
    | --- | --- |
    | T | type of value. |

Definition at line 208 of file get\_base1.hpp.

template<typename T >

|  |  |  |  |
| --- | --- | --- | --- |
| const T& stan::math::get\_base1 | ( | const std::vector< std::vector< std::vector< std::vector< std::vector< std::vector< std::vector< std::vector< T > > > > > > > > & | *x*, |
|  |  | size\_t | *i1*, |
|  |  | size\_t | *i2*, |
|  |  | size\_t | *i3*, |
|  |  | size\_t | *i4*, |
|  |  | size\_t | *i5*, |
|  |  | size\_t | *i6*, |
|  |  | size\_t | *i7*, |
|  |  | size\_t | *i8*, |
|  |  | const char \* | *error\_msg*, |
|  |  | size\_t | *idx* |
|  | ) |  |  |

inline

Return a reference to the value of the specified vector at the specified base-one indexes.

If an index is out of range, throw a `std::out_of_range` exception with the specified error message and index indicated.

Parameters
:   |  |  |
    | --- | --- |
    | x | Vector from which to get a value. |
    | i1 | First index plus 1. |
    | i2 | Second index plus 1. |
    | i3 | Third index plus 1. |
    | i4 | Fourth index plus 1. |
    | i5 | Fifth index plus 1. |
    | i6 | Sixth index plus 1. |
    | i7 | Seventh index plus 1. |
    | i8 | Eigth index plus 1. |
    | error\_msg | Error message if an index is out of range. |
    | idx | Nested index level to report in error message if the index is out of range. |

Returns
:   Value of vector at indexes.

Template Parameters
:   |  |  |
    | --- | --- |
    | T | type of value. |

Definition at line 246 of file get\_base1.hpp.

template<typename T >

|  |  |  |  |
| --- | --- | --- | --- |
| Eigen::Matrix<T,1,Eigen::Dynamic> stan::math::get\_base1 | ( | const Eigen::Matrix< T, Eigen::Dynamic, Eigen::Dynamic > & | *x*, |
|  |  | size\_t | *m*, |
|  |  | const char \* | *error\_msg*, |
|  |  | size\_t | *idx* |
|  | ) |  |  |

inline

Return a copy of the row of the specified vector at the specified base-one row index.

If the index is out of range, throw a `std::out_of_range` exception with the specified error message and index indicated.

**Warning**: Because a copy is involved, it is inefficient to access element of matrices by first using this method to get a row then using a second call to get the value at a specified column.

Parameters
:   |  |  |
    | --- | --- |
    | x | Matrix from which to get a row |
    | m | Index into matrix plus 1. |
    | error\_msg | Error message if the index is out of range. |
    | idx | Nested index level to report in error message if the index is out of range. |

Returns
:   Row of matrix at `i - 1`.

Template Parameters
:   |  |  |
    | --- | --- |
    | T | type of value. |

Definition at line 284 of file get\_base1.hpp.

template<typename T >

|  |  |  |  |
| --- | --- | --- | --- |
| const T& stan::math::get\_base1 | ( | const Eigen::Matrix< T, Eigen::Dynamic, Eigen::Dynamic > & | *x*, |
|  |  | size\_t | *m*, |
|  |  | size\_t | *n*, |
|  |  | const char \* | *error\_msg*, |
|  |  | size\_t | *idx* |
|  | ) |  |  |

inline

Return a reference to the value of the specified matrix at the specified base-one row and column indexes.

If either index is out of range, throw a `std::out_of_range` exception with the specified error message and index indicated.

Parameters
:   |  |  |
    | --- | --- |
    | x | Matrix from which to get a row |
    | m | Row index plus 1. |
    | n | Column index plus 1. |
    | error\_msg | Error message if either index is out of range. |
    | idx | Nested index level to report in error message if either index is out of range. |

Returns
:   Value of matrix at row `m - 1` and column `n - 1`.

Template Parameters
:   |  |  |
    | --- | --- |
    | T | type of value. |

Definition at line 310 of file get\_base1.hpp.

template<typename T >

|  |  |  |  |
| --- | --- | --- | --- |
| const T& stan::math::get\_base1 | ( | const Eigen::Matrix< T, Eigen::Dynamic, 1 > & | *x*, |
|  |  | size\_t | *m*, |
|  |  | const char \* | *error\_msg*, |
|  |  | size\_t | *idx* |
|  | ) |  |  |

inline

Return a reference to the value of the specified column vector at the specified base-one index.

If the index is out of range, throw a `std::out_of_range` exception with the specified error message and index indicated.

Parameters
:   |  |  |
    | --- | --- |
    | x | Column vector from which to get a value. |
    | m | Row index plus 1. |
    | error\_msg | Error message if the index is out of range. |
    | idx | Nested index level to report in error message if the index is out of range. |

Returns
:   Value of column vector at row `m - 1`.

Template Parameters
:   |  |  |
    | --- | --- |
    | T | type of value. |

Definition at line 336 of file get\_base1.hpp.

template<typename T >

|  |  |  |  |
| --- | --- | --- | --- |
| const T& stan::math::get\_base1 | ( | const Eigen::Matrix< T, 1, Eigen::Dynamic > & | *x*, |
|  |  | size\_t | *n*, |
|  |  | const char \* | *error\_msg*, |
|  |  | size\_t | *idx* |
|  | ) |  |  |

inline

Return a reference to the value of the specified row vector at the specified base-one index.

If the index is out of range, throw a `std::out_of_range` exception with the specified error message and index indicated.

Parameters
:   |  |  |
    | --- | --- |
    | x | Row vector from which to get a value. |
    | n | Column index plus 1. |
    | error\_msg | Error message if the index is out of range. |
    | idx | Nested index level to report in error message if the index is out of range. |

Returns
:   Value of row vector at column `n - 1`.

Template Parameters
:   |  |  |
    | --- | --- |
    | T | type of value. |

Definition at line 360 of file get\_base1.hpp.

template<typename T >

|  |  |  |  |
| --- | --- | --- | --- |
| T& stan::math::get\_base1\_lhs | ( | std::vector< T > & | *x*, |
|  |  | size\_t | *i*, |
|  |  | const char \* | *error\_msg*, |
|  |  | size\_t | *idx* |
|  | ) |  |  |

inline

Return a reference to the value of the specified vector at the specified base-one index.

If the index is out of range, throw a `std::out_of_range` exception with the specified error message and index indicated.

Parameters
:   |  |  |
    | --- | --- |
    | x | Vector from which to get a value. |
    | i | Index into vector plus 1. |
    | error\_msg | Error message if the index is out of range. |
    | idx | Nested index level to report in error message if the index is out of range. |

Returns
:   Value of vector at `i - 1`

Template Parameters
:   |  |  |
    | --- | --- |
    | T | type of value. |

Definition at line 27 of file get\_base1\_lhs.hpp.

template<typename T >

|  |  |  |  |
| --- | --- | --- | --- |
| T& stan::math::get\_base1\_lhs | ( | std::vector< std::vector< T > > & | *x*, |
|  |  | size\_t | *i1*, |
|  |  | size\_t | *i2*, |
|  |  | const char \* | *error\_msg*, |
|  |  | size\_t | *idx* |
|  | ) |  |  |

inline

Return a reference to the value of the specified vector at the specified base-one indexes.

If an index is out of range, throw a `std::out_of_range` exception with the specified error message and index indicated.

Parameters
:   |  |  |
    | --- | --- |
    | x | Vector from which to get a value. |
    | i1 | First index plus 1. |
    | i2 | Second index plus 1. |
    | error\_msg | Error message if an index is out of range. |
    | idx | Nested index level to report in error message if the index is out of range. |

Returns
:   Value of vector at indexes.

Template Parameters
:   |  |  |
    | --- | --- |
    | T | type of value. |

Definition at line 52 of file get\_base1\_lhs.hpp.

template<typename T >

|  |  |  |  |
| --- | --- | --- | --- |
| T& stan::math::get\_base1\_lhs | ( | std::vector< std::vector< std::vector< T > > > & | *x*, |
|  |  | size\_t | *i1*, |
|  |  | size\_t | *i2*, |
|  |  | size\_t | *i3*, |
|  |  | const char \* | *error\_msg*, |
|  |  | size\_t | *idx* |
|  | ) |  |  |

inline

Return a reference to the value of the specified vector at the specified base-one indexes.

If an index is out of range, throw a `std::out_of_range` exception with the specified error message and index indicated.

Parameters
:   |  |  |
    | --- | --- |
    | x | Vector from which to get a value. |
    | i1 | First index plus 1. |
    | i2 | Second index plus 1. |
    | i3 | Third index plus 1. |
    | error\_msg | Error message if an index is out of range. |
    | idx | Nested index level to report in error message if the index is out of range. |

Returns
:   Value of vector at indexes.

Template Parameters
:   |  |  |
    | --- | --- |
    | T | type of value. |

Definition at line 79 of file get\_base1\_lhs.hpp.

template<typename T >

|  |  |  |  |
| --- | --- | --- | --- |
| T& stan::math::get\_base1\_lhs | ( | std::vector< std::vector< std::vector< std::vector< T > > > > & | *x*, |
|  |  | size\_t | *i1*, |
|  |  | size\_t | *i2*, |
|  |  | size\_t | *i3*, |
|  |  | size\_t | *i4*, |
|  |  | const char \* | *error\_msg*, |
|  |  | size\_t | *idx* |
|  | ) |  |  |

inline

Return a reference to the value of the specified vector at the specified base-one indexes.

If an index is out of range, throw a `std::out_of_range` exception with the specified error message and index indicated.

Parameters
:   |  |  |
    | --- | --- |
    | x | Vector from which to get a value. |
    | i1 | First index plus 1. |
    | i2 | Second index plus 1. |
    | i3 | Third index plus 1. |
    | i4 | Fourth index plus 1. |
    | error\_msg | Error message if an index is out of range. |
    | idx | Nested index level to report in error message if the index is out of range. |

Returns
:   Value of vector at indexes.

Template Parameters
:   |  |  |
    | --- | --- |
    | T | type of value. |

Definition at line 108 of file get\_base1\_lhs.hpp.

template<typename T >

|  |  |  |  |
| --- | --- | --- | --- |
| T& stan::math::get\_base1\_lhs | ( | std::vector< std::vector< std::vector< std::vector< std::vector< T > > > > > & | *x*, |
|  |  | size\_t | *i1*, |
|  |  | size\_t | *i2*, |
|  |  | size\_t | *i3*, |
|  |  | size\_t | *i4*, |
|  |  | size\_t | *i5*, |
|  |  | const char \* | *error\_msg*, |
|  |  | size\_t | *idx* |
|  | ) |  |  |

inline

Return a reference to the value of the specified vector at the specified base-one indexes.

If an index is out of range, throw a `std::out_of_range` exception with the specified error message and index indicated.

Parameters
:   |  |  |
    | --- | --- |
    | x | Vector from which to get a value. |
    | i1 | First index plus 1. |
    | i2 | Second index plus 1. |
    | i3 | Third index plus 1. |
    | i4 | Fourth index plus 1. |
    | i5 | Fifth index plus 1. |
    | error\_msg | Error message if an index is out of range. |
    | idx | Nested index level to report in error message if the index is out of range. |

Returns
:   Value of vector at indexes.

Template Parameters
:   |  |  |
    | --- | --- |
    | T | type of value. |

Definition at line 139 of file get\_base1\_lhs.hpp.

template<typename T >

|  |  |  |  |
| --- | --- | --- | --- |
| T& stan::math::get\_base1\_lhs | ( | std::vector< std::vector< std::vector< std::vector< std::vector< std::vector< T > > > > > > & | *x*, |
|  |  | size\_t | *i1*, |
|  |  | size\_t | *i2*, |
|  |  | size\_t | *i3*, |
|  |  | size\_t | *i4*, |
|  |  | size\_t | *i5*, |
|  |  | size\_t | *i6*, |
|  |  | const char \* | *error\_msg*, |
|  |  | size\_t | *idx* |
|  | ) |  |  |

inline

Return a reference to the value of the specified vector at the specified base-one indexes.

If an index is out of range, throw a `std::out_of_range` exception with the specified error message and index indicated.

Parameters
:   |  |  |
    | --- | --- |
    | x | Vector from which to get a value. |
    | i1 | First index plus 1. |
    | i2 | Second index plus 1. |
    | i3 | Third index plus 1. |
    | i4 | Fourth index plus 1. |
    | i5 | Fifth index plus 1. |
    | i6 | Sixth index plus 1. |
    | error\_msg | Error message if an index is out of range. |
    | idx | Nested index level to report in error message if the index is out of range. |

Returns
:   Value of vector at indexes.

Template Parameters
:   |  |  |
    | --- | --- |
    | T | type of value. |

Definition at line 172 of file get\_base1\_lhs.hpp.

template<typename T >

|  |  |  |  |
| --- | --- | --- | --- |
| T& stan::math::get\_base1\_lhs | ( | std::vector< std::vector< std::vector< std::vector< std::vector< std::vector< std::vector< T > > > > > > > & | *x*, |
|  |  | size\_t | *i1*, |
|  |  | size\_t | *i2*, |
|  |  | size\_t | *i3*, |
|  |  | size\_t | *i4*, |
|  |  | size\_t | *i5*, |
|  |  | size\_t | *i6*, |
|  |  | size\_t | *i7*, |
|  |  | const char \* | *error\_msg*, |
|  |  | size\_t | *idx* |
|  | ) |  |  |

inline

Return a reference to the value of the specified vector at the specified base-one indexes.

If an index is out of range, throw a `std::out_of_range` exception with the specified error message and index indicated.

Parameters
:   |  |  |
    | --- | --- |
    | x | Vector from which to get a value. |
    | i1 | First index plus 1. |
    | i2 | Second index plus 1. |
    | i3 | Third index plus 1. |
    | i4 | Fourth index plus 1. |
    | i5 | Fifth index plus 1. |
    | i6 | Sixth index plus 1. |
    | i7 | Seventh index plus 1. |
    | error\_msg | Error message if an index is out of range. |
    | idx | Nested index level to report in error message if the index is out of range. |

Returns
:   Value of vector at indexes.

Template Parameters
:   |  |  |
    | --- | --- |
    | T | type of value. |

Definition at line 208 of file get\_base1\_lhs.hpp.

template<typename T >

|  |  |  |  |
| --- | --- | --- | --- |
| T& stan::math::get\_base1\_lhs | ( | std::vector< std::vector< std::vector< std::vector< std::vector< std::vector< std::vector< std::vector< T > > > > > > > > & | *x*, |
|  |  | size\_t | *i1*, |
|  |  | size\_t | *i2*, |
|  |  | size\_t | *i3*, |
|  |  | size\_t | *i4*, |
|  |  | size\_t | *i5*, |
|  |  | size\_t | *i6*, |
|  |  | size\_t | *i7*, |
|  |  | size\_t | *i8*, |
|  |  | const char \* | *error\_msg*, |
|  |  | size\_t | *idx* |
|  | ) |  |  |

inline

Return a reference to the value of the specified vector at the specified base-one indexes.

If an index is out of range, throw a `std::out_of_range` exception with the specified error message and index indicated.

Parameters
:   |  |  |
    | --- | --- |
    | x | Vector from which to get a value. |
    | i1 | First index plus 1. |
    | i2 | Second index plus 1. |
    | i3 | Third index plus 1. |
    | i4 | Fourth index plus 1. |
    | i5 | Fifth index plus 1. |
    | i6 | Sixth index plus 1. |
    | i7 | Seventh index plus 1. |
    | i8 | Eigth index plus 1. |
    | error\_msg | Error message if an index is out of range. |
    | idx | Nested index level to report in error message if the index is out of range. |

Returns
:   Value of vector at indexes.

Template Parameters
:   |  |  |
    | --- | --- |
    | T | type of value. |

Definition at line 246 of file get\_base1\_lhs.hpp.

template<typename T >

|  |  |  |  |
| --- | --- | --- | --- |
| Eigen::Block<Eigen::Matrix<T,Eigen::Dynamic,Eigen::Dynamic> > stan::math::get\_base1\_lhs | ( | Eigen::Matrix< T, Eigen::Dynamic, Eigen::Dynamic > & | *x*, |
|  |  | size\_t | *m*, |
|  |  | const char \* | *error\_msg*, |
|  |  | size\_t | *idx* |
|  | ) |  |  |

inline

Return a copy of the row of the specified vector at the specified base-one row index.

If the index is out of range, throw a `std::out_of_range` exception with the specified error message and index indicated.

**Warning**: Because a copy is involved, it is inefficient to access element of matrices by first using this method to get a row then using a second call to get the value at a specified column.

Parameters
:   |  |  |
    | --- | --- |
    | x | Matrix from which to get a row |
    | m | Index into matrix plus 1. |
    | error\_msg | Error message if the index is out of range. |
    | idx | Nested index level to report in error message if the index is out of range. |

Returns
:   Row of matrix at `i - 1`.

Template Parameters
:   |  |  |
    | --- | --- |
    | T | type of value. |

Definition at line 285 of file get\_base1\_lhs.hpp.

template<typename T >

|  |  |  |  |
| --- | --- | --- | --- |
| T& stan::math::get\_base1\_lhs | ( | Eigen::Matrix< T, Eigen::Dynamic, Eigen::Dynamic > & | *x*, |
|  |  | size\_t | *m*, |
|  |  | size\_t | *n*, |
|  |  | const char \* | *error\_msg*, |
|  |  | size\_t | *idx* |
|  | ) |  |  |

inline

Return a reference to the value of the specified matrix at the specified base-one row and column indexes.

If either index is out of range, throw a `std::out_of_range` exception with the specified error message and index indicated.

Parameters
:   |  |  |
    | --- | --- |
    | x | Matrix from which to get a row |
    | m | Row index plus 1. |
    | n | Column index plus 1. |
    | error\_msg | Error message if either index is out of range. |
    | idx | Nested index level to report in error message if either index is out of range. |

Returns
:   Value of matrix at row `m - 1` and column `n - 1`.

Template Parameters
:   |  |  |
    | --- | --- |
    | T | type of value. |

Definition at line 311 of file get\_base1\_lhs.hpp.

template<typename T >

|  |  |  |  |
| --- | --- | --- | --- |
| T& stan::math::get\_base1\_lhs | ( | Eigen::Matrix< T, Eigen::Dynamic, 1 > & | *x*, |
|  |  | size\_t | *m*, |
|  |  | const char \* | *error\_msg*, |
|  |  | size\_t | *idx* |
|  | ) |  |  |

inline

Return a reference to the value of the specified column vector at the specified base-one index.

If the index is out of range, throw a `std::out_of_range` exception with the specified error message and index indicated.

Parameters
:   |  |  |
    | --- | --- |
    | x | Column vector from which to get a value. |
    | m | Row index plus 1. |
    | error\_msg | Error message if the index is out of range. |
    | idx | Nested index level to report in error message if the index is out of range. |

Returns
:   Value of column vector at row `m - 1`.

Template Parameters
:   |  |  |
    | --- | --- |
    | T | type of value. |

Definition at line 337 of file get\_base1\_lhs.hpp.

template<typename T >

|  |  |  |  |
| --- | --- | --- | --- |
| T& stan::math::get\_base1\_lhs | ( | Eigen::Matrix< T, 1, Eigen::Dynamic > & | *x*, |
|  |  | size\_t | *n*, |
|  |  | const char \* | *error\_msg*, |
|  |  | size\_t | *idx* |
|  | ) |  |  |

inline

Return a reference to the value of the specified row vector at the specified base-one index.

If the index is out of range, throw a `std::out_of_range` exception with the specified error message and index indicated.

Parameters
:   |  |  |
    | --- | --- |
    | x | Row vector from which to get a value. |
    | n | Column index plus 1. |
    | error\_msg | Error message if the index is out of range. |
    | idx | Nested index level to report in error message if the index is out of range. |

Returns
:   Value of row vector at column `n - 1`.

Template Parameters
:   |  |  |
    | --- | --- |
    | T | type of value. |

Definition at line 362 of file get\_base1\_lhs.hpp.

template<typename T\_lp , typename T\_lp\_accum >

|  |  |  |  |
| --- | --- | --- | --- |
| boost::math::tools::promote\_args<T\_lp, T\_lp\_accum>::type stan::math::get\_lp | ( | const T\_lp & | *lp*, |
|  |  | const stan::math::accumulator< T\_lp\_accum > & | *lp\_accum* |
|  | ) |  |  |

inline

Definition at line 11 of file get\_lp.hpp.

|  |  |  |  |
| --- | --- | --- | --- |
| void stan::math::grad2F1 | ( | double & | *gradA*, |
|  |  | double & | *gradC*, |
|  |  | double | *a*, |
|  |  | double | *b*, |
|  |  | double | *c*, |
|  |  | double | *z*, |
|  |  | double | *precision* = `1e-6` |
|  | ) |  |  |

Definition at line 102 of file internal\_math.hpp.

|  |  |  |  |
| --- | --- | --- | --- |
| void stan::math::gradF32 | ( | double \* | *g*, |
|  |  | double | *a*, |
|  |  | double | *b*, |
|  |  | double | *c*, |
|  |  | double | *d*, |
|  |  | double | *e*, |
|  |  | double | *z*, |
|  |  | double | *precision* = `1e-6` |
|  | ) |  |  |

Definition at line 50 of file internal\_math.hpp.

|  |  |  |  |
| --- | --- | --- | --- |
| void stan::math::gradIncBeta | ( | double & | *g1*, |
|  |  | double & | *g2*, |
|  |  | double | *a*, |
|  |  | double | *b*, |
|  |  | double | *z* |
|  | ) |  |  |

Definition at line 140 of file internal\_math.hpp.

|  |  |  |  |
| --- | --- | --- | --- |
| void stan::math::gradRegIncBeta | ( | double & | *g1*, |
|  |  | double & | *g2*, |
|  |  | double | *a*, |
|  |  | double | *b*, |
|  |  | double | *z*, |
|  |  | double | *digammaA*, |
|  |  | double | *digammaB*, |
|  |  | double | *digammaSum*, |
|  |  | double | *betaAB* |
|  | ) |  |  |

Definition at line 161 of file internal\_math.hpp.

|  |  |  |  |
| --- | --- | --- | --- |
| double stan::math::gradRegIncGamma | ( | double | *a*, |
|  |  | double | *z*, |
|  |  | double | *g*, |
|  |  | double | *dig*, |
|  |  | double | *precision* = `1e-6` |
|  | ) |  |  |

Definition at line 181 of file internal\_math.hpp.

template<typename T >

|  |  |  |  |
| --- | --- | --- | --- |
| Eigen::Matrix<T,Eigen::Dynamic,1> stan::math::head | ( | const Eigen::Matrix< T, Eigen::Dynamic, 1 > & | *v*, |
|  |  | size\_t | *n* |
|  | ) |  |  |

inline

Return the specified number of elements as a vector from the front of the specified vector.

Template Parameters
:   |  |  |
    | --- | --- |
    | T | Type of value in vector |

Parameters
:   |  |  |
    | --- | --- |
    | v | Vector input |
    | n | Size of return |

Returns
:   The first n elements of v

Definition at line 24 of file head.hpp.

template<typename T >

|  |  |  |  |
| --- | --- | --- | --- |
| Eigen::Matrix<T,1,Eigen::Dynamic> stan::math::head | ( | const Eigen::Matrix< T, 1, Eigen::Dynamic > & | *rv*, |
|  |  | size\_t | *n* |
|  | ) |  |  |

inline

Return the specified number of elements as a row vector from the front of the specified row vector.

Template Parameters
:   |  |  |
    | --- | --- |
    | T | Type of value in vector |

Parameters
:   |  |  |
    | --- | --- |
    | rv | Row vector |
    | n | Size of return row vector |

Returns
:   The first n elements of rv

Definition at line 42 of file head.hpp.

template<typename T >

|  |  |  |  |
| --- | --- | --- | --- |
| std::vector<T> stan::math::head | ( | const std::vector< T > & | *sv*, |
|  |  | size\_t | *n* |
|  | ) |  |  |

Return the specified number of elements as a standard vector from the front of the specified standard vector.

Template Parameters
:   |  |  |
    | --- | --- |
    | T | Type of value in vector |

Parameters
:   |  |  |
    | --- | --- |
    | sv | Standard vector |
    | n | Size of return |

Returns
:   The first n elements of sv

Definition at line 58 of file head.hpp.

|  |  |  |  |
| --- | --- | --- | --- |
| double stan::math::ibeta | ( | const double | *a*, |
|  |  | const double | *b*, |
|  |  | const double | *x* |
|  | ) |  |  |

inline

The normalized incomplete beta function of a, b, and x.

Used to compute the cumulative density function for the beta distribution.

Parameters
:   |  |  |
    | --- | --- |
    | a | Shape parameter a <= 0; a and b can't both be 0 |
    | b | Shape parameter b <= 0 |
    | x | Random variate. 0 <= x <= 1 |

Exceptions
:   |  |  |
    | --- | --- |
    | if | constraints are violated or if any argument is NaN |

Returns
:   The normalized incomplete beta function.

Definition at line 23 of file ibeta.hpp.

template<typename T\_true , typename T\_false >

|  |  |  |  |
| --- | --- | --- | --- |
| boost::math::tools::promote\_args<T\_true,T\_false>::type stan::math::if\_else | ( | const bool | *c*, |
|  |  | const T\_true | *y\_true*, |
|  |  | const T\_false | *y\_false* |
|  | ) |  |  |

inline

Return the second argument if the first argument is true and otherwise return the second argument.

This is just a convenience method to provide a function with the same behavior as the built-in ternary operator. In general, this function behaves as if defined by

`if_else(c,y1,y0) = c ? y1 : y0`.

Parameters
:   |  |  |
    | --- | --- |
    | c | Boolean condition value. |
    | y\_true | Value to return if condition is true. |
    | y\_false | Value to return if condition is false. |

Definition at line 25 of file if\_else.hpp.

template<typename T >

|  |  |  |  |
| --- | --- | --- | --- |
| void stan::math::initialize | ( | T & | *x*, |
|  |  | const T & | *v* |
|  | ) |  |  |

inline

Definition at line 17 of file initialize.hpp.

template<typename T , typename V >

|  |  |  |  |
| --- | --- | --- | --- |
| boost::enable\_if\_c<boost::is\_arithmetic<V>::value, void>::type stan::math::initialize | ( | T & | *x*, |
|  |  | V | *v* |
|  | ) |  |  |

inline

Definition at line 23 of file initialize.hpp.

template<typename T , int R, int C, typename V >

|  |  |  |  |
| --- | --- | --- | --- |
| void stan::math::initialize | ( | Eigen::Matrix< T, R, C > & | *x*, |
|  |  | const V & | *v* |
|  | ) |  |  |

inline

Definition at line 27 of file initialize.hpp.

template<typename T , typename V >

|  |  |  |  |
| --- | --- | --- | --- |
| void stan::math::initialize | ( | std::vector< T > & | *x*, |
|  |  | const V & | *v* |
|  | ) |  |  |

inline

Definition at line 32 of file initialize.hpp.

template<typename T >

|  |  |  |  |  |  |
| --- | --- | --- | --- | --- | --- |
| unsigned int stan::math::int\_step | ( | const T | *y* | ) |  |

The integer step, or Heaviside, function.

For double NaN input, int\_step(NaN) returns 0.

Parameters
:   |  |  |
    | --- | --- |
    | y | Value to test. |

Returns
:   1 if value is greater than 0 and 0 otherwise

Template Parameters
:   |  |  |
    | --- | --- |
    | T | Scalar argument type. |

Definition at line 25 of file int\_step.hpp.

template<typename F , typename T1 , typename T2 >

|  |  |  |  |
| --- | --- | --- | --- |
| std::vector<std::vector<typename stan::return\_type<T1,T2>::type> > stan::math::integrate\_ode | ( | const F & | *f*, |
|  |  | const std::vector< T1 > | *y0*, |
|  |  | const double | *t0*, |
|  |  | const std::vector< double > & | *ts*, |
|  |  | const std::vector< T2 > & | *theta*, |
|  |  | const std::vector< double > & | *x*, |
|  |  | const std::vector< int > & | *x\_int*, |
|  |  | std::ostream \* | *msgs* |
|  | ) |  |  |

Return the solutions for the specified system of ordinary differential equations given the specified initial state, initial times, times of desired solution, and parameters and data, writing error and warning messages to the specified stream.

**Warning:** If the system of equations is stiff, roughly defined by having varying time scales across dimensions, then this solver is likely to be slow.

This function is templated to allow the initial times to be either data or autodiff variables and the parameters to be data or autodiff variables. The autodiff-based implementation for reverse-mode are defined in namespace `stan::agrad` and may be invoked via argument-dependent lookup by including their headers.

This function uses the Dormand-Prince method as implemented in Boost's  `boost::numeric::odeint::runge_kutta_dopri5` integrator.

Template Parameters
:   |  |  |
    | --- | --- |
    | F | type of ODE system function. |
    | T1 | type of scalars for initial values. |
    | T2 | type of scalars for parameters. |

Parameters
:   |  |  |  |
    | --- | --- | --- |
    | [in] | f | functor for the base ordinary differential equation. |
    | [in] | y0 | initial state. |
    | [in] | t0 | initial time. |
    | [in] | ts | times of the desired solutions, in strictly increasing order, all greater than the initial time. |
    | [in] | theta | parameter vector for the ODE. |
    | [in] | x | continuous data vector for the ODE. |
    | [in] | x\_int | integer data vector for the ODE. |
    | [in,out] | msgs | the print stream for warning messages. |

Returns
:   a vector of states, each state being a vector of the same size as the state variable, corresponding to a time in ts.

Definition at line 61 of file integrate\_ode.hpp.

template<typename T >

|  |  |  |  |  |  |
| --- | --- | --- | --- | --- | --- |
| boost::math::tools::promote\_args<T>::type stan::math::inv | ( | const T | *x* | ) |  |

inline

Definition at line 12 of file inv.hpp.

template<typename T >

|  |  |  |  |  |  |
| --- | --- | --- | --- | --- | --- |
| boost::math::tools::promote\_args<T>::type stan::math::inv\_cloglog | ( | T | *x* | ) |  |

inline

The inverse complementary log-log function.

The function is defined by

`inv_cloglog(x) = 1 - exp(-exp(x))`.

This function can be used to implement the inverse link function for complementary-log-log regression.

Parameters
:   |  |  |
    | --- | --- |
    | x | Argument. |

Returns
:   Inverse complementary log-log of the argument.

Definition at line 49 of file inv\_cloglog.hpp.

template<typename T >

|  |  |  |  |  |  |
| --- | --- | --- | --- | --- | --- |
| boost::math::tools::promote\_args<T>::type stan::math::inv\_logit | ( | const T | *a* | ) |  |

inline

Returns the inverse logit function applied to the argument.

The inverse logit function is defined by

.

This function can be used to implement the inverse link function for logistic regression.

The inverse to this function is `stan::math::logit`.

Parameters
:   |  |  |
    | --- | --- |
    | a | Argument. |

Returns
:   Inverse logit of argument.

Definition at line 52 of file inv\_logit.hpp.

template<typename T >

|  |  |  |  |  |  |
| --- | --- | --- | --- | --- | --- |
| boost::math::tools::promote\_args<T>::type stan::math::inv\_sqrt | ( | const T | *x* | ) |  |

inline

Definition at line 12 of file inv\_sqrt.hpp.

template<typename T >

|  |  |  |  |  |  |
| --- | --- | --- | --- | --- | --- |
| boost::math::tools::promote\_args<T>::type stan::math::inv\_square | ( | const T | *x* | ) |  |

inline

Definition at line 12 of file inv\_square.hpp.

template<typename T >

|  |  |  |  |  |  |
| --- | --- | --- | --- | --- | --- |
| Eigen::Matrix<T,Eigen::Dynamic,Eigen::Dynamic> stan::math::inverse | ( | const Eigen::Matrix< T, Eigen::Dynamic, Eigen::Dynamic > & | *m* | ) |  |

inline

Returns the inverse of the specified matrix.

Parameters
:   |  |  |
    | --- | --- |
    | m | Specified matrix. |

Returns
:   Inverse of the matrix.

Definition at line 18 of file inverse.hpp.

template<typename Vector >

|  |  |  |  |
| --- | --- | --- | --- |
| void stan::math::inverse\_softmax | ( | const Vector & | *simplex*, |
|  |  | Vector & | *y* |
|  | ) |  |  |

Writes the inverse softmax of the simplex argument into the second argument.

See `stan::math::softmax` for the inverse function and a definition of the relation.

The inverse softmax function is defined by

.

This function defines the inverse of `stan::math::softmax` up to a scaling factor.

Because of the definition, values of 0.0 in the simplex are converted to negative infinity, and values of 1.0 are converted to 0.0.

There is no check that the input vector is a valid simplex vector.

Parameters
:   |  |  |
    | --- | --- |
    | simplex | Simplex vector input. |
    | y | Vector into which the inverse softmax is written. |

Exceptions
:   |  |  |
    | --- | --- |
    | std::invalid\_argument | if size of the input and output vectors differ. |

Definition at line 34 of file inverse\_softmax.hpp.

template<typename T >

|  |  |  |  |  |  |
| --- | --- | --- | --- | --- | --- |
| Eigen::Matrix<T,Eigen::Dynamic,Eigen::Dynamic> stan::math::inverse\_spd | ( | const Eigen::Matrix< T, Eigen::Dynamic, Eigen::Dynamic > & | *m* | ) |  |

inline

Returns the inverse of the specified symmetric, pos/neg-definite matrix.

Parameters
:   |  |  |
    | --- | --- |
    | m | Specified matrix. |

Returns
:   Inverse of the matrix.

Definition at line 19 of file inverse\_spd.hpp.

|  |  |  |  |  |  |
| --- | --- | --- | --- | --- | --- |
| int stan::math::is\_inf | ( | const double | *x* | ) |  |

inline

Returns 1 if the input is infinite and 0 otherwise.

Delegates to `boost::math::isinf`.

Parameters
:   |  |  |
    | --- | --- |
    | x | Value to test. |

Returns
:   `1` if the value is infinite.

Definition at line 19 of file is\_inf.hpp.

|  |  |  |  |  |  |
| --- | --- | --- | --- | --- | --- |
| int stan::math::is\_nan | ( | const double | *x* | ) |  |

inline

Returns 1 if the input is NaN and 0 otherwise.

Delegates to `boost::math::isnan`.

Parameters
:   |  |  |
    | --- | --- |
    | x | Value to test. |

Returns
:   `1` if the value is NaN.

Definition at line 19 of file is\_nan.hpp.

template<typename T >

|  |  |  |  |  |  |
| --- | --- | --- | --- | --- | --- |
| bool stan::math::is\_uninitialized | ( | T | *x* | ) |  |

inline

Returns `true` if the specified variable is uninitialized.

Arithmetic types are always initialized by definition (the value is not specified).

Template Parameters
:   |  |  |
    | --- | --- |
    | T | Type of object to test. |

Parameters
:   |  |  |
    | --- | --- |
    | x | Object to test. |

Returns
:   `true` if the specified object is uninitialized. false if input is NaN.

Definition at line 19 of file is\_uninitialized.hpp.

template<typename T1 , typename T2 >

|  |  |  |  |
| --- | --- | --- | --- |
| boost::math::tools::promote\_args<T1,T2>::type stan::math::lbeta | ( | const T1 | *a*, |
|  |  | const T2 | *b* |
|  | ) |  |  |

inline

Return the log of the beta function applied to the specified arguments.

The beta function is defined for  and  by

.

This function returns its log,

.

See boost::math::lgamma() for the double-based and stan::agrad for the variable-based log Gamma function.

Parameters
:   |  |  |
    | --- | --- |
    | a | First value |
    | b | Second value |

Returns
:   Log of the beta function applied to the two values.

Template Parameters
:   |  |  |
    | --- | --- |
    | T1 | Type of first value. |
    | T2 | Type of second value. |

Definition at line 59 of file lbeta.hpp.

|  |  |  |  |  |  |
| --- | --- | --- | --- | --- | --- |
| double stan::math::lgamma | ( | double | *x* | ) |  |

Definition at line 31 of file lgamma.hpp.

template<typename T >

|  |  |  |  |
| --- | --- | --- | --- |
| boost::math::tools::promote\_args<T>::type stan::math::lmgamma | ( | const int | *k*, |
|  |  | T | *x* |
|  | ) |  |  |

inline

Return the natural logarithm of the multivariate gamma function with the speciifed dimensions and argument.

The multivariate gamma function  for dimensionality  and argument  is defined by

,

where  is the gamma function.

Parameters
:   |  |  |
    | --- | --- |
    | k | Number of dimensions. |
    | x | Function argument. |

Returns
:   Natural log of the multivariate gamma function.

Template Parameters
:   |  |  |
    | --- | --- |
    | T | Type of scalar. |

Definition at line 57 of file lmgamma.hpp.

template<typename T , int Rows, int Cols>

|  |  |  |  |  |  |
| --- | --- | --- | --- | --- | --- |
| Eigen::Matrix<T,Rows,Cols> stan::math::log | ( | const Eigen::Matrix< T, Rows, Cols > & | *m* | ) |  |

inline

Return the element-wise logarithm of the matrix or vector.

Parameters
:   |  |  |
    | --- | --- |
    | m | The matrix or vector. |

Returns
:   ret(i,j) = log(m(i,j))

Definition at line 16 of file log.hpp.

|  |  |  |  |  |
| --- | --- | --- | --- | --- |
| double stan::math::log10 | ( |  | ) |  |

inline

Return natural logarithm of ten.

Returns
:   Natural logarithm of ten.

Definition at line 105 of file constants.hpp.

template<typename T >

|  |  |  |  |  |  |
| --- | --- | --- | --- | --- | --- |
| boost::math::tools::promote\_args<T>::type stan::math::log1m | ( | T | *x* | ) |  |

inline

Return the natural logarithm of one minus the specified value.

The main use of this function is to cut down on intermediate values during algorithmic differentiation.

Parameters
:   |  |  |
    | --- | --- |
    | x | Specified value. |

Returns
:   Natural log of one minus `x`.

Definition at line 40 of file log1m.hpp.

template<typename T >

|  |  |  |  |  |  |
| --- | --- | --- | --- | --- | --- |
| boost::math::tools::promote\_args<T>::type stan::math::log1m\_exp | ( | const T | *a* | ) |  |

inline

Calculates the log of 1 minus the exponential of the specified value without overflow log1m\_exp(x) = log(1-exp(x)).

This function is only defined for x<0

Definition at line 40 of file log1m\_exp.hpp.

template<typename T >

|  |  |  |  |  |  |
| --- | --- | --- | --- | --- | --- |
| boost::math::tools::promote\_args<T>::type stan::math::log1m\_inv\_logit | ( | const T | *u* | ) |  |

inline

Returns the natural logarithm of 1 minus the inverse logit of the specified argument.

Template Parameters
:   |  |  |
    | --- | --- |
    | T | Scalar type |

Parameters
:   |  |  |
    | --- | --- |
    | u | Input. |

Returns
:   log of 1 minus the inverse logit of the input.

Definition at line 36 of file log1m\_inv\_logit.hpp.

template<typename T >

|  |  |  |  |  |  |
| --- | --- | --- | --- | --- | --- |
| boost::math::tools::promote\_args<T>::type stan::math::log1p | ( | const T | *x* | ) |  |

inline

Return the natural logarithm of one plus the specified value.

The main use of this function is to cut down on intermediate values during algorithmic differentiation.

Parameters
:   |  |  |
    | --- | --- |
    | x | Specified value. |

Returns
:   Natural log of one plus `x`.

Definition at line 39 of file log1p.hpp.

template<typename T >

|  |  |  |  |  |  |
| --- | --- | --- | --- | --- | --- |
| boost::math::tools::promote\_args<T>::type stan::math::log1p\_exp | ( | const T | *a* | ) |  |

inline

Calculates the log of 1 plus the exponential of the specified value without overflow.

This function is related to other special functions by:

`log1p_exp(x)`

`= log1p(exp(a))`

`= log(1 + exp(x))`

`= log_sum_exp(0,x)`.

Definition at line 44 of file log1p\_exp.hpp.

template<typename T >

|  |  |  |  |  |  |
| --- | --- | --- | --- | --- | --- |
| boost::math::tools::promote\_args<T>::type stan::math::log2 | ( | const T | *a* | ) |  |

inline

Returns the base 2 logarithm of the argument (C99).

The function is defined by:

`log2(a) = log(a) / std::log(2.0)`.

Template Parameters
:   |  |  |
    | --- | --- |
    | T | type of scalar |

Parameters
:   |  |  |
    | --- | --- |
    | a | Value. |

Returns
:   Base 2 logarithm of the value.

Definition at line 25 of file log2.hpp.

|  |  |  |  |  |
| --- | --- | --- | --- | --- |
| double stan::math::log2 | ( |  | ) |  |

inline

Return natural logarithm of two.

Returns
:   Natural logarithm of two.

Definition at line 35 of file log2.hpp.

template<typename T , int R, int C>

|  |  |  |  |  |  |
| --- | --- | --- | --- | --- | --- |
| T stan::math::log\_determinant | ( | const Eigen::Matrix< T, R, C > & | *m* | ) |  |

inline

Returns the log absolute determinant of the specified square matrix.

Parameters
:   |  |  |
    | --- | --- |
    | m | Specified matrix. |

Returns
:   log absolute determinant of the matrix.

Exceptions
:   |  |  |
    | --- | --- |
    | std::domain\_error | if matrix is not square. |

Definition at line 18 of file log\_determinant.hpp.

template<int R, int C, typename T >

|  |  |  |  |  |  |
| --- | --- | --- | --- | --- | --- |
| T stan::math::log\_determinant\_ldlt | ( | stan::math::LDLT\_factor< T, R, C > & | *A* | ) |  |

inline

Definition at line 12 of file log\_determinant\_ldlt.hpp.

template<typename T , int R, int C>

|  |  |  |  |  |  |
| --- | --- | --- | --- | --- | --- |
| T stan::math::log\_determinant\_spd | ( | const Eigen::Matrix< T, R, C > & | *m* | ) |  |

inline

Returns the log absolute determinant of the specified square matrix.

Parameters
:   |  |  |
    | --- | --- |
    | m | Specified matrix. |

Returns
:   log absolute determinant of the matrix.

Exceptions
:   |  |  |
    | --- | --- |
    | std::domain\_error | if matrix is not square. |

Definition at line 20 of file log\_determinant\_spd.hpp.

template<typename T1 , typename T2 >

|  |  |  |  |
| --- | --- | --- | --- |
| boost::math::tools::promote\_args<T1,T2>::type stan::math::log\_diff\_exp | ( | const T1 | *x*, |
|  |  | const T2 | *y* |
|  | ) |  |  |

inline

The natural logarithm of the difference of the natural exponentiation of x1 and the natural exponentiation of x2.

This function is only defined for x<0

Definition at line 49 of file log\_diff\_exp.hpp.

template<typename T1 , typename T2 >

|  |  |  |  |
| --- | --- | --- | --- |
| boost::math::tools::promote\_args<T1,T2>::type stan::math::log\_falling\_factorial | ( | const T1 | *x*, |
|  |  | const T2 | *n* |
|  | ) |  |  |

inline

Definition at line 41 of file log\_falling\_factorial.hpp.

template<typename T >

|  |  |  |  |  |  |
| --- | --- | --- | --- | --- | --- |
| boost::math::tools::promote\_args<T>::type stan::math::log\_inv\_logit | ( | const T & | *u* | ) |  |

inline

Returns the natural logarithm of the inverse logit of the specified argument.

Template Parameters
:   |  |  |
    | --- | --- |
    | T | Scalar type |

Parameters
:   |  |  |
    | --- | --- |
    | u | Input. |

Returns
:   log of the inverse logit of the input.

Definition at line 36 of file log\_inv\_logit.hpp.

template<typename T1 , typename T2 >

|  |  |  |  |
| --- | --- | --- | --- |
| boost::math::tools::promote\_args<T1,T2>::type stan::math::log\_rising\_factorial | ( | const T1 | *x*, |
|  |  | const T2 | *n* |
|  | ) |  |  |

inline

Definition at line 41 of file log\_rising\_factorial.hpp.

template<typename T >

|  |  |  |  |  |  |
| --- | --- | --- | --- | --- | --- |
| Eigen::Matrix<T,Eigen::Dynamic,1> stan::math::log\_softmax | ( | const Eigen::Matrix< T, Eigen::Dynamic, 1 > & | *v* | ) |  |

inline

Return the natural logarithm of the softmax of the specified vector.

For the log softmax function, the entries in the Jacobian are

Template Parameters
:   |  |  |
    | --- | --- |
    | T | Scalar type of values in vector. |

Parameters
:   |  |  |  |
    | --- | --- | --- |
    | [in] | v | Vector to transform. |

Returns
:   Unit simplex result of the softmax transform of the vector.

Definition at line 44 of file log\_softmax.hpp.

template<int R, int C>

|  |  |  |  |  |  |
| --- | --- | --- | --- | --- | --- |
| double stan::math::log\_sum\_exp | ( | const Eigen::Matrix< double, R, C > & | *x* | ) |  |

Return the log of the sum of the exponentiated values of the specified matrix of values.

The matrix may be a full matrix, a vector, or a row vector.

The function is defined as follows to prevent overflow in exponential calculations.

.

Parameters
:   |  |  |  |
    | --- | --- | --- |
    | [in] | x | Matrix of specified values |

Returns
:   The log of the sum of the exponentiated vector values.

Definition at line 29 of file log\_sum\_exp.hpp.

template<typename T1 , typename T2 >

|  |  |  |  |
| --- | --- | --- | --- |
| boost::math::tools::promote\_args<T1,T2>::type stan::math::log\_sum\_exp | ( | const T2 & | *a*, |
|  |  | const T1 & | *b* |
|  | ) |  |  |

inline

Calculates the log sum of exponetials without overflow.

,

where .

Parameters
:   |  |  |
    | --- | --- |
    | a | the first variable |
    | b | the second variable |

Definition at line 49 of file log\_sum\_exp.hpp.

|  |  |  |  |  |  |
| --- | --- | --- | --- | --- | --- |
| double stan::math::log\_sum\_exp | ( | const std::vector< double > & | *x* | ) |  |

Return the log of the sum of the exponentiated values of the specified sequence of values.

The function is defined as follows to prevent overflow in exponential calculations.

.

Parameters
:   |  |  |  |
    | --- | --- | --- |
    | [in] | x | array of specified values |

Returns
:   The log of the sum of the exponentiated vector values.

Definition at line 68 of file log\_sum\_exp.hpp.

template<typename T1 , typename T2 >

|  |  |  |  |
| --- | --- | --- | --- |
| int stan::math::logical\_and | ( | const T1 | *x1*, |
|  |  | const T2 | *x2* |
|  | ) |  |  |

inline

The logical and function which returns 1 if both arguments are unequal to zero and 0 otherwise.

Equivalent to `x1 != 0 && x2 != 0`.

Template Parameters
:   |  |  |
    | --- | --- |
    | T1 | Type of first argument. |
    | T2 | Type of second argument. |

Parameters
:   |  |  |
    | --- | --- |
    | x1 | First argument |
    | x2 | Second argument |

Returns
:   `true` if both x1 and x2 are not equal to 0.

Definition at line 30 of file logical\_and.hpp.

template<typename T1 , typename T2 >

|  |  |  |  |
| --- | --- | --- | --- |
| int stan::math::logical\_eq | ( | const T1 | *x1*, |
|  |  | const T2 | *x2* |
|  | ) |  |  |

inline

Return 1 if the first argument is equal to the second.

Equivalent to `x1 == x2`.

Template Parameters
:   |  |  |
    | --- | --- |
    | T1 | Type of first argument. |
    | T2 | Type of second argument. |

Parameters
:   |  |  |
    | --- | --- |
    | x1 | First argument |
    | x2 | Second argument |

Returns
:   `true` iff `x1 == x2`

Definition at line 19 of file logical\_eq.hpp.

template<typename T1 , typename T2 >

|  |  |  |  |
| --- | --- | --- | --- |
| int stan::math::logical\_gt | ( | const T1 | *x1*, |
|  |  | const T2 | *x2* |
|  | ) |  |  |

inline

Return 1 if the first argument is strictly greater than the second.

Equivalent to `x1 < x2`.

Template Parameters
:   |  |  |
    | --- | --- |
    | T1 | Type of first argument. |
    | T2 | Type of second argument. |

Parameters
:   |  |  |
    | --- | --- |
    | x1 | First argument |
    | x2 | Second argument |

Returns
:   `true` iff `x1 > x2`

Definition at line 19 of file logical\_gt.hpp.

template<typename T1 , typename T2 >

|  |  |  |  |
| --- | --- | --- | --- |
| int stan::math::logical\_gte | ( | const T1 | *x1*, |
|  |  | const T2 | *x2* |
|  | ) |  |  |

inline

Return 1 if the first argument is greater than or equal to the second.

Equivalent to `x1 >= x2`.

Template Parameters
:   |  |  |
    | --- | --- |
    | T1 | Type of first argument. |
    | T2 | Type of second argument. |

Parameters
:   |  |  |
    | --- | --- |
    | x1 | First argument |
    | x2 | Second argument |

Returns
:   `true` iff `x1 >= x2`

Definition at line 19 of file logical\_gte.hpp.

template<typename T1 , typename T2 >

|  |  |  |  |
| --- | --- | --- | --- |
| int stan::math::logical\_lt | ( | T1 | *x1*, |
|  |  | T2 | *x2* |
|  | ) |  |  |

inline

Return 1 if the first argument is strictly less than the second.

Equivalent to `x1 < x2`.

Template Parameters
:   |  |  |
    | --- | --- |
    | T1 | Type of first argument. |
    | T2 | Type of second argument. |

Parameters
:   |  |  |
    | --- | --- |
    | x1 | First argument |
    | x2 | Second argument |

Returns
:   `true` iff `x1 < x2`

Definition at line 20 of file logical\_lt.hpp.

template<typename T1 , typename T2 >

|  |  |  |  |
| --- | --- | --- | --- |
| int stan::math::logical\_lte | ( | const T1 | *x1*, |
|  |  | const T2 | *x2* |
|  | ) |  |  |

inline

Return 1 if the first argument is less than or equal to the second.

Equivalent to `x1 <= x2`.

Template Parameters
:   |  |  |
    | --- | --- |
    | T1 | Type of first argument. |
    | T2 | Type of second argument. |

Parameters
:   |  |  |
    | --- | --- |
    | x1 | First argument |
    | x2 | Second argument |

Returns
:   `true` iff `x1 <= x2`

Definition at line 19 of file logical\_lte.hpp.

template<typename T >

|  |  |  |  |  |  |
| --- | --- | --- | --- | --- | --- |
| int stan::math::logical\_negation | ( | const T | *x* | ) |  |

inline

The logical negation function which returns 1 if the input is equal to zero and 0 otherwise.

Template Parameters
:   |  |  |
    | --- | --- |
    | T | Type to compare to zero. |

Parameters
:   |  |  |
    | --- | --- |
    | x | Value to compare to zero. |

Returns
:   1 if input is equal to zero.

Definition at line 17 of file logical\_negation.hpp.

template<typename T1 , typename T2 >

|  |  |  |  |
| --- | --- | --- | --- |
| int stan::math::logical\_neq | ( | const T1 | *x1*, |
|  |  | const T2 | *x2* |
|  | ) |  |  |

inline

Return 1 if the first argument is unequal to the second.

Equivalent to `x1 != x2`.

Template Parameters
:   |  |  |
    | --- | --- |
    | T1 | Type of first argument. |
    | T2 | Type of second argument. |

Parameters
:   |  |  |
    | --- | --- |
    | x1 | First argument |
    | x2 | Second argument |

Returns
:   `true` iff `x1 != x2`

Definition at line 19 of file logical\_neq.hpp.

template<typename T1 , typename T2 >

|  |  |  |  |
| --- | --- | --- | --- |
| int stan::math::logical\_or | ( | T1 | *x1*, |
|  |  | T2 | *x2* |
|  | ) |  |  |

inline

The logical or function which returns 1 if either argument is unequal to zero and 0 otherwise.

Equivalent to `x1 != 0 || x2 != 0`.

Template Parameters
:   |  |  |
    | --- | --- |
    | T1 | Type of first argument. |
    | T2 | Type of second argument. |

Parameters
:   |  |  |
    | --- | --- |
    | x1 | First argument |
    | x2 | Second argument |

Returns
:   `true` if either x1 or x2 is not equal to 0.

Definition at line 29 of file logical\_or.hpp.

template<typename T >

|  |  |  |  |  |  |
| --- | --- | --- | --- | --- | --- |
| boost::math::tools::promote\_args<T>::type stan::math::logit | ( | const T | *a* | ) |  |

inline

Returns the logit function applied to the argument.

The logit function is defined as for  by returning the log odds of  treated as a probability,

.

The inverse to this function is `stan::math::inv_logit`.

Parameters
:   |  |  |
    | --- | --- |
    | a | Argument. |

Returns
:   Logit of the argument.

Definition at line 44 of file logit.hpp.

|  |  |  |  |  |
| --- | --- | --- | --- | --- |
| double stan::math::machine\_precision | ( |  | ) |  |

inline

Returns the difference between 1.0 and the next value representable.

Returns
:   Minimum positive number.

Definition at line 142 of file constants.hpp.

|  |  |  |  |
| --- | --- | --- | --- |
| double stan::math::max | ( | const double | *a*, |
|  |  | const double | *b* |
|  | ) |  |  |

inline

Definition at line 7 of file max.hpp.

|  |  |  |  |  |  |
| --- | --- | --- | --- | --- | --- |
| int stan::math::max | ( | const std::vector< int > & | *x* | ) |  |

inline

Returns the maximum coefficient in the specified column vector.

Parameters
:   |  |  |
    | --- | --- |
    | x | Specified vector. |

Returns
:   Maximum coefficient value in the vector.

Template Parameters
:   |  |  |
    | --- | --- |
    | Type | of values being compared and returned |

Exceptions
:   |  |  |
    | --- | --- |
    | std::domain\_error | If the size of the vector is zero. |

Definition at line 19 of file max.hpp.

template<typename T >

|  |  |  |  |  |  |
| --- | --- | --- | --- | --- | --- |
| T stan::math::max | ( | const std::vector< T > & | *x* | ) |  |

inline

Returns the maximum coefficient in the specified column vector.

Parameters
:   |  |  |
    | --- | --- |
    | x | Specified vector. |

Returns
:   Maximum coefficient value in the vector.

Template Parameters
:   |  |  |
    | --- | --- |
    | T | Type of values being compared and returned |

Definition at line 37 of file max.hpp.

template<typename T , int R, int C>

|  |  |  |  |  |  |
| --- | --- | --- | --- | --- | --- |
| T stan::math::max | ( | const Eigen::Matrix< T, R, C > & | *m* | ) |  |

inline

Returns the maximum coefficient in the specified vector, row vector, or matrix.

Parameters
:   |  |  |
    | --- | --- |
    | m | Specified vector, row vector, or matrix. |

Returns
:   Maximum coefficient value in the vector.

Definition at line 54 of file max.hpp.

template<typename T1 , typename T2 , int R1, int C1, int R2, int C2>

|  |  |  |  |
| --- | --- | --- | --- |
| Eigen::Matrix<typename boost::math::tools::promote\_args<T1,T2>::type,R1,C2> stan::math::mdivide\_left | ( | const Eigen::Matrix< T1, R1, C1 > & | *A*, |
|  |  | const Eigen::Matrix< T2, R2, C2 > & | *b* |
|  | ) |  |  |

inline

Returns the solution of the system Ax=b.

Parameters
:   |  |  |
    | --- | --- |
    | A | Matrix. |
    | b | Right hand side matrix or vector. |

Returns
:   x = A^-1 b, solution of the linear system.

Exceptions
:   |  |  |
    | --- | --- |
    | std::domain\_error | if A is not square or the rows of b don't match the size of A. |

Definition at line 24 of file mdivide\_left.hpp.

template<int R1, int C1, int R2, int C2, typename T1 , typename T2 >

|  |  |  |  |
| --- | --- | --- | --- |
| Eigen::Matrix<typename boost::math::tools::promote\_args<T1,T2>::type,R1,C2> stan::math::mdivide\_left\_ldlt | ( | const stan::math::LDLT\_factor< T1, R1, C1 > & | *A*, |
|  |  | const Eigen::Matrix< T2, R2, C2 > & | *b* |
|  | ) |  |  |

inline

Returns the solution of the system Ax=b given an LDLT\_factor of A.

Parameters
:   |  |  |
    | --- | --- |
    | A | LDLT\_factor |
    | b | Right hand side matrix or vector. |

Returns
:   x = b A^-1, solution of the linear system.

Exceptions
:   |  |  |
    | --- | --- |
    | std::domain\_error | if rows of b don't match the size of A. |

Definition at line 24 of file mdivide\_left\_ldlt.hpp.

template<typename T1 , typename T2 , int R1, int C1, int R2, int C2>

|  |  |  |  |
| --- | --- | --- | --- |
| Eigen::Matrix<typename boost::math::tools::promote\_args<T1,T2>::type,R1,C2> stan::math::mdivide\_left\_spd | ( | const Eigen::Matrix< T1, R1, C1 > & | *A*, |
|  |  | const Eigen::Matrix< T2, R2, C2 > & | *b* |
|  | ) |  |  |

inline

Returns the solution of the system Ax=b where A is symmetric positive definite.

Parameters
:   |  |  |
    | --- | --- |
    | A | Matrix. |
    | b | Right hand side matrix or vector. |

Returns
:   x = A^-1 b, solution of the linear system.

Exceptions
:   |  |  |
    | --- | --- |
    | std::domain\_error | if A is not square or the rows of b don't match the size of A. |

Definition at line 27 of file mdivide\_left\_spd.hpp.

template<int TriView, typename T1 , typename T2 , int R1, int C1, int R2, int C2>

|  |  |  |  |
| --- | --- | --- | --- |
| Eigen::Matrix<typename boost::math::tools::promote\_args<T1,T2>::type, R1,C2> stan::math::mdivide\_left\_tri | ( | const Eigen::Matrix< T1, R1, C1 > & | *A*, |
|  |  | const Eigen::Matrix< T2, R2, C2 > & | *b* |
|  | ) |  |  |

inline

Returns the solution of the system Ax=b when A is triangular.

Parameters
:   |  |  |
    | --- | --- |
    | A | Triangular matrix. Specify upper or lower with TriView being Eigen::Upper or Eigen::Lower. |
    | b | Right hand side matrix or vector. |

Returns
:   x = A^-1 b, solution of the linear system.

Exceptions
:   |  |  |
    | --- | --- |
    | std::domain\_error | if A is not square or the rows of b don't match the size of A. |

Definition at line 27 of file mdivide\_left\_tri.hpp.

template<int TriView, typename T , int R1, int C1>

|  |  |  |  |  |  |
| --- | --- | --- | --- | --- | --- |
| Eigen::Matrix<T,R1,C1> stan::math::mdivide\_left\_tri | ( | const Eigen::Matrix< T, R1, C1 > & | *A* | ) |  |

inline

Returns the solution of the system Ax=b when A is triangular and b=I.

Parameters
:   |  |  |
    | --- | --- |
    | A | Triangular matrix. Specify upper or lower with TriView being Eigen::Upper or Eigen::Lower. |

Returns
:   x = A^-1 .

Exceptions
:   |  |  |
    | --- | --- |
    | std::domain\_error | if A is not square |

Definition at line 48 of file mdivide\_left\_tri.hpp.

template<typename T1 , typename T2 , int R1, int C1, int R2, int C2>

|  |  |  |  |
| --- | --- | --- | --- |
| Eigen::Matrix<typename boost::math::tools::promote\_args<T1,T2>::type, R1,C2> stan::math::mdivide\_left\_tri\_low | ( | const Eigen::Matrix< T1, R1, C1 > & | *A*, |
|  |  | const Eigen::Matrix< T2, R2, C2 > & | *b* |
|  | ) |  |  |

inline

Definition at line 16 of file mdivide\_left\_tri\_low.hpp.

template<typename T , int R1, int C1>

|  |  |  |  |  |  |
| --- | --- | --- | --- | --- | --- |
| Eigen::Matrix<T,R1,C1> stan::math::mdivide\_left\_tri\_low | ( | const Eigen::Matrix< T, R1, C1 > & | *A* | ) |  |

inline

Definition at line 31 of file mdivide\_left\_tri\_low.hpp.

template<typename T1 , typename T2 , int R1, int C1, int R2, int C2>

|  |  |  |  |
| --- | --- | --- | --- |
| Eigen::Matrix<typename boost::math::tools::promote\_args<T1,T2>::type,R1,C2> stan::math::mdivide\_right | ( | const Eigen::Matrix< T1, R1, C1 > & | *b*, |
|  |  | const Eigen::Matrix< T2, R2, C2 > & | *A* |
|  | ) |  |  |

inline

Returns the solution of the system Ax=b.

Parameters
:   |  |  |
    | --- | --- |
    | A | Matrix. |
    | b | Right hand side matrix or vector. |

Returns
:   x = b A^-1, solution of the linear system.

Exceptions
:   |  |  |
    | --- | --- |
    | std::domain\_error | if A is not square or the rows of b don't match the size of A. |

Definition at line 25 of file mdivide\_right.hpp.

template<typename T1 , typename T2 , int R1, int C1, int R2, int C2>

|  |  |  |  |
| --- | --- | --- | --- |
| Eigen::Matrix<typename boost::math::tools::promote\_args<T1,T2>::type,R1,C2> stan::math::mdivide\_right\_ldlt | ( | const Eigen::Matrix< T1, R1, C1 > & | *b*, |
|  |  | const stan::math::LDLT\_factor< T2, R2, C2 > & | *A* |
|  | ) |  |  |

inline

Returns the solution of the system xA=b given an LDLT\_factor of A.

Parameters
:   |  |  |
    | --- | --- |
    | A | LDLT\_factor |
    | b | Right hand side matrix or vector. |

Returns
:   x = A^-1 b, solution of the linear system.

Exceptions
:   |  |  |
    | --- | --- |
    | std::domain\_error | if rows of b don't match the size of A. |

Definition at line 25 of file mdivide\_right\_ldlt.hpp.

template<int R1, int C1, int R2, int C2>

|  |  |  |  |
| --- | --- | --- | --- |
| Eigen::Matrix<double,R1,C2> stan::math::mdivide\_right\_ldlt | ( | const Eigen::Matrix< double, R1, C1 > & | *b*, |
|  |  | const stan::math::LDLT\_factor< double, R2, C2 > & | *A* |
|  | ) |  |  |

inline

Definition at line 36 of file mdivide\_right\_ldlt.hpp.

template<typename T1 , typename T2 , int R1, int C1, int R2, int C2>

|  |  |  |  |
| --- | --- | --- | --- |
| Eigen::Matrix<typename boost::math::tools::promote\_args<T1,T2>::type,R1,C2> stan::math::mdivide\_right\_spd | ( | const Eigen::Matrix< T1, R1, C1 > & | *b*, |
|  |  | const Eigen::Matrix< T2, R2, C2 > & | *A* |
|  | ) |  |  |

inline

Returns the solution of the system Ax=b where A is symmetric positive definite.

Parameters
:   |  |  |
    | --- | --- |
    | A | Matrix. |
    | b | Right hand side matrix or vector. |

Returns
:   x = b A^-1, solution of the linear system.

Exceptions
:   |  |  |
    | --- | --- |
    | std::domain\_error | if A is not square or the rows of b don't match the size of A. |

Definition at line 28 of file mdivide\_right\_spd.hpp.

template<int TriView, typename T1 , typename T2 , int R1, int C1, int R2, int C2>

|  |  |  |  |
| --- | --- | --- | --- |
| Eigen::Matrix<typename boost::math::tools::promote\_args<T1,T2>::type,R1,C2> stan::math::mdivide\_right\_tri | ( | const Eigen::Matrix< T1, R1, C1 > & | *b*, |
|  |  | const Eigen::Matrix< T2, R2, C2 > & | *A* |
|  | ) |  |  |

inline

Returns the solution of the system Ax=b when A is triangular.

Parameters
:   |  |  |
    | --- | --- |
    | A | Triangular matrix. Specify upper or lower with TriView being Eigen::Upper or Eigen::Lower. |
    | b | Right hand side matrix or vector. |

Returns
:   x = b A^-1, solution of the linear system.

Exceptions
:   |  |  |
    | --- | --- |
    | std::domain\_error | if A is not square or the rows of b don't match the size of A. |

Definition at line 28 of file mdivide\_right\_tri.hpp.

template<typename T1 , typename T2 , int R1, int C1, int R2, int C2>

|  |  |  |  |
| --- | --- | --- | --- |
| Eigen::Matrix<typename boost::math::tools::promote\_args<T1,T2>::type,R1,C2> stan::math::mdivide\_right\_tri\_low | ( | const Eigen::Matrix< T1, R1, C1 > & | *b*, |
|  |  | const Eigen::Matrix< T2, R2, C2 > & | *A* |
|  | ) |  |  |

inline

Returns the solution of the system tri(A)x=b when tri(A) is a lower triangular view of the matrix A.

Parameters
:   |  |  |
    | --- | --- |
    | A | Matrix. |
    | b | Right hand side matrix or vector. |

Returns
:   x = b \* tri(A)^-1, solution of the linear system.

Exceptions
:   |  |  |
    | --- | --- |
    | std::domain\_error | if A is not square or the rows of b don't match the size of A. |

Definition at line 24 of file mdivide\_right\_tri\_low.hpp.

template<typename T >

|  |  |  |  |  |  |
| --- | --- | --- | --- | --- | --- |
| boost::math::tools::promote\_args<T>::type stan::math::mean | ( | const std::vector< T > & | *v* | ) |  |

inline

Returns the sample mean (i.e., average) of the coefficients in the specified standard vector.

Parameters
:   |  |  |
    | --- | --- |
    | v | Specified vector. |

Returns
:   Sample mean of vector coefficients.

Exceptions
:   |  |  |
    | --- | --- |
    | std::domain\_error | if the size of the vector is less than 1. |

Definition at line 23 of file mean.hpp.

template<typename T , int R, int C>

|  |  |  |  |  |  |
| --- | --- | --- | --- | --- | --- |
| boost::math::tools::promote\_args<T>::type stan::math::mean | ( | const Eigen::Matrix< T, R, C > & | *m* | ) |  |

inline

Returns the sample mean (i.e., average) of the coefficients in the specified vector, row vector, or matrix.

Parameters
:   |  |  |
    | --- | --- |
    | m | Specified vector, row vector, or matrix. |

Returns
:   Sample mean of vector coefficients.

Definition at line 40 of file mean.hpp.

|  |  |  |  |
| --- | --- | --- | --- |
| double stan::math::min | ( | const double | *a*, |
|  |  | const double | *b* |
|  | ) |  |  |

inline

Definition at line 7 of file min.hpp.

|  |  |  |  |  |  |
| --- | --- | --- | --- | --- | --- |
| int stan::math::min | ( | const std::vector< int > & | *x* | ) |  |

inline

Returns the minimum coefficient in the specified column vector.

Parameters
:   |  |  |
    | --- | --- |
    | x | Specified vector. |

Returns
:   Minimum coefficient value in the vector.

Template Parameters
:   |  |  |
    | --- | --- |
    | Type | of values being compared and returned |

Definition at line 18 of file min.hpp.

template<typename T >

|  |  |  |  |  |  |
| --- | --- | --- | --- | --- | --- |
| T stan::math::min | ( | const std::vector< T > & | *x* | ) |  |

inline

Returns the minimum coefficient in the specified column vector.

Parameters
:   |  |  |
    | --- | --- |
    | x | Specified vector. |

Returns
:   Minimum coefficient value in the vector.

Template Parameters
:   |  |  |
    | --- | --- |
    | Type | of values being compared and returned |

Definition at line 36 of file min.hpp.

template<typename T , int R, int C>

|  |  |  |  |  |  |
| --- | --- | --- | --- | --- | --- |
| T stan::math::min | ( | const Eigen::Matrix< T, R, C > & | *m* | ) |  |

inline

Returns the minimum coefficient in the specified matrix, vector, or row vector.

Parameters
:   |  |  |
    | --- | --- |
    | m | Specified matrix, vector, or row vector. |

Returns
:   Minimum coefficient value in the vector.

Definition at line 53 of file min.hpp.

template<typename T >

|  |  |  |  |  |  |
| --- | --- | --- | --- | --- | --- |
| T stan::math::minus | ( | const T & | *x* | ) |  |

inline

Returns the negation of the specified scalar or matrix.

Template Parameters
:   |  |  |
    | --- | --- |
    | T | Type of subtrahend. |

Parameters
:   |  |  |
    | --- | --- |
    | x | Subtrahend. |

Returns
:   Negation of subtrahend.

Definition at line 16 of file minus.hpp.

template<typename T2 >

|  |  |  |  |
| --- | --- | --- | --- |
| T2 stan::math::modified\_bessel\_first\_kind | ( | const int | *v*, |
|  |  | const T2 | *z* |
|  | ) |  |  |

inline

Definition at line 39 of file modified\_bessel\_first\_kind.hpp.

template<typename T2 >

|  |  |  |  |
| --- | --- | --- | --- |
| T2 stan::math::modified\_bessel\_second\_kind | ( | const int | *v*, |
|  |  | const T2 | *z* |
|  | ) |  |  |

inline

Definition at line 42 of file modified\_bessel\_second\_kind.hpp.

|  |  |  |  |
| --- | --- | --- | --- |
| int stan::math::modulus | ( | const int | *x*, |
|  |  | const int | *y* |
|  | ) |  |  |

inline

Definition at line 11 of file modulus.hpp.

template<int R, int C, typename T >

|  |  |  |  |
| --- | --- | --- | --- |
| boost::enable\_if\_c<boost::is\_arithmetic<T>::value, Eigen::Matrix<double, R, C> >::type stan::math::multiply | ( | const Eigen::Matrix< double, R, C > & | *m*, |
|  |  | T | *c* |
|  | ) |  |  |

inline

Return specified matrix multiplied by specified scalar.

Template Parameters
:   |  |  |
    | --- | --- |
    | R | Row type for matrix. |
    | C | Column type for matrix. |

Parameters
:   |  |  |
    | --- | --- |
    | m | Matrix. |
    | c | Scalar. |

Returns
:   Product of matrix and scalar.

Definition at line 25 of file multiply.hpp.

template<int R, int C, typename T >

|  |  |  |  |
| --- | --- | --- | --- |
| boost::enable\_if\_c<boost::is\_arithmetic<T>::value, Eigen::Matrix<double, R, C> >::type stan::math::multiply | ( | T | *c*, |
|  |  | const Eigen::Matrix< double, R, C > & | *m* |
|  | ) |  |  |

inline

Return specified scalar multiplied by specified matrix.

Template Parameters
:   |  |  |
    | --- | --- |
    | R | Row type for matrix. |
    | C | Column type for matrix. |

Parameters
:   |  |  |
    | --- | --- |
    | c | Scalar. |
    | m | Matrix. |

Returns
:   Product of scalar and matrix.

Definition at line 45 of file multiply.hpp.

template<int R1, int C1, int R2, int C2>

|  |  |  |  |
| --- | --- | --- | --- |
| Eigen::Matrix<double,R1,C2> stan::math::multiply | ( | const Eigen::Matrix< double, R1, C1 > & | *m1*, |
|  |  | const Eigen::Matrix< double, R2, C2 > & | *m2* |
|  | ) |  |  |

inline

Return the product of the specified matrices.

The number of columns in the first matrix must be the same as the number of rows in the second matrix.

Parameters
:   |  |  |
    | --- | --- |
    | m1 | First matrix. |
    | m2 | Second matrix. |

Returns
:   The product of the first and second matrices.

Exceptions
:   |  |  |
    | --- | --- |
    | std::domain\_error | if the number of columns of m1 does not match the number of rows of m2. |

Definition at line 61 of file multiply.hpp.

template<int C1, int R2>

|  |  |  |  |
| --- | --- | --- | --- |
| double stan::math::multiply | ( | const Eigen::Matrix< double, 1, C1 > & | *rv*, |
|  |  | const Eigen::Matrix< double, R2, 1 > & | *v* |
|  | ) |  |  |

inline

Return the scalar product of the specified row vector and specified column vector.

The return is the same as the dot product. The two vectors must be the same size.

Parameters
:   |  |  |
    | --- | --- |
    | rv | Row vector. |
    | v | Column vector. |

Returns
:   Scalar result of multiplying row vector by column vector.

Exceptions
:   |  |  |
    | --- | --- |
    | std::domain\_error | if rv and v are not the same size. |

Definition at line 79 of file multiply.hpp.

template<typename T\_a , typename T\_b >

|  |  |  |  |
| --- | --- | --- | --- |
| boost::math::tools::promote\_args<T\_a,T\_b>::type stan::math::multiply\_log | ( | const T\_a | *a*, |
|  |  | const T\_b | *b* |
|  | ) |  |  |

inline

Calculated the value of the first argument times log of the second argument while behaving properly with 0 inputs.

.

Parameters
:   |  |  |
    | --- | --- |
    | a | the first variable |
    | b | the second variable |

Returns
:   a \* log(b)

Definition at line 51 of file multiply\_log.hpp.

|  |  |  |  |  |  |
| --- | --- | --- | --- | --- | --- |
| matrix\_d stan::math::multiply\_lower\_tri\_self\_transpose | ( | const matrix\_d & | *L* | ) |  |

inline

Returns the result of multiplying the lower triangular portion of the input matrix by its own transpose.

Parameters
:   |  |  |
    | --- | --- |
    | L | Matrix to multiply. |

Returns
:   The lower triangular values in L times their own transpose.

Exceptions
:   |  |  |
    | --- | --- |
    | std::domain\_error | If the input matrix is not square. |

Definition at line 18 of file multiply\_lower\_tri\_self\_transpose.hpp.

|  |  |  |  |  |
| --- | --- | --- | --- | --- |
| double stan::math::negative\_infinity | ( |  | ) |  |

inline

Return negative infinity.

Returns
:   Negative infinity.

Definition at line 123 of file constants.hpp.

|  |  |  |  |  |
| --- | --- | --- | --- | --- |
| double stan::math::not\_a\_number | ( |  | ) |  |

inline

Return (quiet) not-a-number.

Returns
:   Quiet not-a-number.

Definition at line 132 of file constants.hpp.

template<typename T >

|  |  |  |  |  |  |
| --- | --- | --- | --- | --- | --- |
| int stan::math::num\_elements | ( | const T & | *x* | ) |  |

inline

Returns 1, the number of elements in a primitive type.

Parameters
:   |  |  |
    | --- | --- |
    | x | Argument of primitive type. |

Returns
:   1

Definition at line 19 of file num\_elements.hpp.

template<typename T , int R, int C>

|  |  |  |  |  |  |
| --- | --- | --- | --- | --- | --- |
| int stan::math::num\_elements | ( | const Eigen::Matrix< T, R, C > & | *m* | ) |  |

inline

Returns the size of the specified matrix.

Parameters
:   |  |  |
    | --- | --- |
    | m | argument matrix |

Returns
:   size of matrix

Definition at line 31 of file num\_elements.hpp.

template<typename T >

|  |  |  |  |  |  |
| --- | --- | --- | --- | --- | --- |
| int stan::math::num\_elements | ( | const std::vector< T > & | *v* | ) |  |

inline

Returns the number of elements in the specified vector.

This assumes it is not ragged and that each of its contained elements has the same number of elements.

Parameters
:   |  |  |
    | --- | --- |
    | m | argument vector |

Returns
:   number of contained arguments

Definition at line 45 of file num\_elements.hpp.

template<typename T1 , typename T2 >

|  |  |  |  |
| --- | --- | --- | --- |
| boost::math::tools::promote\_args<T1,T2>::type stan::math::owens\_t | ( | const T1 & | *h*, |
|  |  | const T2 & | *a* |
|  | ) |  |  |

inline

The Owen's T function of h and a.

Used to compute the cumulative density function for the skew normal distribution.

Template Parameters
:   |  |  |
    | --- | --- |
    | T1 | Type of first argument. |
    | T2 | Type of second argument. |

Parameters
:   |  |  |
    | --- | --- |
    | h | First argument |
    | a | Second argument |

Returns
:   The Owen's T function.

Definition at line 64 of file owens\_t.hpp.

template<typename T >

|  |  |  |  |  |  |
| --- | --- | --- | --- | --- | --- |
| boost::math::tools::promote\_args<T>::type stan::math::Phi | ( | const T | *x* | ) |  |

inline

The unit normal cumulative distribution function.

The return value for a specified input is the probability that a random unit normal variate is less than or equal to the specified value, defined by

This function can be used to implement the inverse link function for probit regression.

Phi will underflow to 0 below -37.5 and overflow to 1 above 8

Parameters
:   |  |  |
    | --- | --- |
    | x | Argument. |

Returns
:   Probability random sample is less than or equal to argument.

Definition at line 31 of file Phi.hpp.

template<typename T >

|  |  |  |  |  |  |
| --- | --- | --- | --- | --- | --- |
| boost::math::tools::promote\_args<T>::type stan::math::Phi\_approx | ( | T | *x* | ) |  |

inline

Approximation of the unit normal CDF.

http://www.jiem.org/index.php/jiem/article/download/60/27

This function can be used to implement the inverse link function for probit regression.

Parameters
:   |  |  |
    | --- | --- |
    | x | Argument. |

Returns
:   Probability random sample is less than or equal to argument.

Definition at line 23 of file Phi\_approx.hpp.

|  |  |  |  |  |
| --- | --- | --- | --- | --- |
| double stan::math::pi | ( |  | ) |  |

inline

Return the value of pi.

Returns
:   Pi.

Definition at line 77 of file constants.hpp.

|  |  |  |  |  |
| --- | --- | --- | --- | --- |
| double stan::math::positive\_infinity | ( |  | ) |  |

inline

Return positive infinity.

Returns
:   Positive infinity.

Definition at line 114 of file constants.hpp.

template<typename T >

|  |  |  |  |  |  |
| --- | --- | --- | --- | --- | --- |
| boost::enable\_if<boost::is\_arithmetic<T>, T>::type stan::math::primitive\_value | ( | T | *x* | ) |  |

inline

Return the value of the specified arithmetic argument unmodified with its own declared type.

This template function can only be instantiated with arithmetic types as defined by Boost's `is_arithmetic` trait metaprogram.

This function differs from `stan::math::value_of` in that it does not cast all return types to `double`.

Template Parameters
:   |  |  |
    | --- | --- |
    | T | type of arithmetic input. |

Parameters
:   |  |  |
    | --- | --- |
    | x | input. |

Returns
:   input unmodified.

Definition at line 30 of file primitive\_value.hpp.

template<typename T >

|  |  |  |  |  |  |
| --- | --- | --- | --- | --- | --- |
| boost::disable\_if<boost::is\_arithmetic<T>, double>::type stan::math::primitive\_value | ( | const T & | *x* | ) |  |

inline

Return the primitive value of the specified argument.

This implementation only applies to non-arithmetic types as defined by Boost's `is_arithmetic` trait metaprogram.

Template Parameters
:   |  |  |
    | --- | --- |
    | T | type of non-arithmetic input. |

Parameters
:   |  |  |
    | --- | --- |
    | x | input. |

Returns
:   value of input.

Definition at line 47 of file primitive\_value.hpp.

template<typename T >

|  |  |  |  |  |  |
| --- | --- | --- | --- | --- | --- |
| T stan::math::prod | ( | const std::vector< T > & | *v* | ) |  |

inline

Returns the product of the coefficients of the specified standard vector.

Parameters
:   |  |  |
    | --- | --- |
    | v | Specified vector. |

Returns
:   Product of coefficients of vector.

Definition at line 17 of file prod.hpp.

template<typename T , int R, int C>

|  |  |  |  |  |  |
| --- | --- | --- | --- | --- | --- |
| T stan::math::prod | ( | const Eigen::Matrix< T, R, C > & | *v* | ) |  |

inline

Returns the product of the coefficients of the specified column vector.

Parameters
:   |  |  |
    | --- | --- |
    | v | Specified vector. |

Returns
:   Product of coefficients of vector.

Definition at line 32 of file prod.hpp.

template<typename T1 , typename T2 , typename F >

|  |  |  |  |  |  |
| --- | --- | --- | --- | --- | --- |
| common\_type<T1,T2>::type stan::math::promote\_common | ( | const F & | *u* | ) |  |

inline

Definition at line 14 of file promote\_common.hpp.

template<typename T , typename S >

|  |  |  |  |  |  |
| --- | --- | --- | --- | --- | --- |
| promote\_scalar\_type<T,S>::type stan::math::promote\_scalar | ( | const S & | *x* | ) |  |

This is the top-level function to call to promote the scalar types of an input of type S to type T.

Template Parameters
:   |  |  |
    | --- | --- |
    | T | scalar type of output. |
    | S | input type. |

Parameters
:   |  |  |
    | --- | --- |
    | x | input vector. |

Returns
:   input vector with scalars promoted to type T.

Definition at line 103 of file promote\_scalar.hpp.

template<typename T >

|  |  |  |  |  |  |
| --- | --- | --- | --- | --- | --- |
| Eigen::Matrix<T,Eigen::Dynamic,Eigen::Dynamic> stan::math::qr\_Q | ( | const Eigen::Matrix< T, Eigen::Dynamic, Eigen::Dynamic > & | *m* | ) |  |

Definition at line 14 of file qr\_Q.hpp.

template<typename T >

|  |  |  |  |  |  |
| --- | --- | --- | --- | --- | --- |
| Eigen::Matrix<T,Eigen::Dynamic,Eigen::Dynamic> stan::math::qr\_R | ( | const Eigen::Matrix< T, Eigen::Dynamic, Eigen::Dynamic > & | *m* | ) |  |

Definition at line 14 of file qr\_R.hpp.

template<int RA, int CA, int RB, int CB, typename T >

|  |  |  |  |
| --- | --- | --- | --- |
| Eigen::Matrix<T,CB,CB> stan::math::quad\_form | ( | const Eigen::Matrix< T, RA, CA > & | *A*, |
|  |  | const Eigen::Matrix< T, RB, CB > & | *B* |
|  | ) |  |  |

inline

Compute B^T A B.

Definition at line 21 of file quad\_form.hpp.

template<int RA, int CA, int RB, typename T >

|  |  |  |  |
| --- | --- | --- | --- |
| T stan::math::quad\_form | ( | const Eigen::Matrix< T, RA, CA > & | *A*, |
|  |  | const Eigen::Matrix< T, RB, 1 > & | *B* |
|  | ) |  |  |

inline

Definition at line 33 of file quad\_form.hpp.

template<typename T1 , typename T2 , int R, int C>

|  |  |  |  |
| --- | --- | --- | --- |
| Matrix<typename promote\_args<T1,T2>::type, Dynamic, Dynamic> stan::math::quad\_form\_diag | ( | const Matrix< T1, Dynamic, Dynamic > & | *mat*, |
|  |  | const Matrix< T2, R, C > & | *vec* |
|  | ) |  |  |

inline

Definition at line 20 of file quad\_form\_diag.hpp.

template<int RA, int CA, int RB, int CB, typename T >

|  |  |  |  |
| --- | --- | --- | --- |
| Eigen::Matrix<T,CB,CB> stan::math::quad\_form\_sym | ( | const Eigen::Matrix< T, RA, CA > & | *A*, |
|  |  | const Eigen::Matrix< T, RB, CB > & | *B* |
|  | ) |  |  |

inline

Definition at line 47 of file quad\_form.hpp.

template<int RA, int CA, int RB, typename T >

|  |  |  |  |
| --- | --- | --- | --- |
| T stan::math::quad\_form\_sym | ( | const Eigen::Matrix< T, RA, CA > & | *A*, |
|  |  | const Eigen::Matrix< T, RB, 1 > & | *B* |
|  | ) |  |  |

inline

Definition at line 62 of file quad\_form.hpp.

template<typename T >

|  |  |  |  |
| --- | --- | --- | --- |
| size\_t stan::math::rank | ( | const std::vector< T > & | *v*, |
|  |  | int | *s* |
|  | ) |  |  |

inline

Return the number of components of v less than v[s].

Returns
:   Number of components of v less than v[s].

Template Parameters
:   |  |  |
    | --- | --- |
    | T | Type of elements of the vector. |

Definition at line 18 of file rank.hpp.

template<typename T , int R, int C>

|  |  |  |  |
| --- | --- | --- | --- |
| size\_t stan::math::rank | ( | const Eigen::Matrix< T, R, C > & | *v*, |
|  |  | int | *s* |
|  | ) |  |  |

inline

Return the number of components of v less than v[s].

Returns
:   Number of components of v less than v[s].

Template Parameters
:   |  |  |
    | --- | --- |
    | T | Type of elements of the vector. |

Definition at line 36 of file rank.hpp.

template<typename T >

|  |  |  |  |
| --- | --- | --- | --- |
| std::vector<T> stan::math::rep\_array | ( | const T & | *x*, |
|  |  | int | *n* |
|  | ) |  |  |

inline

Definition at line 14 of file rep\_array.hpp.

template<typename T >

|  |  |  |  |
| --- | --- | --- | --- |
| std::vector<std::vector<T> > stan::math::rep\_array | ( | const T & | *x*, |
|  |  | int | *m*, |
|  |  | int | *n* |
|  | ) |  |  |

inline

Definition at line 21 of file rep\_array.hpp.

template<typename T >

|  |  |  |  |
| --- | --- | --- | --- |
| std::vector<std::vector<std::vector<T> > > stan::math::rep\_array | ( | const T & | *x*, |
|  |  | int | *k*, |
|  |  | int | *m*, |
|  |  | int | *n* |
|  | ) |  |  |

inline

Definition at line 30 of file rep\_array.hpp.

template<typename T >

|  |  |  |  |
| --- | --- | --- | --- |
| Eigen::Matrix<typename boost::math::tools::promote\_args<T>::type, Eigen::Dynamic,Eigen::Dynamic> stan::math::rep\_matrix | ( | const T & | *x*, |
|  |  | int | *m*, |
|  |  | int | *n* |
|  | ) |  |  |

inline

Definition at line 16 of file rep\_matrix.hpp.

template<typename T >

|  |  |  |  |
| --- | --- | --- | --- |
| Eigen::Matrix<T,Eigen::Dynamic,Eigen::Dynamic> stan::math::rep\_matrix | ( | const Eigen::Matrix< T, Eigen::Dynamic, 1 > & | *v*, |
|  |  | int | *n* |
|  | ) |  |  |

inline

Definition at line 25 of file rep\_matrix.hpp.

template<typename T >

|  |  |  |  |
| --- | --- | --- | --- |
| Eigen::Matrix<T,Eigen::Dynamic,Eigen::Dynamic> stan::math::rep\_matrix | ( | const Eigen::Matrix< T, 1, Eigen::Dynamic > & | *rv*, |
|  |  | int | *m* |
|  | ) |  |  |

inline

Definition at line 34 of file rep\_matrix.hpp.

template<typename T >

|  |  |  |  |
| --- | --- | --- | --- |
| Eigen::Matrix<typename boost::math::tools::promote\_args<T>::type, 1,Eigen::Dynamic> stan::math::rep\_row\_vector | ( | const T & | *x*, |
|  |  | int | *m* |
|  | ) |  |  |

inline

Definition at line 15 of file rep\_row\_vector.hpp.

template<typename T >

|  |  |  |  |
| --- | --- | --- | --- |
| Eigen::Matrix<typename boost::math::tools::promote\_args<T>::type, Eigen::Dynamic,1> stan::math::rep\_vector | ( | const T & | *x*, |
|  |  | int | *n* |
|  | ) |  |  |

inline

Definition at line 16 of file rep\_vector.hpp.

template<typename T >

|  |  |  |  |
| --- | --- | --- | --- |
| void stan::math::resize | ( | T & | *x*, |
|  |  | std::vector< size\_t > | *dims* |
|  | ) |  |  |

inline

Recursively resize the specified vector of vectors, which must bottom out at scalar values, Eigen vectors or Eigen matrices.

Parameters
:   |  |  |
    | --- | --- |
    | x | Array-like object to resize. |
    | dims | New dimensions. |

Template Parameters
:   |  |  |
    | --- | --- |
    | T | Type of object being resized. |

Definition at line 63 of file resize.hpp.

template<typename T1 , typename T2 >

|  |  |  |  |
| --- | --- | --- | --- |
| boost::math::tools::promote\_args<T1,T2>::type stan::math::rising\_factorial | ( | const T1 | *x*, |
|  |  | const T2 | *n* |
|  | ) |  |  |

inline

Definition at line 53 of file rising\_factorial.hpp.

template<typename T >

|  |  |  |  |
| --- | --- | --- | --- |
| Eigen::Matrix<T,1,Eigen::Dynamic> stan::math::row | ( | const Eigen::Matrix< T, Eigen::Dynamic, Eigen::Dynamic > & | *m*, |
|  |  | size\_t | *i* |
|  | ) |  |  |

inline

Return the specified row of the specified matrix, using start-at-1 indexing.

This is equivalent to calling `m.row(i - 1)` and assigning the resulting template expression to a row vector.

Template Parameters
:   |  |  |
    | --- | --- |
    | T | Scalar value type for matrix. |

Parameters
:   |  |  |
    | --- | --- |
    | m | Matrix. |
    | i | Row index (count from 1). |

Returns
:   Specified row of the matrix.

Definition at line 26 of file row.hpp.

template<typename T , int R, int C>

|  |  |  |  |  |  |
| --- | --- | --- | --- | --- | --- |
| size\_t stan::math::rows | ( | const Eigen::Matrix< T, R, C > & | *m* | ) |  |

inline

Definition at line 12 of file rows.hpp.

template<int R1, int C1, int R2, int C2>

|  |  |  |  |
| --- | --- | --- | --- |
| Eigen::Matrix<double, R1, 1> stan::math::rows\_dot\_product | ( | const Eigen::Matrix< double, R1, C1 > & | *v1*, |
|  |  | const Eigen::Matrix< double, R2, C2 > & | *v2* |
|  | ) |  |  |

inline

Returns the dot product of the specified vectors.

Parameters
:   |  |  |
    | --- | --- |
    | v1 | First vector. |
    | v2 | Second vector. |

Returns
:   Dot product of the vectors.

Exceptions
:   |  |  |
    | --- | --- |
    | std::domain\_error | If the vectors are not the same size or if they are both not vector dimensioned. |

Definition at line 22 of file rows\_dot\_product.hpp.

template<typename T , int R, int C>

|  |  |  |  |  |  |
| --- | --- | --- | --- | --- | --- |
| Eigen::Matrix<T,R,1> stan::math::rows\_dot\_self | ( | const Eigen::Matrix< T, R, C > & | *x* | ) |  |

inline

Returns the dot product of each row of a matrix with itself.

Parameters
:   |  |  |
    | --- | --- |
    | x | Matrix. |

Template Parameters
:   |  |  |
    | --- | --- |
    | T | scalar type |

Definition at line 16 of file rows\_dot\_self.hpp.

|  |  |  |  |
| --- | --- | --- | --- |
| void stan::math::scaled\_add | ( | std::vector< double > & | *x*, |
|  |  | const std::vector< double > & | *y*, |
|  |  | const double | *lambda* |
|  | ) |  |  |

inline

Definition at line 11 of file scaled\_add.hpp.

template<typename T >

|  |  |  |  |  |  |
| --- | --- | --- | --- | --- | --- |
| boost::math::tools::promote\_args<T>::type stan::math::sd | ( | const std::vector< T > & | *v* | ) |  |

inline

Returns the unbiased sample standard deviation of the coefficients in the specified column vector.

Parameters
:   |  |  |
    | --- | --- |
    | v | Specified vector. |

Returns
:   Sample variance of vector.

Definition at line 22 of file sd.hpp.

template<typename T , int R, int C>

|  |  |  |  |  |  |
| --- | --- | --- | --- | --- | --- |
| boost::math::tools::promote\_args<T>::type stan::math::sd | ( | const Eigen::Matrix< T, R, C > & | *m* | ) |  |

inline

Returns the unbiased sample standard deviation of the coefficients in the specified vector, row vector, or matrix.

Parameters
:   |  |  |
    | --- | --- |
    | m | Specified vector, row vector or matrix. |

Returns
:   Sample variance.

Definition at line 37 of file sd.hpp.

template<typename T >

|  |  |  |  |
| --- | --- | --- | --- |
| Eigen::Matrix<T,Eigen::Dynamic,1> stan::math::segment | ( | const Eigen::Matrix< T, Eigen::Dynamic, 1 > & | *v*, |
|  |  | size\_t | *i*, |
|  |  | size\_t | *n* |
|  | ) |  |  |

inline

Return the specified number of elements as a vector starting from the specified element - 1 of the specified vector.

Definition at line 20 of file segment.hpp.

template<typename T >

|  |  |  |  |
| --- | --- | --- | --- |
| Eigen::Matrix<T,1,Eigen::Dynamic> stan::math::segment | ( | const Eigen::Matrix< T, 1, Eigen::Dynamic > & | *v*, |
|  |  | size\_t | *i*, |
|  |  | size\_t | *n* |
|  | ) |  |  |

inline

Definition at line 34 of file segment.hpp.

template<typename T >

|  |  |  |  |
| --- | --- | --- | --- |
| std::vector<T> stan::math::segment | ( | const std::vector< T > & | *sv*, |
|  |  | size\_t | *i*, |
|  |  | size\_t | *n* |
|  | ) |  |  |

Definition at line 49 of file segment.hpp.

template<typename T >

|  |  |  |  |  |  |
| --- | --- | --- | --- | --- | --- |
| int stan::math::sign | ( | const T & | *z* | ) |  |

inline

Definition at line 9 of file sign.hpp.

template<typename T >

|  |  |  |  |  |  |
| --- | --- | --- | --- | --- | --- |
| Eigen::Matrix<T,Eigen::Dynamic,1> stan::math::singular\_values | ( | const Eigen::Matrix< T, Eigen::Dynamic, Eigen::Dynamic > & | *m* | ) |  |

Return the vector of the singular values of the specified matrix in decreasing order of magnitude.

See the documentation for `svd()` for information on the signular values.

Parameters
:   |  |  |
    | --- | --- |
    | m | Specified matrix. |

Returns
:   Singular values of the matrix.

Definition at line 19 of file singular\_values.hpp.

template<typename T >

|  |  |  |  |  |  |
| --- | --- | --- | --- | --- | --- |
| int stan::math::size | ( | const std::vector< T > & | *x* | ) |  |

inline

Definition at line 11 of file size.hpp.

template<typename T >

|  |  |  |  |  |  |
| --- | --- | --- | --- | --- | --- |
| Eigen::Matrix<T,Eigen::Dynamic,1> stan::math::softmax | ( | const Eigen::Matrix< T, Eigen::Dynamic, 1 > & | *v* | ) |  |

inline

Return the softmax of the specified vector.

The entries in the Jacobian of the softmax function are given by

Template Parameters
:   |  |  |
    | --- | --- |
    | T | Scalar type of values in vector. |

Parameters
:   |  |  |  |
    | --- | --- | --- |
    | [in] | v | Vector to transform. |

Returns
:   Unit simplex result of the softmax transform of the vector.

Definition at line 46 of file softmax.hpp.

template<typename T >

|  |  |  |  |  |  |
| --- | --- | --- | --- | --- | --- |
| std::vector<T> stan::math::sort\_asc | ( | std::vector< T > | *xs* | ) |  |

inline

Return the specified standard vector in ascending order.

Parameters
:   |  |  |
    | --- | --- |
    | xs | Standard vector to order. |

Returns
:   Standard vector ordered.

Template Parameters
:   |  |  |
    | --- | --- |
    | T | Type of elements of the vector. |

Definition at line 20 of file sort.hpp.

template<typename T , int R, int C>

|  |  |  |  |  |  |
| --- | --- | --- | --- | --- | --- |
| Eigen::Matrix<T,R,C> stan::math::sort\_asc | ( | Eigen::Matrix< T, R, C > | *xs* | ) |  |

inline

Return the specified eigen vector in ascending order.

Parameters
:   |  |  |
    | --- | --- |
    | xs | Eigen vector to order. |

Returns
:   Eigen vector ordered.

Template Parameters
:   |  |  |
    | --- | --- |
    | T | Type of elements of the vector. |

Definition at line 46 of file sort.hpp.

template<typename T >

|  |  |  |  |  |  |
| --- | --- | --- | --- | --- | --- |
| std::vector<T> stan::math::sort\_desc | ( | std::vector< T > | *xs* | ) |  |

inline

Return the specified standard vector in descending order.

Parameters
:   |  |  |
    | --- | --- |
    | xs | Standard vector to order. |

Returns
:   Standard vector ordered.

Template Parameters
:   |  |  |
    | --- | --- |
    | T | Type of elements of the vector. |

Definition at line 33 of file sort.hpp.

template<typename T , int R, int C>

|  |  |  |  |  |  |
| --- | --- | --- | --- | --- | --- |
| Eigen::Matrix<T,R,C> stan::math::sort\_desc | ( | Eigen::Matrix< T, R, C > | *xs* | ) |  |

inline

Return the specified eigen vector in descending order.

Parameters
:   |  |  |
    | --- | --- |
    | xs | Eigen vector to order. |

Returns
:   Eigen vector ordered.

Template Parameters
:   |  |  |
    | --- | --- |
    | T | Type of elements of the vector. |

Definition at line 59 of file sort.hpp.

template<typename C >

|  |  |  |  |  |  |
| --- | --- | --- | --- | --- | --- |
| std::vector<int> stan::math::sort\_indices\_asc | ( | const C & | *xs* | ) |  |

Return a sorted copy of the argument container in ascending order.

Template Parameters
:   |  |  |
    | --- | --- |
    | C | type of container |

Parameters
:   |  |  |
    | --- | --- |
    | xs | Container to sort |

Returns
:   sorted version of container

Definition at line 86 of file sort\_indices.hpp.

template<typename C >

|  |  |  |  |  |  |
| --- | --- | --- | --- | --- | --- |
| std::vector<int> stan::math::sort\_indices\_desc | ( | const C & | *xs* | ) |  |

Return a sorted copy of the argument container in ascending order.

Template Parameters
:   |  |  |
    | --- | --- |
    | C | type of container |

Parameters
:   |  |  |
    | --- | --- |
    | xs | Container to sort |

Returns
:   sorted version of container

Definition at line 98 of file sort\_indices.hpp.

|  |  |  |  |  |
| --- | --- | --- | --- | --- |
| double stan::math::sqrt2 | ( |  | ) |  |

inline

Return the square root of two.

Returns
:   Square root of two.

Definition at line 95 of file constants.hpp.

template<typename T >

|  |  |  |  |  |  |
| --- | --- | --- | --- | --- | --- |
| T stan::math::square | ( | const T | *x* | ) |  |

inline

Return the square of the specified argument.

.

The implementation of `square(x)` is just `x * x`. Given this, this method is mainly useful in cases where `x` is not a simple primitive type, particularly when it is an auto-dif type.

Parameters
:   |  |  |
    | --- | --- |
    | x | Input to square. |

Returns
:   Square of input.

Template Parameters
:   |  |  |
    | --- | --- |
    | T | Type of scalar. |

Definition at line 22 of file square.hpp.

template<int R1, int C1, int R2, int C2, typename T1 , typename T2 >

|  |  |  |  |
| --- | --- | --- | --- |
| boost::math::tools::promote\_args<T1,T2>::type stan::math::squared\_distance | ( | const Eigen::Matrix< T1, R1, C1 > & | *v1*, |
|  |  | const Eigen::Matrix< T2, R2, C2 > & | *v2* |
|  | ) |  |  |

inline

Returns the squared distance between the specified vectors.

Parameters
:   |  |  |
    | --- | --- |
    | v1 | First vector. |
    | v2 | Second vector. |

Returns
:   Dot product of the vectors.

Exceptions
:   |  |  |
    | --- | --- |
    | std::domain\_error | If the vectors are not the same size or if they are both not vector dimensioned. |

Definition at line 22 of file squared\_distance.hpp.

template<typename T >

|  |  |  |  |
| --- | --- | --- | --- |
| void stan::math::stan\_print | ( | std::ostream \* | *o*, |
|  |  | const T & | *x* |
|  | ) |  |  |

Definition at line 12 of file stan\_print.hpp.

template<typename T >

|  |  |  |  |
| --- | --- | --- | --- |
| void stan::math::stan\_print | ( | std::ostream \* | *o*, |
|  |  | const std::vector< T > & | *x* |
|  | ) |  |  |

Definition at line 17 of file stan\_print.hpp.

template<typename T >

|  |  |  |  |
| --- | --- | --- | --- |
| void stan::math::stan\_print | ( | std::ostream \* | *o*, |
|  |  | const Eigen::Matrix< T, Eigen::Dynamic, 1 > & | *x* |
|  | ) |  |  |

Definition at line 27 of file stan\_print.hpp.

template<typename T >

|  |  |  |  |
| --- | --- | --- | --- |
| void stan::math::stan\_print | ( | std::ostream \* | *o*, |
|  |  | const Eigen::Matrix< T, 1, Eigen::Dynamic > & | *x* |
|  | ) |  |  |

Definition at line 37 of file stan\_print.hpp.

template<typename T >

|  |  |  |  |
| --- | --- | --- | --- |
| void stan::math::stan\_print | ( | std::ostream \* | *o*, |
|  |  | const Eigen::Matrix< T, Eigen::Dynamic, Eigen::Dynamic > & | *x* |
|  | ) |  |  |

Definition at line 47 of file stan\_print.hpp.

template<typename T >

|  |  |  |  |  |  |
| --- | --- | --- | --- | --- | --- |
| int stan::math::step | ( | const T | *y* | ) |  |

inline

The step, or Heaviside, function.

The function is defined by

`step(y) = (y < 0.0) ? 0 : 1`.

Parameters
:   |  |  |
    | --- | --- |
    | y | Scalar argument. |

Returns
:   1 if the specified argument is greater than or equal to 0.0, and 0 otherwise.

Definition at line 29 of file step.hpp.

|  |  |  |  |
| --- | --- | --- | --- |
| void stan::math::sub | ( | std::vector< double > & | *x*, |
|  |  | std::vector< double > & | *y*, |
|  |  | std::vector< double > & | *result* |
|  | ) |  |  |

inline

Definition at line 10 of file sub.hpp.

template<typename T >

|  |  |  |  |
| --- | --- | --- | --- |
| Eigen::Matrix<T,Eigen::Dynamic,1> stan::math::sub\_col | ( | const Eigen::Matrix< T, Eigen::Dynamic, Eigen::Dynamic > & | *m*, |
|  |  | size\_t | *i*, |
|  |  | size\_t | *j*, |
|  |  | size\_t | *nrows* |
|  | ) |  |  |

inline

Return a nrows x 1 subcolumn starting at (i-1,j-1).

Parameters
:   |  |  |
    | --- | --- |
    | m | Matrix |
    | i | Starting row + 1 |
    | j | Starting column + 1 |
    | nrows | Number of rows in block |

Definition at line 22 of file sub\_col.hpp.

template<typename T >

|  |  |  |  |
| --- | --- | --- | --- |
| Eigen::Matrix<T,1,Eigen::Dynamic> stan::math::sub\_row | ( | const Eigen::Matrix< T, Eigen::Dynamic, Eigen::Dynamic > & | *m*, |
|  |  | size\_t | *i*, |
|  |  | size\_t | *j*, |
|  |  | size\_t | *ncols* |
|  | ) |  |  |

inline

Return a 1 x nrows subrow starting at (i-1,j-1).

Parameters
:   |  |  |
    | --- | --- |
    | m | Matrix |
    | i | Starting row + 1 |
    | j | Starting column + 1 |
    | ncols | Number of columns in block |

Definition at line 23 of file sub\_row.hpp.

template<typename T1 , typename T2 , int R, int C>

|  |  |  |  |
| --- | --- | --- | --- |
| Eigen::Matrix<typename boost::math::tools::promote\_args<T1,T2>::type, R, C> stan::math::subtract | ( | const Eigen::Matrix< T1, R, C > & | *m1*, |
|  |  | const Eigen::Matrix< T2, R, C > & | *m2* |
|  | ) |  |  |

inline

Return the result of subtracting the second specified matrix from the first specified matrix.

The return scalar type is the promotion of the input types.

Template Parameters
:   |  |  |
    | --- | --- |
    | T1 | Scalar type of first matrix. |
    | T2 | Scalar type of second matrix. |
    | R | Row type of matrices. |
    | C | Column type of matrices. |

Parameters
:   |  |  |
    | --- | --- |
    | m1 | First matrix. |
    | m2 | Second matrix. |

Returns
:   Difference between first matrix and second matrix.

Definition at line 27 of file subtract.hpp.

template<typename T1 , typename T2 , int R, int C>

|  |  |  |  |
| --- | --- | --- | --- |
| Eigen::Matrix<typename boost::math::tools::promote\_args<T1,T2>::type, R, C> stan::math::subtract | ( | const T1 & | *c*, |
|  |  | const Eigen::Matrix< T2, R, C > & | *m* |
|  | ) |  |  |

inline

Definition at line 41 of file subtract.hpp.

template<typename T1 , typename T2 , int R, int C>

|  |  |  |  |
| --- | --- | --- | --- |
| Eigen::Matrix<typename boost::math::tools::promote\_args<T1,T2>::type, R, C> stan::math::subtract | ( | const Eigen::Matrix< T1, R, C > & | *m*, |
|  |  | const T2 & | *c* |
|  | ) |  |  |

inline

Definition at line 53 of file subtract.hpp.

|  |  |  |  |  |  |
| --- | --- | --- | --- | --- | --- |
| double stan::math::sum | ( | std::vector< double > & | *x* | ) |  |

inline

Definition at line 10 of file sum.hpp.

template<typename T >

|  |  |  |  |  |  |
| --- | --- | --- | --- | --- | --- |
| T stan::math::sum | ( | const std::vector< T > & | *xs* | ) |  |

inline

Return the sum of the values in the specified standard vector.

Parameters
:   |  |  |
    | --- | --- |
    | xs | Standard vector to sum. |

Returns
:   Sum of elements.

Template Parameters
:   |  |  |
    | --- | --- |
    | T | Type of elements summed. |

Definition at line 19 of file sum.hpp.

template<typename T , int R, int C>

|  |  |  |  |  |  |
| --- | --- | --- | --- | --- | --- |
| double stan::math::sum | ( | const Eigen::Matrix< T, R, C > & | *v* | ) |  |

inline

Returns the sum of the coefficients of the specified column vector.

Parameters
:   |  |  |
    | --- | --- |
    | v | Specified vector. |

Returns
:   Sum of coefficients of vector.

Definition at line 34 of file sum.hpp.

template<typename T >

|  |  |  |  |
| --- | --- | --- | --- |
| Eigen::Matrix<T,Eigen::Dynamic,1> stan::math::tail | ( | const Eigen::Matrix< T, Eigen::Dynamic, 1 > & | *v*, |
|  |  | size\_t | *n* |
|  | ) |  |  |

inline

Return the specified number of elements as a vector from the back of the specified vector.

Definition at line 22 of file tail.hpp.

template<typename T >

|  |  |  |  |
| --- | --- | --- | --- |
| Eigen::Matrix<T,1,Eigen::Dynamic> stan::math::tail | ( | const Eigen::Matrix< T, 1, Eigen::Dynamic > & | *rv*, |
|  |  | size\_t | *n* |
|  | ) |  |  |

inline

Return the specified number of elements as a row vector from the back of the specified row vector.

Definition at line 37 of file tail.hpp.

template<typename T >

|  |  |  |  |
| --- | --- | --- | --- |
| std::vector<T> stan::math::tail | ( | const std::vector< T > & | *sv*, |
|  |  | size\_t | *n* |
|  | ) |  |  |

Definition at line 45 of file tail.hpp.

|  |  |  |  |  |  |
| --- | --- | --- | --- | --- | --- |
| matrix\_d stan::math::tcrossprod | ( | const matrix\_d & | *M* | ) |  |

inline

Returns the result of post-multiplying a matrix by its own transpose.

Parameters
:   |  |  |
    | --- | --- |
    | M | Matrix to multiply. |

Returns
:   M times its transpose.

Definition at line 17 of file tcrossprod.hpp.

template<typename T , int R, int C>

|  |  |  |  |  |  |
| --- | --- | --- | --- | --- | --- |
| vector<T> stan::math::to\_array\_1d | ( | const Matrix< T, R, C > & | *matrix* | ) |  |

inline

Definition at line 131 of file containers\_conversion.hpp.

template<typename T >

|  |  |  |  |  |  |
| --- | --- | --- | --- | --- | --- |
| vector<T> stan::math::to\_array\_1d | ( | const vector< T > & | *x* | ) |  |

inline

Definition at line 143 of file containers\_conversion.hpp.

template<typename T >

|  |  |  |  |  |  |
| --- | --- | --- | --- | --- | --- |
| vector<typename scalar\_type<T>::type> stan::math::to\_array\_1d | ( | const vector< vector< T > > & | *x* | ) |  |

inline

Definition at line 150 of file containers\_conversion.hpp.

template<typename T >

|  |  |  |  |  |  |
| --- | --- | --- | --- | --- | --- |
| vector< vector<T> > stan::math::to\_array\_2d | ( | const Matrix< T, Dynamic, Dynamic > & | *matrix* | ) |  |

inline

Definition at line 116 of file containers\_conversion.hpp.

template<typename T , int R, int C>

|  |  |  |  |  |  |
| --- | --- | --- | --- | --- | --- |
| Matrix<T, Dynamic, Dynamic> stan::math::to\_matrix | ( | Matrix< T, R, C > | *matrix* | ) |  |

inline

Definition at line 20 of file containers\_conversion.hpp.

template<typename T >

|  |  |  |  |  |  |
| --- | --- | --- | --- | --- | --- |
| Matrix<T, Dynamic, Dynamic> stan::math::to\_matrix | ( | const vector< vector< T > > & | *vec* | ) |  |

inline

Definition at line 27 of file containers\_conversion.hpp.

|  |  |  |  |  |  |
| --- | --- | --- | --- | --- | --- |
| Matrix<double, Dynamic, Dynamic> stan::math::to\_matrix | ( | const vector< vector< int > > & | *vec* | ) |  |

inline

Definition at line 44 of file containers\_conversion.hpp.

template<typename T , int R, int C>

|  |  |  |  |  |  |
| --- | --- | --- | --- | --- | --- |
| Matrix<T, 1, Dynamic> stan::math::to\_row\_vector | ( | const Matrix< T, R, C > & | *matrix* | ) |  |

inline

Definition at line 91 of file containers\_conversion.hpp.

template<typename T >

|  |  |  |  |  |  |
| --- | --- | --- | --- | --- | --- |
| Matrix<T, 1, Dynamic> stan::math::to\_row\_vector | ( | const vector< T > & | *vec* | ) |  |

inline

Definition at line 98 of file containers\_conversion.hpp.

|  |  |  |  |  |  |
| --- | --- | --- | --- | --- | --- |
| Matrix<double, 1, Dynamic> stan::math::to\_row\_vector | ( | const vector< int > & | *vec* | ) |  |

inline

Definition at line 104 of file containers\_conversion.hpp.

template<typename T , int R, int C>

|  |  |  |  |  |  |
| --- | --- | --- | --- | --- | --- |
| Matrix<T, Dynamic, 1> stan::math::to\_vector | ( | const Matrix< T, R, C > & | *matrix* | ) |  |

inline

Definition at line 64 of file containers\_conversion.hpp.

template<typename T >

|  |  |  |  |  |  |
| --- | --- | --- | --- | --- | --- |
| Matrix<T, Dynamic, 1> stan::math::to\_vector | ( | const vector< T > & | *vec* | ) |  |

inline

Definition at line 71 of file containers\_conversion.hpp.

|  |  |  |  |  |  |
| --- | --- | --- | --- | --- | --- |
| Matrix<double, Dynamic, 1> stan::math::to\_vector | ( | const vector< int > & | *vec* | ) |  |

inline

Definition at line 77 of file containers\_conversion.hpp.

template<typename T >

|  |  |  |  |  |  |
| --- | --- | --- | --- | --- | --- |
| T stan::math::trace | ( | const Eigen::Matrix< T, Eigen::Dynamic, Eigen::Dynamic > & | *m* | ) |  |

inline

Returns the trace of the specified matrix.

The trace is defined as the sum of the elements on the diagonal. The matrix is not required to be square. Returns 0 if matrix is empty.

Parameters
:   |  |  |  |
    | --- | --- | --- |
    | [in] | m | Specified matrix. |

Returns
:   Trace of the matrix.

Definition at line 20 of file trace.hpp.

template<typename T >

|  |  |  |  |  |  |
| --- | --- | --- | --- | --- | --- |
| T stan::math::trace | ( | const T & | *m* | ) |  |

inline

Definition at line 26 of file trace.hpp.

template<typename T1 , typename T2 , typename T3 , int R1, int C1, int R2, int C2, int R3, int C3>

|  |  |  |  |
| --- | --- | --- | --- |
| boost::enable\_if\_c<!stan::is\_var<T1>::value && !stan::is\_var<T2>::value && !stan::is\_var<T3>::value, typename boost::math::tools::promote\_args<T1,T2,T3>::type>::type stan::math::trace\_gen\_inv\_quad\_form\_ldlt | ( | const Eigen::Matrix< T1, R1, C1 > & | *D*, |
|  |  | const stan::math::LDLT\_factor< T2, R2, C2 > & | *A*, |
|  |  | const Eigen::Matrix< T3, R3, C3 > & | *B* |
|  | ) |  |  |

inline

Definition at line 28 of file trace\_gen\_inv\_quad\_form\_ldlt.hpp.

template<int RD, int CD, int RA, int CA, int RB, int CB>

|  |  |  |  |
| --- | --- | --- | --- |
| double stan::math::trace\_gen\_quad\_form | ( | const Eigen::Matrix< double, RD, CD > & | *D*, |
|  |  | const Eigen::Matrix< double, RA, CA > & | *A*, |
|  |  | const Eigen::Matrix< double, RB, CB > & | *B* |
|  | ) |  |  |

inline

Compute trace(D B^T A B).

Definition at line 17 of file trace\_gen\_quad\_form.hpp.

template<typename T1 , typename T2 , int R2, int C2, int R3, int C3>

|  |  |  |  |
| --- | --- | --- | --- |
| boost::enable\_if\_c<!stan::is\_var<T1>::value && !stan::is\_var<T2>::value, typename boost::math::tools::promote\_args<T1,T2>::type>::type stan::math::trace\_inv\_quad\_form\_ldlt | ( | const stan::math::LDLT\_factor< T1, R2, C2 > & | *A*, |
|  |  | const Eigen::Matrix< T2, R3, C3 > & | *B* |
|  | ) |  |  |

inline

Definition at line 25 of file trace\_inv\_quad\_form\_ldlt.hpp.

template<int RA, int CA, int RB, int CB>

|  |  |  |  |
| --- | --- | --- | --- |
| double stan::math::trace\_quad\_form | ( | const Eigen::Matrix< double, RA, CA > & | *A*, |
|  |  | const Eigen::Matrix< double, RB, CB > & | *B* |
|  | ) |  |  |

inline

Compute trace(B^T A B).

Definition at line 17 of file trace\_quad\_form.hpp.

template<typename T , int R, int C>

|  |  |  |  |  |  |
| --- | --- | --- | --- | --- | --- |
| Eigen::Matrix<T,C,R> stan::math::transpose | ( | const Eigen::Matrix< T, R, C > & | *m* | ) |  |

inline

Definition at line 12 of file transpose.hpp.

template<typename T >

|  |  |  |  |  |  |
| --- | --- | --- | --- | --- | --- |
| T stan::math::trigamma | ( | T | *x* | ) |  |

inline

Definition at line 49 of file trigamma.hpp.

|  |  |  |  |
| --- | --- | --- | --- |
| void stan::math::validate\_non\_negative\_index | ( | const std::string & | *var\_name*, |
|  |  | const std::string & | *expr*, |
|  |  | int | *val* |
|  | ) |  |  |

inline

Definition at line 12 of file validate\_non\_negative\_index.hpp.

template<typename T >

|  |  |  |  |  |  |
| --- | --- | --- | --- | --- | --- |
| double stan::math::value\_of | ( | const T | *x* | ) |  |

inline

Return the value of the specified scalar argument converted to a double value.

See the `stan::math::primitive_value` function to extract values without casting to `double`.

This function is meant to cover the primitive types. For types requiring pass-by-reference, this template function should be specialized.

Template Parameters
:   |  |  |
    | --- | --- |
    | T | Type of scalar. |

Parameters
:   |  |  |
    | --- | --- |
    | x | Scalar to convert to double. |

Returns
:   Value of scalar cast to a double.

Definition at line 24 of file value\_of.hpp.

template<>

|  |  |  |  |  |  |
| --- | --- | --- | --- | --- | --- |
| double stan::math::value\_of< double > | ( | const double | *x* | ) |  |

inline

Return the specified argument.

See `value_of(T)` for a polymorphic implementation using static casts.

This inline pass-through no-op should be compiled away.

Parameters
:   |  |  |
    | --- | --- |
    | x | Specified value. |

Returns
:   Specified value.

Definition at line 40 of file value\_of.hpp.

template<typename T >

|  |  |  |  |  |  |
| --- | --- | --- | --- | --- | --- |
| boost::math::tools::promote\_args<T>::type stan::math::variance | ( | const std::vector< T > & | *v* | ) |  |

inline

Returns the sample variance (divide by length - 1) of the coefficients in the specified standard vector.

Parameters
:   |  |  |
    | --- | --- |
    | v | Specified vector. |

Returns
:   Sample variance of vector.

Exceptions
:   |  |  |
    | --- | --- |
    | std::domain\_error | if the size of the vector is less than 1. |

Definition at line 24 of file variance.hpp.

template<typename T , int R, int C>

|  |  |  |  |  |  |
| --- | --- | --- | --- | --- | --- |
| boost::math::tools::promote\_args<T>::type stan::math::variance | ( | const Eigen::Matrix< T, R, C > & | *m* | ) |  |

inline

Returns the sample variance (divide by length - 1) of the coefficients in the specified column vector.

Parameters
:   |  |  |
    | --- | --- |
    | m | Specified vector. |

Returns
:   Sample variance of vector.

Definition at line 46 of file variance.hpp.

## Variable Documentation

const double stan::math::CONSTRAINT\_TOLERANCE = 1E-8

The tolerance for checking arithmetic bounds In rank and in simplexes.

The default value is `1E-8`.

Definition at line 11 of file constraint\_tolerance.hpp.

const double stan::math::E = boost::math::constants::e<double>()

The base of the natural logarithm, .

Definition at line 14 of file constants.hpp.

const double stan::math::EPSILON = std::numeric\_limits<double>::epsilon()

Smallest positive value.

Definition at line 58 of file constants.hpp.

const double stan::math::INFTY = std::numeric\_limits<double>::infinity()

Positive infinity.

Definition at line 43 of file constants.hpp.

const double stan::math::INV\_SQRT\_2 = 1.0 / SQRT\_2

The value of 1 over the square root of 2, .

Definition at line 26 of file constants.hpp.

const double stan::math::INV\_SQRT\_TWO\_PI = 1.0 / std::sqrt(2.0 \* boost::math::constants::pi<double>())

Definition at line 150 of file constants.hpp.

const double stan::math::LOG\_10 = std::log(10.0)

The natural logarithm of 10, .

Definition at line 38 of file constants.hpp.

const double stan::math::LOG\_2 = std::log(2.0)

The natural logarithm of 2, .

Definition at line 32 of file constants.hpp.

const double stan::math::LOG\_PI\_OVER\_FOUR = std::log(boost::math::constants::pi<double>()) / 4.0

Definition at line 70 of file constants.hpp.

const double stan::math::NEG\_TWO\_OVER\_SQRT\_PI = -TWO\_OVER\_SQRT\_PI

Definition at line 148 of file constants.hpp.

const double stan::math::NEGATIVE\_EPSILON = - std::numeric\_limits<double>::epsilon()

Largest negative value (i.e., smallest absolute value).

Definition at line 63 of file constants.hpp.

const double stan::math::NEGATIVE\_INFTY = - std::numeric\_limits<double>::infinity()

Negative infinity.

Definition at line 48 of file constants.hpp.

const double stan::math::NOT\_A\_NUMBER = std::numeric\_limits<double>::quiet\_NaN()

(Quiet) not-a-number value.

Definition at line 53 of file constants.hpp.

const double stan::math::SQRT\_2 = std::sqrt(2.0)

The value of the square root of 2, .

Definition at line 20 of file constants.hpp.

const double stan::math::TWO\_OVER\_SQRT\_PI = 2.0 / std::sqrt(boost::math::constants::pi<double>())

Definition at line 146 of file constants.hpp.


---

[ Stan Home Page ]

*© 2011–2014,
Stan Development Team.*
